# Supplementary figures and images for: Identification and characterization of a target antigen recognized by the monoclonal antibody against Opisthorchis viverrini
Source: PLoS One. 2025 May 29;20(5):e0324137. doi: 10.1371/journal.pone.0324137 (PMC12121735; doi:10.1371/journal.pone.0324137)

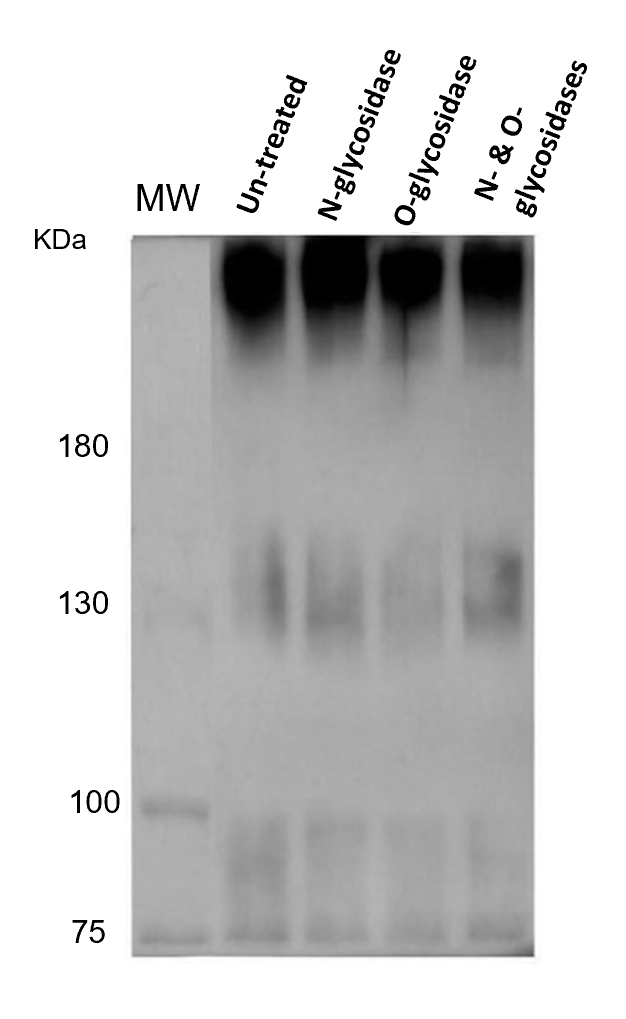

Supplement: S1 Fig — The proteins were separated using 6% acrylamide gels of SDS-PAGE follow by western blot analysis with the mAb KKU505 clone as a primary antibody. MW = standard protein molecular weights and KDa = kilodalton. (TIF) [file pone.0324137.s001.tif]

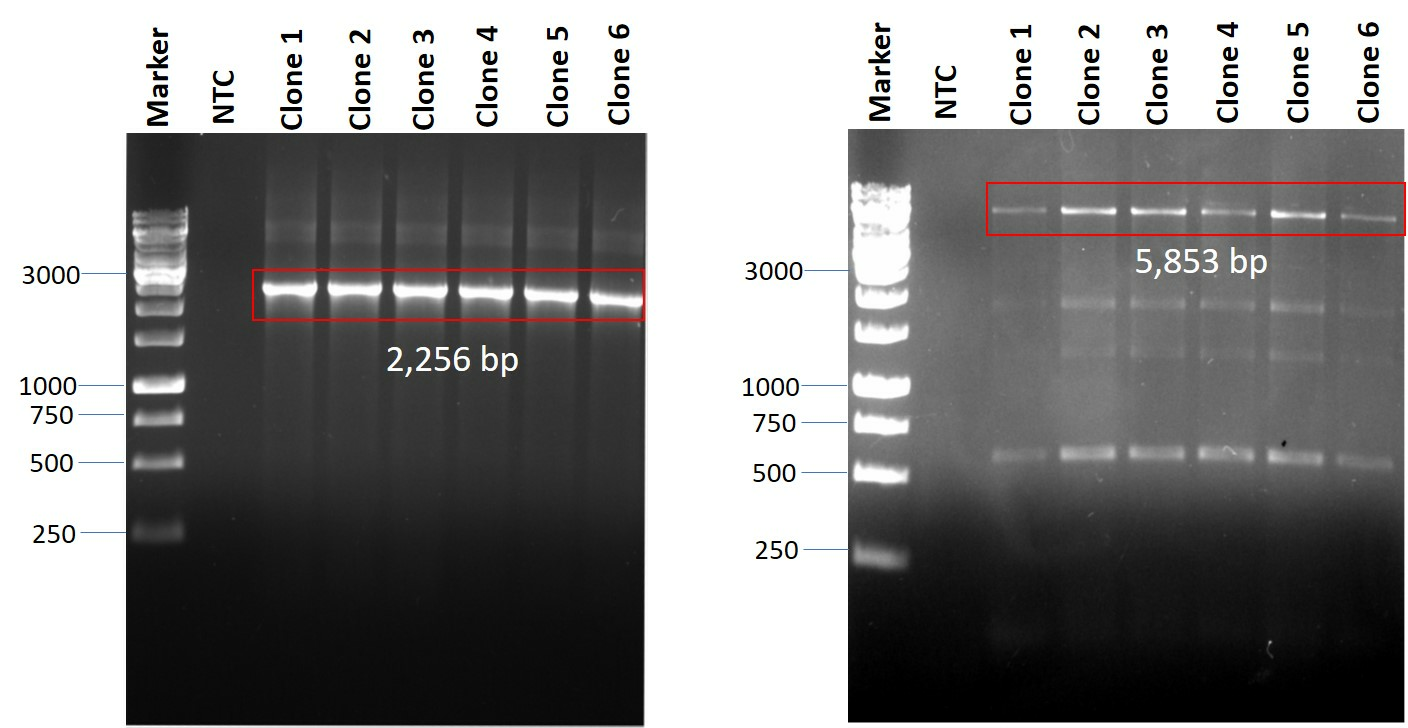

Supplement: S2 Fig — The 2,560 bp PCR products represents partial Ov myosin head inserted sequence vector and 5,853 bp PCR products represent full-length myosin heavy chain inserted sequence vector. NTC = Negative control. (TIF) [file pone.0324137.s002.tif]
