# Supplementary material for: Identification and characterization of a target antigen recognized by the monoclonal antibody against Opisthorchis viverrini
Source: PLoS One. 2025 May 29;20(5):e0324137. doi: 10.1371/journal.pone.0324137 (PMC12121735; doi:10.1371/journal.pone.0324137)
Supplement: S1 File — (PDF) [file pone.0324137.s003.pdf]

MATRIX SCIENCE Mascot Search Results

User	: tonkla
Email	: tonkla_ins@hotmail.com
Search title	: Ov 180 kDa-1
MS data file	: Crude-Ov 180 kD-1_E9_01_2104.mgf
Database	: NCBIprot 20180429 (152462470 sequences; 55858910152 residues)
Taxonomy	: Metazoa (Animals) (15843440 sequences)
Timestamp	: 23 Aug 2018 at 02:46:07 GMT
Enzyme	: Trypsin
Fixed modifications	: Carbamidomethyl (C)
Variable modifications	: Oxidation (HW),Oxidation (M)
Mass values	: Monoisotopic
Protein Mass	: Unrestricted
Peptide Mass Tolerance	: ± 0.5 Da
Fragment Mass Tolerance	: ± 0.5 Da
Max Missed Cleavages	: 1
Instrument type	: ESI-TRAP
Number of queries	: 1191
Protein hits	: OON15278.1 myosin head, partial [Opisthorchis viverrini]
	OON13874.1 immunoglobulin domain protein, partial [Opisthorchis viverrini]
	XP_009171936.1 hypothetical protein T265_07991 [Opisthorchis viverrini]
	CDS24615.1 myosin heavy chain [Echinococcus granulosus]
	GAA54660.1 innexin unc-9 [Clonorchis sinensis]
	XP_019577924.1 PREDICTED: malate dehydrogenase 2, mitochondrial [Rhinolophus sinicus]
	PIO33952.1 hypothetical protein AB205_0063410 [Rana catesbeiana]
	XP_003747528.1 PREDICTED: myosin heavy chain, muscle-like [Galendromus occidentalis]
	XP_018496589.1 PREDICTED: myosin heavy chain, muscle [Galendromus occidentalis]
	AAB03660.1 myosin heavy chain [Placopecten magellanicus]
	XP_023651856.1 myosin-7 [Paramormyrops kingsleyae]
	OON17488.1 ADP,ATP carrier protein 1 [Opisthorchis viverrini]
	XP_014293076.1 uncharacterized protein LOC106691733 [Halyomorpha halys]
	XP_018014352.1 PREDICTED: epithelial chloride channel protein-like [Hyalella azteca]
	XP_014661502.1 PREDICTED: myosin heavy chain, striated muscle-like isoform X1 [Priapulus caudatus]
	XP_002029795.1 GM24908 [Drosophila sechellia]
	OZC05323.1 hypothetical protein X798_07759 [Onchocerca flexuosa]
	XP_007459180.1 PREDICTED: LOW QUALITY PROTEIN: centrosomal protein of 85 kDa [Lipotes vexillifer]
	KFO85968.1 Myosin-3, partial [Buceros rhinoceros silvestris]
	XP_022596635.1 coiled-coil domain-containing protein 173 [Seriola dumerili]

Select Summary Report

Format As [Select Summary (protein hits)] Help

Significance threshold p< 0.05 Max. number of hits AUTO

Standard scoring (●) MudPIT scoring ( ) Display non-significant matches [ ] Show sub-sets 0

Show pop-ups (●) Suppress pop-ups ( ) Require bold red [ ]

Preferred taxonomy [All entries]

Re-Search (●) All queries ( ) Unassigned ( ) Below homology threshold ( ) Below identity threshold

1. OON15278.1 **Mass:** 222414 **Score:** 2484 **Matches:** 35(35) **Sequences:** 33(33) **emPAI:** 0.72

myosin head, partial [Opisthorchis viverrini]

Query	Observed	Mr(expt)	Mr(calc)	Delta	Miss	Score	Expect	Rank	Unique	Peptide
265	440.7630	879.5114	879.4702	0.0412	0	61	0.036	1		K.VGTEFVTK.G
400	508.8230	1015.6314	1015.5549	0.0765	0	63	0.029	1	U	K.ILEATNLDK.S
411	516.3830	1030.7514	1030.6175	0.1339	0	76	0.0015	1		K.VAFLLGVNAK.D
471	551.8370	1101.6594	1101.5601	0.0994	0	69	0.0077	1	U	K.MQGELQQLR.S
498	568.3040	1134.5934	1134.5040	0.0894	0	62	0.033	1	U	R.ESLEEEQSGK.A
522	582.3620	1162.7094	1162.5652	0.1442	0	73	0.0029	1	U	K.AGTLASLEDMR.D
551	594.8280	1187.6414	1187.5530	0.0884	0	63	0.026	1	U	R.NSQENAELQR.Q
566	601.8540	1201.6934	1201.5873	0.1061	0	84	0.0002	1	U	R.AQNEQAIAAMR.K
636	631.8650	1261.7154	1261.6038	0.1117	0	85	0.00019	1	U	K.DVEDLESSLQK.A
648	636.8570	1271.6994	1271.6469	0.0525	0	74	0.0018	1	U	K.QQLSAQLEEAR.H
651	638.8620	1275.7094	1275.5943	0.1152	0	69	0.0069	1	U	K.ATQETVDDLER.V
697	665.8690	1329.7234	1329.6161	0.1074	0	79	0.00067	1	U	R.EVQGQLEDEQR.Q
708	673.8850	1345.7554	1345.6361	0.1194	0	68	0.0089	1	U	R.ELETELEAEQR.R
715	675.4740	1348.9334	1348.7755	0.1580	0	75	0.0015	1	U	K.VISYFAIVAAAPK.K
739	690.3960	1378.7774	1378.6220	0.1554	0	87	9.8e-05	1	U	R.ANMMSGEIEEELR.T

752	703.8850	1405.7554	1405.6395	0.1160	0	74	0.0021	1	U	K.IQELEAECESAK.S
793	737.4190	1472.8234	1472.6994	0.1240	0	62	0.032	1	U	R.IQELEEDLEAER.A
802	745.4580	1488.9014	1488.7784	0.1231	0	68	0.0072	1	U	R.ATELSTQAASLAAQK.R
809	750.5220	1499.0294	1498.8871	0.1423	0	76	0.0011	1	U	K.LNVIIALFQAEIR.G
810	752.4330	1502.8514	1502.7576	0.0938	0	65	0.015	1		R.ENQSILITGESGAGK.T
851	779.4820	1556.9494	1556.8046	0.1449	0	60	0.044	1	U	K.ELEEQNVTVLQQK.N
858	781.9660	1561.9174	1561.7736	0.1438	0	67	0.0094	1	U	K.FEDEQSIVAQLQR.K
876	795.4870	1588.9594	1588.7944	0.1651	0	79	0.00054	1	U	R.QLEEAESQLSQLSK.V
896	810.9580	1619.9014	1619.7638	0.1376	0	83	0.00024	1	U	K.LDETTNQLSEQASGK.A
901	814.5070	1626.9994	1626.8464	0.1530	0	78	0.00076	1		R.QVEEAEEIAAINLAK.Y
912	823.4940	1644.9734	1644.8029	0.1706	0	103	2.3e-06	1	U	R.LQGELEDLMVDVER.A
913	825.4610	1648.9074	1648.7614	0.1461	0	87	9.5e-05	1		K.LESTLDEMEENLAR.E
929	835.9950	1669.9754	1669.8159	0.1596	0	87	9e-05	1	U	K.AEVDDLHSQLESLSK.A
934	839.0270	1676.0394	1675.8893	0.1501	0	107	8.9e-07	1	U	K.GQNLNQVTYAVSALAK.S
935	559.7040	1676.0902	1675.8893	0.2009	0	(61)	0.037	1	U	K.GQNLNQVTYAVSALAK.S
950	851.5030	1700.9914	1700.8581	0.1334	0	95	1.4e-05	1	U	R.TLQGEIAQQDEQITK.L
974	880.0640	1758.1134	1757.9564	0.1571	0	61	0.039	1	U	K.DALVSQLFVPVVAESGK.K
994	892.5570	1783.0994	1782.9476	0.1519	1	(65)	0.013	1	U	K.NKDPLNDTVVNLLGGSK.D
995	595.3860	1783.1362	1782.9476	0.1886	1	66	0.011	1	U	K.NKDPLNDTVVNLLGGSK.D
997	894.0270	1786.0394	1785.8745	0.1650	0	96	1.2e-05	1	U	R.LEEQDGVNAQQVDLTK.K

---

2. OON13874.1 **Mass:** 578548 **Score:** 491 **Matches:** 7(7) **Sequences:** 7(7) **emPAI:** 0.05
immunoglobulin domain protein, partial [Opisthorchis viverrini]

Query	Observed	Mr(expt)	Mr(calc)	Delta	Miss	Score	Expect	Rank	Unique	Peptide
460	544.8230	1087.6314	1087.5258	0.1057	0	65	0.019	1	U	R.NLDGGLADASR.L
502	570.8810	1139.7474	1139.5975	0.1499	0	77	0.00088	1	U	K.SEFFALAVTR.A
590	612.9650	1223.9154	1223.7489	0.1665	0	65	0.016	1	U	K.QLVLDSLVLPK.T
681	658.4050	1314.7954	1314.6528	0.1426	0	69	0.0064	1	U	R.QIQDVDTNAVGR.C
880	799.4800	1596.9454	1596.7936	0.1518	0	77	0.00088	1	U	R.LYWAQLNAASFDAK.G
904	817.5030	1632.9914	1632.8148	0.1767	0	75	0.0015	1	U	R.YGWQLANPVTEDLK.T
983	883.5850	1765.1554	1764.9298	0.2257	0	71	0.0035	1	U	R.INVYQSEPFILIDSK.R

Proteins matching the same set of peptides:
XP_009162503.1 **Mass:** 759012 **Score:** 490 **Matches:** 7(7) **Sequences:** 7(7)
hypothetical protein T265_00466 [Opisthorchis viverrini]

---

3. XP_009171936.1 **Mass:** 250184 **Score:** 347 **Matches:** 5(5) **Sequences:** 5(5) **emPAI:** 0.08
hypothetical protein T265_07991 [Opisthorchis viverrini]

Query	Observed	Mr(expt)	Mr(calc)	Delta	Miss	Score	Expect	Rank	Unique	Peptide
612	622.9020	1243.7894	1243.6561	0.1334	0	61	0.047	1	U	K.ANPILEAFGNAK.T
665	646.4190	1290.8234	1290.6819	0.1415	0	86	0.00014	1	U	K.AQVEFAVEAISK.S
736	687.9020	1373.7894	1373.6786	0.1108	0	68	0.0075	1	U	R.SESAAVAELNEVR.V
866	788.4010	1574.7874	1574.7424	0.0451	0	73	0.0022	1	U	R.VADLSSQLAEEEER.S
945	848.5390	1695.0634	1694.8555	0.2079	0	60	0.047	1	U	R.LPIYNDEIIEWYK.G

Proteins matching the same set of peptides:
OON14868.1 **Mass:** 235391 **Score:** 346 **Matches:** 5(5) **Sequences:** 5(5)
myosin head [Opisthorchis viverrini]

---

4. CDS24615.1 **Mass:** 224152 **Score:** 329 **Matches:** 4(4) **Sequences:** 4(4) **emPAI:** 0.07
myosin heavy chain [Echinococcus granulosus]

Query	Observed	Mr(expt)	Mr(calc)	Delta	Miss	Score	Expect	Rank	Unique	Peptide
411	516.3830	1030.7514	1030.6175	0.1339	0	76	0.0015	1		K.VAFLLGVNAK.D
810	752.4330	1502.8514	1502.7576	0.0938	0	65	0.015	1		R.ENQSILITGESGAGK.T
912	823.4940	1644.9734	1644.8029	0.1706	0	103	2.3e-06	1	U	R.LQGEIEDLMVDVER.A
913	825.4610	1648.9074	1648.7614	0.1461	0	87	9.5e-05	1		K.LESTLDEMEENLAR.E

Proteins matching the same set of peptides:
CDS33163.1 **Mass:** 224303 **Score:** 329 **Matches:** 4(4) **Sequences:** 4(4)
myosin heavy chain [Hymenolepis microstoma]
CDS41550.1 **Mass:** 224160 **Score:** 329 **Matches:** 4(4) **Sequences:** 4(4)
myosin heavy chain [Echinococcus multilocularis]
XP_024352951.1 **Mass:** 227397 **Score:** 329 **Matches:** 4(4) **Sequences:** 4(4)

Myosin heavy chain, striated muscle [Echinococcus granulosus]

---

5. GAA54660.1 **Mass:** 53023 **Score:** 238 **Matches:** 3(3) **Sequences:** 3(3) **emPAI:** 0.23

innexin unc-9 [Clonorchis sinensis]

Query	Observed	Mr(expt)	Mr(calc)	Delta	Miss	Score	Expect	Rank	Unique	Peptide
345	484.8170	967.6194	967.5015	0.1180	0	60	0.044	1	U	K.DFIDLFAK.F
779	727.9580	1453.9014	1453.7089	0.1925	0	75	0.0014	1	U	R.ADLDDFIDVYLR.R
823	761.9330	1521.8514	1521.7133	0.1381	0	103	2.6e-06	1	U	R.VAEFLEDMIDGQR.D

Proteins matching the same set of peptides:

XP_009165966.1 **Mass:** 112458 **Score:** 238 **Matches:** 3(3) **Sequences:** 3(3)

hypothetical protein T265_03269 [Opisthorchis viverrini]

---

6. XP_019577924.1 **Mass:** 35993 **Score:** 193 **Matches:** 3(3) **Sequences:** 3(3) **emPAI:** 0.35

PREDICTED: malate dehydrogenase 2, mitochondrial [Rhinolophus sinicus]

Query	Observed	Mr(expt)	Mr(calc)	Delta	Miss	Score	Expect	Rank	Unique	Peptide
584	610.4440	1218.8734	1218.6972	0.1762	0	61	0.037	1	U	K.LFGVTTLDVVR.A
601	617.3490	1232.6834	1232.5885	0.0950	0	62	0.03	1	U	R.TQDGGTEVVEAK.A
686	659.9320	1317.8494	1317.6929	0.1566	0	69	0.0067	1	U	R.DDLFNINAGIVK.N

---

7. PIO33952.1 **Mass:** 214635 **Score:** 149 **Matches:** 2(2) **Sequences:** 2(2) **emPAI:** 0.03

hypothetical protein AB205_0063410 [Rana catesbeiana]

Query	Observed	Mr(expt)	Mr(calc)	Delta	Miss	Score	Expect	Rank	Unique	Peptide
810	752.4330	1502.8514	1502.7576	0.0938	0	65	0.015	1		R.ENQSILITGESGAGK.T
912	823.4940	1644.9734	1644.8029	0.1706	0	84	0.00018	3	U	R.LQAEVEDLMVDVER.S

Proteins matching the same set of peptides:

XP_018413513.1 **Mass:** 223688 **Score:** 149 **Matches:** 2(2) **Sequences:** 2(2)

PREDICTED: myosin-1B-like [Nanorana parkeri]

EPY83625.1 **Mass:** 225659 **Score:** 147 **Matches:** 2(2) **Sequences:** 2(2)

myosin-3 [Camelus ferus]

XP_006216239.1 **Mass:** 224482 **Score:** 147 **Matches:** 2(2) **Sequences:** 2(2)

PREDICTED: LOW QUALITY PROTEIN: myosin-3 [Vicugna pacos]

XP_010964285.1 **Mass:** 224676 **Score:** 147 **Matches:** 2(2) **Sequences:** 2(2)

PREDICTED: myosin-3 [Camelus bactrianus]

XP_014411923.1 **Mass:** 224662 **Score:** 147 **Matches:** 2(2) **Sequences:** 2(2)

PREDICTED: myosin-3 [Camelus ferus]

XP_018084804.1 **Mass:** 223712 **Score:** 146 **Matches:** 2(2) **Sequences:** 2(2)

PREDICTED: myosin-4-like [Xenopus laevis]

---

8. XP_003747528.1 **Mass:** 222499 **Score:** 141 **Matches:** 2(2) **Sequences:** 2(2) **emPAI:** 0.03

PREDICTED: myosin heavy chain, muscle-like [Galendromus occidentalis]

Query	Observed	Mr(expt)	Mr(calc)	Delta	Miss	Score	Expect	Rank	Unique	Peptide
265	440.7630	879.5114	879.4372	0.0743	0	61	0.036	1		R.VGTEMVTK.G
876	795.4870	1588.9594	1588.7944	0.1651	0	79	0.00054	1		R.QLEEAESQISQLSK.Q

Proteins matching the same set of peptides:

XP_022711325.1 **Mass:** 183245 **Score:** 141 **Matches:** 2(2) **Sequences:** 2(2)

myosin heavy chain, muscle-like isoform X1 [Varroa jacobsoni]

XP_022711327.1 **Mass:** 182888 **Score:** 141 **Matches:** 2(2) **Sequences:** 2(2)

myosin heavy chain, muscle-like isoform X2 [Varroa jacobsoni]

---

9. XP_018496589.1 **Mass:** 223260 **Score:** 141 **Matches:** 2(2) **Sequences:** 2(2) **emPAI:** 0.03

PREDICTED: myosin heavy chain, muscle [Galendromus occidentalis]

Query	Observed	Mr(expt)	Mr(calc)	Delta	Miss	Score	Expect	Rank	Unique	Peptide
265	440.7630	879.5114	879.4702	0.0412	0	61	0.036	1		K.VGTEFVTK.G
876	795.4870	1588.9594	1588.7944	0.1651	0	79	0.00054	1		R.QLEEAESQISQLSK.Q

Proteins matching the same set of peptides:

OQR72717.1 **Mass:** 222906 **Score:** 139 **Matches:** 2(2) **Sequences:** 2(2)

myosin heavy chain [Tropilaelaps mercedesae]

XP_022709639.1 **Mass:** 444999 **Score:** 139 **Matches:** 2(2) **Sequences:** 2(2)

uncharacterized protein LOC111272448 [Varroa jacobsoni]

XP_022647816.1 **Mass:** 672553 **Score:** 135 **Matches:** 2(2) **Sequences:** 2(2)
LOW QUALITY PROTEIN: uncharacterized protein LOC111244705 [Varroa destructor]

---

10. AAB03660.1 **Mass:** 224190 **Score:** 139 **Matches:** 2(2) **Sequences:** 2(2) **emPAI:** 0.03
myosin heavy chain [Placopecten magellanicus]

Query	Observed	Mr(expt)	Mr(calc)	Delta	Miss	Score	Expect	Rank	Unique	Peptide
265	440.7630	879.5114	879.4372	0.0743	0	61	0.036	1		K.VGTEMVTK.G
901	814.5070	1626.9994	1626.8464	0.1530	0	78	0.00076	1		R.QVEEAEEIAAINLAK.Y

Proteins matching the same set of peptides:
AAB03661.1 **Mass:** 225130 **Score:** 139 **Matches:** 2(2) **Sequences:** 2(2)
myosin heavy chain [Placopecten magellanicus]
BAB40711.1 **Mass:** 224174 **Score:** 139 **Matches:** 2(2) **Sequences:** 2(2)
myosin [Mizuhopecten yessoensis]
OWF43611.1 **Mass:** 197791 **Score:** 139 **Matches:** 2(2) **Sequences:** 2(2)
Myosin heavy chain, striated muscle [Mizuhopecten yessoensis]
XP_021367464.1 **Mass:** 226207 **Score:** 139 **Matches:** 2(2) **Sequences:** 2(2)
myosin heavy chain, striated muscle isoform X1 [Mizuhopecten yessoensis]
XP_021367465.1 **Mass:** 226279 **Score:** 139 **Matches:** 2(2) **Sequences:** 2(2)
myosin heavy chain, striated muscle isoform X2 [Mizuhopecten yessoensis]
XP_021367466.1 **Mass:** 226147 **Score:** 139 **Matches:** 2(2) **Sequences:** 2(2)
myosin heavy chain, striated muscle isoform X3 [Mizuhopecten yessoensis]
XP_021367467.1 **Mass:** 226219 **Score:** 139 **Matches:** 2(2) **Sequences:** 2(2)
myosin heavy chain, striated muscle isoform X4 [Mizuhopecten yessoensis]
XP_021367468.1 **Mass:** 226203 **Score:** 139 **Matches:** 2(2) **Sequences:** 2(2)
myosin heavy chain, striated muscle isoform X5 [Mizuhopecten yessoensis]
XP_021367469.1 **Mass:** 226143 **Score:** 139 **Matches:** 2(2) **Sequences:** 2(2)
myosin heavy chain, striated muscle isoform X6 [Mizuhopecten yessoensis]
XP_021367470.1 **Mass:** 226114 **Score:** 139 **Matches:** 2(2) **Sequences:** 2(2)
myosin heavy chain, striated muscle isoform X7 [Mizuhopecten yessoensis]
XP_021367471.1 **Mass:** 224794 **Score:** 139 **Matches:** 2(2) **Sequences:** 2(2)
myosin heavy chain, striated muscle isoform X8 [Mizuhopecten yessoensis]
XP_021367472.1 **Mass:** 224734 **Score:** 139 **Matches:** 2(2) **Sequences:** 2(2)
myosin heavy chain, striated muscle isoform X9 [Mizuhopecten yessoensis]
XP_021367473.1 **Mass:** 223985 **Score:** 139 **Matches:** 2(2) **Sequences:** 2(2)
myosin heavy chain, striated muscle isoform X10 [Mizuhopecten yessoensis]
XP_021367474.1 **Mass:** 224641 **Score:** 139 **Matches:** 2(2) **Sequences:** 2(2)
myosin heavy chain, striated muscle isoform X11 [Mizuhopecten yessoensis]
XP_021367475.1 **Mass:** 226275 **Score:** 139 **Matches:** 2(2) **Sequences:** 2(2)
myosin heavy chain, striated muscle isoform X12 [Mizuhopecten yessoensis]
P24733.1 **Mass:** 223824 **Score:** 137 **Matches:** 2(2) **Sequences:** 2(2)
RecName: Full=Myosin heavy chain, striated muscle
AAC46490.1 **Mass:** 225198 **Score:** 137 **Matches:** 2(2) **Sequences:** 2(2)
myosin heavy chain [Argopecten irradians]
AAD52842.1 **Mass:** 224145 **Score:** 137 **Matches:** 2(2) **Sequences:** 2(2)
myosin heavy chain [Pecten maximus]

---

11. XP_023651856.1 **Mass:** 224575 **Score:** 133 **Matches:** 2(2) **Sequences:** 2(2) **emPAI:** 0.03
myosin-7 [Paramormyrops kingsleyae]

Query	Observed	Mr(expt)	Mr(calc)	Delta	Miss	Score	Expect	Rank	Unique	Peptide
708	673.8850	1345.7554	1345.6361	0.1194	0	68	0.0089	1	U	R.ELETEIEAEQR.R
810	752.4330	1502.8514	1502.7576	0.0938	0	65	0.015	1		R.ENQSILITGESGAGK.T

---

12. OON17488.1 **Mass:** 34543 **Score:** 74 **Matches:** 1(1) **Sequences:** 1(1) **emPAI:** 0.11
ADP,ATP carrier protein 1 [Opisthorchis viverrini]

Query	Observed	Mr(expt)	Mr(calc)	Delta	Miss	Score	Expect	Rank	Unique	Peptide
507	**574.3650**	**1146.7154**	**1146.6033**	**0.1121**	**0**	**74**	**0.0022**	**1**	**U**	**R.GVAGGGVLAGFDK.F**

Proteins matching the same set of peptides:
PIS80496.1 **Mass:** 34360 **Score:** 74 **Matches:** 1(1) **Sequences:** 1(1)
ADP,ATP carrier protein 1 [Fasciola hepatica]
ABU86405.1 **Mass:** 16498 **Score:** 74 **Matches:** 1(1) **Sequences:** 1(1)

ADP/ATP carrier, partial [Clonorchis sinensis]

XP_009162187.1 **Mass:** 72085 **Score:** 74 **Matches:** 1(1) **Sequences:** 1(1)

hypothetical protein T265_00215 [Opisthorchis viverrini]

---

13. XP_014293076.1 **Mass:** 67867 **Score:** 72 **Matches:** 1(1) **Sequences:** 1(1) **emPAI:** 0.05

uncharacterized protein LOC106691733 [Halyomorpha halys]

Query	Observed	Mr(expt)	Mr(calc)	Delta	Miss	Score	Expect	Rank	Unique	Peptide
387	**503.8310**	**1005.6474**	**1005.5529**	**0.0946**	**0**	**72**	**0.0038**	**1**	**U**	**R.GSTALMIAVK.N**

---

14. XP_018014352.1 **Mass:** 125732 **Score:** 65 **Matches:** 1(1) **Sequences:** 1(1) **emPAI:** 0.03

PREDICTED: epithelial chloride channel protein-like [Hyalella azteca]

Query	Observed	Mr(expt)	Mr(calc)	Delta	Miss	Score	Expect	Rank	Unique	Peptide
246	**428.7730**	**855.5314**	**855.5178**	**0.0137**	**0**	**65**	**0.013**	**1**	**U**	**R.LATVSLPR.T**

---

15. XP_014661502.1 **Mass:** 225375 **Score:** 65 **Matches:** 1(1) **Sequences:** 1(1) **emPAI:** 0.02

PREDICTED: myosin heavy chain, striated muscle-like isoform X1 [Priapulus caudatus]

Query	Observed	Mr(expt)	Mr(calc)	Delta	Miss	Score	Expect	Rank	Unique	Peptide
810	752.4330	1502.8514	1502.7576	0.0938	0	65	0.015	1	U	R.ENQSLLITGESGAGK.T

Proteins matching the same set of peptides:

XP_017351886.1 **Mass:** 224307 **Score:** 63 **Matches:** 1(1) **Sequences:** 1(1)

PREDICTED: myosin-7-like [Ictalurus punctatus]

---

16. XP_002029795.1 **Score:** 63 **Matches:** 1(1) **Sequences:** 1(1) **emPAI:** 0.07

GM24908 [Drosophila sechellia]

Query	Observed	Mr(expt)	Mr(calc)	Delta	Miss	Score	Expect	Rank	Unique	Peptide
471	551.8370	1101.6594	1101.5601	0.0994	0	63	0.028	2	U	-.MQADLQQLR.N

---

17. OZC05323.1 **Mass:** 78287 **Score:** 61 **Matches:** 1(1) **Sequences:** 1(1) **emPAI:** 0.05

hypothetical protein X798_07759 [Onchocerca flexuosa]

Query	Observed	Mr(expt)	Mr(calc)	Delta	Miss	Score	Expect	Rank	Unique	Peptide
584	610.4440	1218.8734	1218.6972	0.1762	0	61	0.037	1	U	R.IFGVTTLDVVR.S

Proteins matching the same set of peptides:

KPM12024.1 **Mass:** 39487 **Score:** 61 **Matches:** 1(1) **Sequences:** 1(1)

malate dehydrogenase, mitochondrial-like protein, partial [Sarcoptes scabiei]

ACZ13336.1 **Mass:** 35241 **Score:** 61 **Matches:** 1(1) **Sequences:** 1(1)

malate dehydrogenase [Bursaphelenchus xylophilus]

EPQ15740.1 **Mass:** 34535 **Score:** 61 **Matches:** 1(1) **Sequences:** 1(1)

Malate dehydrogenase, mitochondrial [Myotis brandtii]

AAN23843.1 **Mass:** 24212 **Score:** 61 **Matches:** 1(1) **Sequences:** 1(1)

mitochondrial malate dehydrogenase precursor, partial [Phorcus lineatus]

CRZ25392.1 **Mass:** 40440 **Score:** 61 **Matches:** 1(1) **Sequences:** 1(1)

BMA-MDH-2 [Brugia malayi]

ABO26695.1 **Mass:** 26016 **Score:** 61 **Matches:** 1(1) **Sequences:** 1(1)

malate dehydrogenase precursor, partial [Haliotis discus discus]

AFJ54472.1 **Mass:** 22508 **Score:** 61 **Matches:** 1(1) **Sequences:** 1(1)

mitochondrial malate dehydrogenase 2, partial [Miniopterus schreibersii]

AFJ54473.1 **Mass:** 25483 **Score:** 61 **Matches:** 1(1) **Sequences:** 1(1)

mitochondrial malate dehydrogenase 2, partial [Rhinolophus macrotis]

AFJ54474.1 **Mass:** 23226 **Score:** 61 **Matches:** 1(1) **Sequences:** 1(1)

mitochondrial malate dehydrogenase 2, partial [Pipistrellus sp. YPZ-2012]

AFJ54475.1 **Mass:** 23041 **Score:** 61 **Matches:** 1(1) **Sequences:** 1(1)

mitochondrial malate dehydrogenase 2, partial [Rhinolophus paradoxolophus]

AFJ54476.1 **Mass:** 23038 **Score:** 61 **Matches:** 1(1) **Sequences:** 1(1)

mitochondrial malate dehydrogenase 2, partial [Rhinolophus rex]

AFJ54477.1 **Mass:** 25354 **Score:** 61 **Matches:** 1(1) **Sequences:** 1(1)

mitochondrial malate dehydrogenase 2, partial [Ia io]

AFJ54478.1 **Mass:** 25366 **Score:** 61 **Matches:** 1(1) **Sequences:** 1(1)

mitochondrial malate dehydrogenase 2, partial [Rhinolophus stheno]

AFJ54479.1 **Mass:** 25378 **Score:** 61 **Matches:** 1(1) **Sequences:** 1(1)

mitochondrial malate dehydrogenase 2, partial [Hipposideros larvatus]

PIO67669.1 **Mass:** 33617 **Score:** 61 **Matches:** 1(1) **Sequences:** 1(1)
malate dehydrogenase, NAD-dependent [Teladorsagia circumcincta]
OQR67796.1 **Mass:** 35406 **Score:** 61 **Matches:** 1(1) **Sequences:** 1(1)
malate dehydrogenase [Tropilaelaps mercedesae]
OBS68549.1 **Mass:** 25373 **Score:** 61 **Matches:** 1(1) **Sequences:** 1(1)
hypothetical protein A6R68_02912 [Neotoma lepida]
PIO69504.1 **Mass:** 36083 **Score:** 61 **Matches:** 1(1) **Sequences:** 1(1)
malate dehydrogenase, NAD-dependent [Teladorsagia circumcincta]
OTF70849.1 **Mass:** 36300 **Score:** 61 **Matches:** 1(1) **Sequences:** 1(1)
malate dehydrogenase, mitochondrial-like protein [Euroglyphus maynei]
ABD77282.1 **Mass:** 19538 **Score:** 61 **Matches:** 1(1) **Sequences:** 1(1)
mitochondrial malate dehydrogenase 2, NAD, partial [Tamandua tetradactyla]
ABD77289.1 **Mass:** 30986 **Score:** 61 **Matches:** 1(1) **Sequences:** 1(1)
mitochondrial malate dehydrogenase 2, NAD, partial [Tadarida brasiliensis]
CDJ82700.1 **Mass:** 39903 **Score:** 61 **Matches:** 1(1) **Sequences:** 1(1)
Lactate malate dehydrogenase domain containing protein [Haemonchus contortus]
ELT94561.1 **Mass:** 36841 **Score:** 61 **Matches:** 1(1) **Sequences:** 1(1)
hypothetical protein CAPTEDRAFT_159451 [Capitella teleta]
XP_792004.2 **Mass:** 35691 **Score:** 61 **Matches:** 1(1) **Sequences:** 1(1)
PREDICTED: malate dehydrogenase, mitochondrial [Strongylocentrotus purpuratus]
XP_001900755.1 **Mass:** 36263 **Score:** 61 **Matches:** 1(1) **Sequences:** 1(1)
Probable malate dehydrogenase, mitochondrial precursor , putative [Brugia malayi]
XP_003137632.1 **Mass:** 36359 **Score:** 61 **Matches:** 1(1) **Sequences:** 1(1)
malate dehydrogenase [Loa loa]
XP_003741774.1 **Mass:** 35344 **Score:** 61 **Matches:** 1(1) **Sequences:** 1(1)
PREDICTED: malate dehydrogenase, mitochondrial [Galendromus occidentalis]
XP_006088317.1 **Mass:** 35982 **Score:** 61 **Matches:** 1(1) **Sequences:** 1(1)
malate dehydrogenase, mitochondrial isoform X1 [Myotis lucifugus]
XP_006773447.1 **Mass:** 24924 **Score:** 61 **Matches:** 1(1) **Sequences:** 1(1)
PREDICTED: malate dehydrogenase, mitochondrial [Myotis davidii]
XP_008139680.1 **Mass:** 35969 **Score:** 61 **Matches:** 1(1) **Sequences:** 1(1)
PREDICTED: malate dehydrogenase, mitochondrial isoform X1 [Eptesicus fuscus]
XP_009021600.1 **Mass:** 39022 **Score:** 61 **Matches:** 1(1) **Sequences:** 1(1)
hypothetical protein HELRODRAFT_185773 [Helobdella robusta]
XP_009865113.1 **Mass:** 39191 **Score:** 61 **Matches:** 1(1) **Sequences:** 1(1)
PREDICTED: LOW QUALITY PROTEIN: malate dehydrogenase, mitochondrial [Apaloderma vittatum]
XP_011369846.1 **Mass:** 36000 **Score:** 61 **Matches:** 1(1) **Sequences:** 1(1)
malate dehydrogenase, mitochondrial [Pteropus vampyrus]
XP_014233158.1 **Mass:** 35884 **Score:** 61 **Matches:** 1(1) **Sequences:** 1(1)
malate dehydrogenase, mitochondrial [Trichogramma pretiosum]
XP_015927425.1 **Mass:** 36001 **Score:** 61 **Matches:** 1(1) **Sequences:** 1(1)
malate dehydrogenase, mitochondrial-like [Parasteatoda tepidariorum]
XP_016002032.1 **Mass:** 36014 **Score:** 61 **Matches:** 1(1) **Sequences:** 1(1)
PREDICTED: malate dehydrogenase, mitochondrial [Rousettus aegyptiacus]
XP_016054490.1 **Mass:** 36032 **Score:** 61 **Matches:** 1(1) **Sequences:** 1(1)
PREDICTED: malate dehydrogenase, mitochondrial [Miniopterus natalensis]
XP_017777259.1 **Mass:** 35746 **Score:** 61 **Matches:** 1(1) **Sequences:** 1(1)
PREDICTED: malate dehydrogenase, mitochondrial [Nicrophorus vespilloides]
XP_018319136.1 **Mass:** 35696 **Score:** 61 **Matches:** 1(1) **Sequences:** 1(1)
PREDICTED: malate dehydrogenase, mitochondrial [Agrilus planipennis]
XP_019502404.1 **Mass:** 36084 **Score:** 61 **Matches:** 1(1) **Sequences:** 1(1)
PREDICTED: malate dehydrogenase, mitochondrial [Hipposideros armiger]
XP_019573231.1 **Mass:** 36043 **Score:** 61 **Matches:** 1(1) **Sequences:** 1(1)
PREDICTED: malate dehydrogenase, mitochondrial [Rhinolophus sinicus]
XP_020373996.1 **Mass:** 35801 **Score:** 61 **Matches:** 1(1) **Sequences:** 1(1)
LOW QUALITY PROTEIN: malate dehydrogenase, mitochondrial [Rhincodon typus]
XP_022660414.1 **Mass:** 35696 **Score:** 61 **Matches:** 1(1) **Sequences:** 1(1)
malate dehydrogenase, mitochondrial-like [Varroa destructor]
XP_023222559.1 **Mass:** 36018 **Score:** 61 **Matches:** 1(1) **Sequences:** 1(1)
malate dehydrogenase, mitochondrial-like [Centruroides sculpturatus]
XP_023609246.1 **Mass:** 24938 **Score:** 61 **Matches:** 1(1) **Sequences:** 1(1)
malate dehydrogenase, mitochondrial isoform X2 [Myotis lucifugus]
XP_024427292.1 **Mass:** 36114 **Score:** 61 **Matches:** 1(1) **Sequences:** 1(1)
malate dehydrogenase, mitochondrial [Desmodus rotundus]
KZS17437.1 **Mass:** 36051 **Score:** 60 **Matches:** 1(1) **Sequences:** 1(1)

Malate dehydrogenase, mitochondrial [Daphnia magna]

PDM84942.1 **Mass:** 236221 **Score:** 60 **Matches:** 1(1) **Sequences:** 1(1)

glycoside hydrolase [Pristionchus pacificus]

---

18. XP_007459180.1 **Mass:** 87126 **Score:** 61 **Matches:** 1(1) **Sequences:** 1(1) **emPAI:** 0.04

PREDICTED: LOW QUALITY PROTEIN: centrosomal protein of 85 kDa [Lipotes vexillifer]

Query	Observed	Mr(expt)	Mr(calc)	Delta	Miss	Score	Expect	Rank	Unique	Peptide
665	646.4190	1290.8234	1290.6932	0.1303	1	61	0.039	2	U	R.AQFAQKAEALSK.E

---

19. KFO85968.1 **Mass:** 45949 **Score:** 61 **Matches:** 1(1) **Sequences:** 1(1) **emPAI:** 0.08

Myosin-3, partial [Buceros rhinoceros silvestris]

Query	Observed	Mr(expt)	Mr(calc)	Delta	Miss	Score	Expect	Rank	Unique	Peptide
612	622.9020	1243.7894	1243.6561	0.1334	0	61	0.047	1	U	-.ANPLLEAFGNAK.T

Proteins matching the same set of peptides:

XP_009927788.1 **Mass:** 49952 **Score:** 61 **Matches:** 1(1) **Sequences:** 1(1)

PREDICTED: myosin heavy chain, skeletal muscle, adult-like, partial [Haliaeetus albicilla]

---

20. XP_022596635.1 **Mass:** 64472 **Score:** 59 **Matches:** 1(1) **Sequences:** 1(1) **emPAI:** 0.06

coiled-coil domain-containing protein 173 [Seriola dumerili]

Query	Observed	Mr(expt)	Mr(calc)	Delta	Miss	Score	Expect	Rank	Unique	Peptide
470	**551.3240**	**1100.6334**	**1100.5826**	**0.0509**	**1**	**61**	**0.043**	**1**	**U**	**R.QEQEKALQK.K**

Proteins matching the same set of peptides:

XP_023259287.1 **Mass:** 61881 **Score:** 59 **Matches:** 1(1) **Sequences:** 1(1)

coiled-coil domain-containing protein 173 [Seriola lalandi dorsalis]

Mascot: http://www.matrixscience.com/
