# Supplementary material for: Identification and characterization of a target antigen recognized by the monoclonal antibody against Opisthorchis viverrini
Source: PLoS One. 2025 May 29;20(5):e0324137. doi: 10.1371/journal.pone.0324137 (PMC12121735; doi:10.1371/journal.pone.0324137)
Supplement: S2 File — (PDF) [file pone.0324137.s004.pdf]

MATRIX SCIENCE MASCOT Search Results

Protein View: OON15278.1

myosin head, partial [Opisthorchis viverrini]

**Database:**	NCBIprot
**Score:**	2010
**Monoisotopic mass (M_r):**	222414
**Calculated pI:**	5.57
**Taxonomy:**	Opisthorchis viverrini

Sequence similarity is available as an NCBI BLAST search of OON15278.1 against nr.

Search parameters

**MS data file:**	Full Myosin_H2_01_2402.mgf
**Enzyme:**	Trypsin: cuts C-term side of KR unless next residue is P.
**Fixed modifications:**	Carbamidomethyl (C)
**Variable modifications:**	Oxidation (M), Oxidation (HW)

Protein sequence coverage: 13%

Matched peptides shown in ***bold red***.

1 MEPNDPDFKY LGVDRKALLK ELANFDSKNV IWVEDEK**EGY** **ILADIVETSG**

51 **DTITVK**LKDG VEKKVKK**DDA** **QQVNPPK**FFL IEDMANLTFL NDASVLENLR

101 ARYYRQLIYT YSGLFCVAVN PYK**RFPIYTA** **QVALK**YKGKR RSEMPPHIFS

151 ISDNAYHNML QDR**ENQSILI** **TGESGAGK**TE NTK**KVISYFA** **IVAAAPK**KDD

201 DESSK**KGSLE** **DQIVQANPVL** **EAYGNAK**TTR NNNSSRFGKF IRIHFGTTGK

251 **IAGADIEHYL** **LEK**SRVVSQM KGERNYHIFY QLLSDYGKKY HDKLLVAADP

301 GLYSFINQGE LTIDGVDDSE EMR**LCDEAFE** **VLGFSEEEK**M SLYK**CTTSIC**

351 **NLGEMK**FKQR PR**EEQAEADG** **TAECEKVAFL** **LGVNAKDLMT** **SFLKPK**VK**VG**

401 **TEFVTKGQNL** **NQVTYAVSAL** **AK**SLYNR**MFG** **WLVAR**VNKTL DTKVKRQFFI

451 GVLDIAGFEI FDENGFEQIC INYTNERLQQ FFNHHMFVLE QEEYKREKIQ

501 WTFIDFGMDL QACIDLIEKP MGILSILEEE CIVPK**ATDQT** **FLSK**LYDNHL

551 GKSPNFTKPK PPKAGQKEAH FELHHYAGSV PYTITGWLEK **NKDPLNDTVV**

601 **NLLGGSKDAL** **VSQLFVPVVA** **ESGK**KTKGGS FLTVSFMHRE SLNKLMKNLH

651 STSPHFIRCI VPNEFKQPGV VDAHLVLHQL HCNGVLEGIR ICRKGFPNRM

701 IYSEFKQR**YS** **ILAPNVIPEG** **FVDGR**QVTEK **ILEATNLDK**S LYQCGNTKVF

751 FKAGTLASLE DMRDEKLNVI IALFQAEIRG YLMRKQYKKL QDQRVALTLM

801 QRNIRKYLML RNWPWWRLYA KVKPMLNVAR QEEEMKKAAE ELAKLKEEFE

851 KLEKLKKELE EQNVTVLQQK NDLFLQLQTE QDSLAEAEEK ISNLVLQRGD

901 LETRIKELEE RLADEEDANS NLNEMKKKLN AEIDELKKDV EDLESSLQKA

951 EQEKQTKDNQ IRTLQGEIAQ QDEQITKLNK DKKALEEQNK RTQEALQAEE

1001 DKVNHLNKLK AKLESTLDEM EENLAREQKV RADVEKAKRK LEGDLKATQE

1051 TVDDLERVKR DLEEQLRRRE VEIGGLNSKF EDEQSIVAQL QRKIKELQTR

1101 IQELEEDLEA ERAARSKAEK ARQQASHVEA ELEEVVERLE EQDGVNAQQV

1151 DLTKKRESEL MKLKRDLEDA RAQNEQAIAA MRKKQNDSVN EMADQLDQLN

1201 KAKAKAEKER NQFKAEVDDL HSQLESLSKA KMNAEKNTKG LETQIQELHA

1251 KLDETTNQLS EQASGKARNS QENAELQRQL EEAESQLSQL SKVKQQLSAQ

1301 LEEARHSLEE ESRMKAKLNS EVRNLTSDLD SLRESLEEEQ SGKADLQRQL

1351 QKMQGELQQL RSRGGGDVR SEEMEELKRR LNGKIQELEA ECESAKSKCG

1401 QLEKTKARLQ GELEDLMVDV ERANGMASQL ERKQANFNRT LAEWQKKFAD

1451 SQAELENSQR DARNQSTEMF RLKAQLEEAH EQIEAVRREN KNLSDEIHDL

1501 TEQLGEGGRS VHEIDKARRR LEMEKEELQA ALEEAEGALE QEEAKLQRAQ

1551 LEMSQVRQEI DRRLAEKEEE FEATRKNHQR AMESQQASLE AEAKGKAEAL

1601 RIKKKLEQDI NELEVSLDAA NRNRAEQEKN VKKLQQQVRE VQGQLEDEQR

1651 QRDDIREQFQ AAERRANMMS GEIEELRTAL EQAERGRKMA EAERAEAADR

1701 ATELSTQAAS LAAQKRKLEA DLAAMQADLE EAANEAKQAD ERAKKAMADS

1751 SRVFEEIRQE QEHTQHVEKA RKQLELQVKE MQARLEDTES GAAKNGRKAV

1801 GKLEQRVREL ETELEAEQRR HGETLKNLRK VDRRMKEISM QSEEDKKNHE

1851 RMQELVEKLQ GKIKTYKRQV EEAEEIAAIN LAKYRKIQHE IEDAEERADQ

1901 AEQALQKLRA KNRSSVSAAR GVSAAPAGAG PA

Unformatted sequence string: 1932 residues (for pasting into other applications).

Sort by (●) residue number ( ) increasing mass ( ) decreasing mass

Show (●) matched peptides only ( ) predicted peptides also

Query	Start – End	Observed	Mr(expt)	Mr(calc)	Delta	M	Score	Expect	Rank	U	Peptide
1007	38 – 56	1012.5850	2023.1554	2023.0361	0.1193	0	70	0.0011	1	U	K.EGYILADIVETSGDTITVK.L
501	68 – 77	556.3120	1110.6094	1110.5305	0.0789	0	57	0.03	1	U	K.DDAQQVNPPK.F
713	124 – 135	469.6390	1405.8952	1405.8082	0.0870	1	55	0.038	1	U	K.RFPIYTAQVALK.Y
563	125 – 135	625.9220	1249.8294	1249.7070	0.1224	0	68	0.0025	1	U	R.FPIYTAQVALK.Y
564	125 – 135	625.9280	1249.8414	1249.7070	0.1344	0	70	0.0013	1	U	R.FPIYTAQVALK.Y
769	164 – 178	501.9600	1502.8582	1502.7576	0.1005	0	58	0.023	1	U	R.ENQSILITGESGAGK.T

Query	Start – End	Observed	Mr(expt)	Mr(calc)	Delta	M	Score	Expect	Rank	U	Peptide
770	164 – 178	752.4370	1502.8594	1502.7576	0.1018	0	112	8.6e-08	1	U	R.ENQSILITGESGAGK.T
771	164 – 178	752.4400	1502.8654	1502.7576	0.1078	0	67	0.0026	1	U	R.ENQSILITGESGAGK.T
757	184 – 197	739.4610	1476.9074	1476.8704	0.0370	1	78	0.00023	1	U	K.KVISYFAIVAAAPK.K
659	185 – 197	450.6160	1348.8262	1348.7755	0.0507	0	59	0.018	1	U	K.VISYFAIVAAAPK.K
660	185 – 197	675.4430	1348.8714	1348.7755	0.0960	0	56	0.032	1	U	K.VISYFAIVAAAPK.K
662	185 – 197	675.4600	1348.9054	1348.7755	0.1300	0	88	2.4e-05	1	U	K.VISYFAIVAAAPK.K
663	185 – 197	675.4620	1348.9094	1348.7755	0.1340	0	59	0.019	1	U	K.VISYFAIVAAAPK.K
664	185 – 197	675.4630	1348.9114	1348.7755	0.1360	0	62	0.0094	1	U	K.VISYFAIVAAAPK.K
665	185 – 197	675.4640	1348.9134	1348.7755	0.1380	0	55	0.046	1	U	K.VISYFAIVAAAPK.K
1032	206 – 227	782.1270	2343.3592	2343.2070	0.1522	1	73	0.00047	1	U	K.KGSLEDQIVQANPVLEAYGNAK.T
1023	207 – 227	739.4370	2215.2892	2215.1120	0.1771	0	77	0.00019	1	U	K.GSLEDQIVQANPVLEAYGNAK.T
1025	207 – 227	739.4460	2215.3162	2215.1120	0.2041	0	77	0.00022	1	U	K.GSLEDQIVQANPVLEAYGNAK.T
751	251 – 263	736.4390	1470.8634	1470.7718	0.0916	0	81	0.00011	1	U	K.IAGADIEHYLLEK.S
964	324 – 339	634.6970	1901.0692	1900.8400	0.2291	0	75	0.00039	1	U	R.LCDEAFEVLGFSEEEK.M
965	324 – 339	951.5590	1901.1034	1900.8400	0.2634	0	124	4.6e-09	1	U	R.LCDEAFEVLGFSEEEK.M
721	345 – 356	707.3700	1412.7254	1412.6098	0.1156	0	68	0.0023	1	U	K.CTTSICNLGEMK.F
732	345 – 356	715.3540	1428.6934	1428.6047	0.0887	0	67	0.0027	1	U	K.CTTSICNLGEMK.F + Oxidation (M)
797	363 – 376	783.8670	1565.7194	1565.6151	0.1043	0	75	0.0004	1	U	R.EEQAEADGTAECEK.V
451	377 – 386	516.3580	1030.7014	1030.6175	0.0839	0	92	1.1e-05	1		K.VAFLLGVNAK.D
523	387 – 396	590.3730	1178.7314	1178.6369	0.0945	0	55	0.047	1	U	K.DLMTSFLKPK.V
312	399 – 406	440.7460	879.4774	879.4702	0.0072	0	71	0.0013	1		K.VGTEFVTK.G
313	399 – 406	440.7510	879.4874	879.4702	0.0172	0	65	0.0041	1		K.VGTEFVTK.G
314	399 – 406	440.7520	879.4894	879.4702	0.0192	0	60	0.014	2		K.VGTEFVTK.G
850	407 – 422	838.9930	1675.9714	1675.8893	0.0821	0	95	4.4e-06	1	U	K.GQNLNQVTYAVSALAK.S
852	407 – 422	559.6770	1676.0092	1675.8893	0.1199	0	62	0.0088	1	U	K.GQNLNQVTYAVSALAK.S
853	407 – 422	839.0150	1676.0154	1675.8893	0.1261	0	141	1e-10	1	U	K.GQNLNQVTYAVSALAK.S
854	407 – 422	559.6870	1676.0392	1675.8893	0.1499	0	76	0.00035	1	U	K.GQNLNQVTYAVSALAK.S
855	407 – 422	559.6930	1676.0572	1675.8893	0.1679	0	66	0.0035	1	U	K.GQNLNQVTYAVSALAK.S
419	428 – 435	498.3060	994.5974	994.5059	0.0916	0	61	0.011	1	U	R.MFGWLVAR.V + Oxidation (M)
428	536 – 544	505.7830	1009.5514	1009.5080	0.0434	0	82	8.5e-05	1	U	K.ATDQTFLSK.L
429	536 – 544	505.7840	1009.5534	1009.5080	0.0454	0	67	0.0029	1	U	K.ATDQTFLSK.L
431	536 – 544	505.7900	1009.5654	1009.5080	0.0574	0	68	0.0022	1	U	K.ATDQTFLSK.L
898	591 – 607	595.3720	1783.0942	1782.9476	0.1466	1	63	0.0071	1	U	K.NKDPLNDTVVNLLGGSK.D
899	591 – 607	892.5570	1783.0994	1782.9476	0.1519	1	96	3.6e-06	1	U	K.NKDPLNDTVVNLLGGSK.D
900	591 – 607	595.3750	1783.1032	1782.9476	0.1556	1	69	0.0018	1	U	K.NKDPLNDTVVNLLGGSK.D
785	593 – 607	771.4780	1540.9414	1540.8097	0.1318	0	101	1e-06	1	U	K.DPLNDTVVNLLGGSK.D
874	608 – 624	880.0330	1758.0514	1757.9564	0.0951	0	75	0.00038	1	U	K.DALVSQLFVPVVAESGK.K
878	608 – 624	880.0500	1758.0854	1757.9564	0.1291	0	134	4.7e-10	1	U	K.DALVSQLFVPVVAESGK.K
881	608 – 624	880.0590	1758.1034	1757.9564	0.1471	0	87	2.4e-05	1	U	K.DALVSQLFVPVVAESGK.K
938	709 – 725	924.0630	1846.1114	1845.9625	0.1490	0	94	5.2e-06	1	U	R.YSILAPNVIPEGFVDGR.Q
939	709 – 725	616.3830	1846.1272	1845.9625	0.1647	0	74	0.00044	1	U	R.YSILAPNVIPEGFVDGR.Q
944	709 – 725	616.4070	1846.1992	1845.9625	0.2367	0	54	0.049	1	U	R.YSILAPNVIPEGFVDGR.Q
437	731 – 739	508.8080	1015.6014	1015.5549	0.0465	0	71	0.0013	1	U	K.ILEATNLDK.S
438	731 – 739	508.8140	1015.6134	1015.5549	0.0585	0	73	0.00078	1	U	K.ILEATNLDK.S

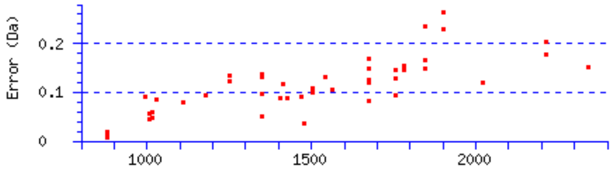

RMS error 78 ppm

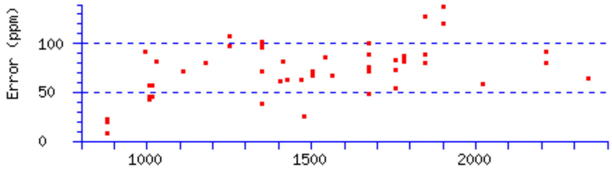

RMS error 78 ppm

```
LOCUS       OON15278                1932 aa            linear   INV 24-FEB-2017
DEFINITION  myosin head, partial [Opisthorchis viverrini].
ACCESSION   OON15278
VERSION     OON15278.1
DBLINK      BioProject: PRJNA230518
            BioSample: SAMN03378119
DBSOURCE    accession KV906128.1
KEYWORDS    .
SOURCE      Opisthorchis viverrini
  ORGANISM  Opisthorchis viverrini
            Eukaryota; Metazoa; Platyhelminthes; Trematoda; Digenea;
            Opisthorchiida; Opisthorchiata; Opisthorchiidae; Opisthorchis.
REFERENCE   1  (residues 1 to 1932)
  AUTHORS   Mitreva,M.
  TITLE     Draft genome of the nematode, Opisthorchis viverrini
  JOURNAL   Unpublished
REFERENCE   2  (residues 1 to 1932)
  AUTHORS   Mitreva,M., Pepin,K.H., Martin,J., Ozersky,P., Palsikar,V.B.,
            Zhang,X. and Wilson,R.K.
  TITLE     Direct Submission
  JOURNAL   Submitted (31-MAR-2015) The Genome Institute, Washington University
            School of Medicine, 4444 Forest Park, St. Louis, MO 63108, USA
COMMENT     Method: conceptual translation.
FEATURES             Location/Qualifiers
     source          1..1932
                     /organism="Opisthorchis viverrini"
                     /isolate="Khon Kaen"
                     /isolation_source="wild caught freshwater fish"
                     /db_xref="taxon:6198"
                     /chromosome="Unknown"
                     /sex="hermaphrodite"
                     /dev_stage="adult"
                     /lab_host="hamster"
                     /country="Thailand: Khon Kaen province"
                     /note="pooled from 10-20 individuals"
```

```
Protein         1..>1932
                /product="myosin head"
Region          28..66
                /region_name="Myosin_N"
                /note="Myosin N-terminal SH3-like domain; pfam02736"
                /db_xref="CDD:308391"
Region          93..752
                /region_name="MYSc_class_II"
                /note="class II myosins, motor domain; cd01377"
                /db_xref="CDD:276951"
Site            order(120..128,172..179,226..236,454..459)
                /site_type="other"
                /note="ATP binding site [chemical binding]"
                /db_xref="CDD:276951"
Site            120..128
                /site_type="other"
                /note="purine-binding loop"
                /db_xref="CDD:276951"
Site            172..179
                /site_type="other"
                /note="P-loop"
                /db_xref="CDD:276951"
Site            226..236
                /site_type="other"
                /note="switch I region"
                /db_xref="CDD:276951"
Site            454..459
                /site_type="other"
                /note="switch II region"
                /db_xref="CDD:276951"
Site            483..506
                /site_type="other"
                /note="relay loop"
                /db_xref="CDD:276951"
Site            684..693
                /site_type="other"
                /note="SH1 helix"
                /db_xref="CDD:276951"
Site            order(696..712,741..752)
                /site_type="other"
                /note="converter subdomain"
                /db_xref="CDD:276951"
Region          866..1911
                /region_name="SMC_N"
                /note="RecF/RecN/SMC N terminal domain; cl25732"
                /db_xref="CDD:330553"
CDS             1..1932
                /locus_tag="X801_08923"
                /coded_by="complement(join(KV906128.1:<82098..82370,
                KV906128.1:82549..82740,KV906128.1:82827..82979,
                KV906128.1:83084..83596,KV906128.1:83687..83868,
                KV906128.1:83977..84103,KV906128.1:84197..84613,
                KV906128.1:84820..85065,KV906128.1:85151..85229,
                KV906128.1:85359..85591,KV906128.1:85697..86020,
                KV906128.1:86235..86910,KV906128.1:87057..87314,
                KV906128.1:87458..87575,KV906128.1:87679..87766,
                KV906128.1:88812..88870,KV906128.1:90155..90275,
                KV906128.1:90396..90575,KV906128.1:90687..90857,
                KV906128.1:90998..91147,KV906128.1:91259..91516,
                KV906128.1:91853..91956,KV906128.1:92171..92269,
                KV906128.1:92322..92385,KV906128.1:92428..92520,
                KV906128.1:92712..92817,KV906128.1:92926..92953,
                KV906128.1:93051..93207,KV906128.1:93342..93485,
                KV906128.1:93985..94167))"
                /inference="protein motif:HMMPfam:IPR001609"
                /inference="protein motif:HMMPfam:IPR002928"
                /inference="protein motif:HMMPfam:IPR004009"
                /note="KEGG: phu:Phum_PHUM098460 0. myosin-9, putative
                K10352"
                /db_xref="InterPro:IPR001609"
                /db_xref="InterPro:IPR002928"
                /db_xref="InterPro:IPR004009"
```
