# Supplementary material for: Identification and characterization of a target antigen recognized by the monoclonal antibody against Opisthorchis viverrini
Source: PLoS One. 2025 May 29;20(5):e0324137. doi: 10.1371/journal.pone.0324137 (PMC12121735; doi:10.1371/journal.pone.0324137)
Supplement: S3 File — (PDF) [file pone.0324137.s005.pdf]

MATRIX SCIENCE Mascot Search Results

```
User          : tonkla
Email         : tonkla_ins@hotmail.com
Search title  : Partial Myosin
MS data file  : Partial Myosin_H1_01_2401.mgf
Database      : NCBIprot 20180429 (152462470 sequences; 55858910152 residues)
Taxonomy      : Other Metazoa (5474206 sequences)
Timestamp     : 13 Mar 2019 at 03:44:01 GMT
Protein hits  : OON15278.1      myosin head, partial [Opisthorchis viverrini]
                XP_009174447.1 hypothetical protein T265_09952 [Opisthorchis viverrini]
                CDS24615.1      myosin heavy chain [Echinococcus granulosus]
                BAA34954.1      myosin heavy chain [Dugesia japonica]
                AAB03660.1      myosin heavy chain [Placopecten magellanicus]
                XP_003747528.1 PREDICTED: myosin heavy chain, muscle-like [Galendromus occidentalis]
                XP_018496589.1 PREDICTED: myosin heavy chain, muscle [Galendromus occidentalis]
                XP_021957688.1 myosin heavy chain, muscle-like [Folsomia candida]
                OZC07256.1      myosin head [Onchocerca flexuosa]
                XP_014661502.1 PREDICTED: myosin heavy chain, striated muscle-like isoform X1 [Priapulus caudatus]
                KYM76081.1      Restin like protein [Atta colombica]
                XP_011669957.1 PREDICTED: uncharacterized protein LOC105440990 isoform X1 [Strongylocentrotus purpuratus]
                XP_015113809.1 PREDICTED: growth hormone-regulated TBC protein 1 [Diachasma alloeum]
                KRY15156.1      hypothetical protein T12_6453 [Trichinella patagoniensis]
                XP_012795120.1 Unconventional myosin-XVIIIa [Schistosoma haematobium]
                PSN47327.1      hypothetical protein C0J52_04406 [Blattella germanica]
```

Mascot Score Histogram

Ions score is -10*Log(P), where P is the probability that the observed match is a random event.
Individual ions scores > 54 indicate identity or extensive homology (p<0.05).
Protein scores are derived from ions scores as a non-probabilistic basis for ranking protein hits.

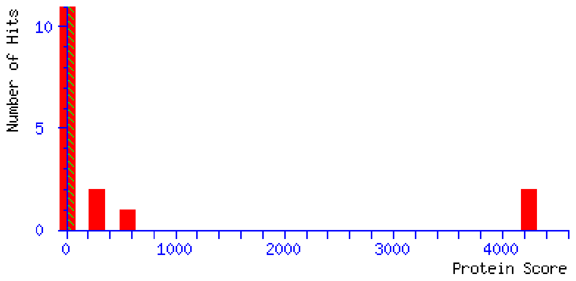

Peptide Summary Report

Format As | Peptide Summary | Help

Significance threshold p< 0.05 | Max. number of hits AUTO

Standard scoring | MudPIT scoring | Display non-significant matches | Show sub-sets 0

Show pop-ups | Suppress pop-ups | Sort unassigned Decreasing Score | Require bold red

Preferred taxonomy All entries

Select All | Select None | Search Selected | **Error tolerant**

1. OON15278.1 **Mass:** 222414 **Score:** 4244 **Matches:** 87(87) **Sequences:** 55(55) **emPAI:** 1.73
myosin head, partial [Opisthorchis viverrini]
Check to include this hit in error tolerant search

Query	Observed	Mr(expt)	Mr(calc)	Delta	Miss	Score	Expect	Rank	Unique	Peptide
261	440.7550	879.4954	879.4702	0.0252	0	60	0.016	2		K.VGTEFVTK.G
280	459.2560	916.4974	916.4614	0.0361	0	62	0.012	1		R.TALEQAER.G
292	466.2970	930.5794	930.5321	0.0474	0	56	0.043	1		R.VALTLMQR.N
297	471.8210	941.6274	941.5658	0.0617	0	71	0.001	1		K.ISNLVLQR.G
323	490.2880	978.5614	978.5109	0.0505	0	(58)	0.024	1		R.MFGWLVAR.V
345	498.3050	994.5954	994.5059	0.0896	0	60	0.016	1		R.MFGWLVAR.V + Oxidation (M)
364	508.8120	1015.6094	1015.5549	0.0545	0	(59)	0.02	1		K.ILEATNLDK.S
365	508.8160	1015.6174	1015.5549	0.0625	0	63	0.0091	1		K.ILEATNLDK.S
376	516.3540	1030.6934	1030.6175	0.0759	0	87	3.1e-05	1		K.VAFLLGVNAK.D
377	516.3660	1030.7174	1030.6175	0.0999	0	(67)	0.0035	1		K.VAFLLGVNAK.D
384	523.3180	1044.6214	1044.5451	0.0763	0	61	0.014	1		R.EVEIGGLNSK.F

☑	391	531.3120	1060.6094	1060.5335	0.0759	0	79	0.00021	1	R.AQLEMSQVR.Q
☑	392	531.3160	1060.6174	1060.5335	0.0839	0	(70)	0.0016	1	R.AQLEMSQVR.Q
☑	404	538.7990	1075.5834	1075.5080	0.0754	0	58	0.029	1	R.ANGMASQLER.K
☑	412	551.3180	1100.6214	1100.5462	0.0753	0	79	0.00018	1	R.ADQAEQALQK.L
☑	413	551.3180	1100.6214	1100.5462	0.0753	0	(57)	0.029	1	R.ADQAEQALQK.L
☑	416	551.8270	1101.6394	1101.5601	0.0794	0	77	0.00032	1	K.MQGELQQLR.S
☑	434	567.3390	1132.6634	1132.5724	0.0910	0	(56)	0.037	1	R.NLTSDLDSLR.E
☑	435	567.3490	1132.6834	1132.5724	0.1110	0	66	0.004	1	R.NLTSDLDSLR.E
☑	447	582.3390	1162.6634	1162.5652	0.0982	0	(59)	0.017	1	K.AGTLASLEDMR.D
☑	448	582.3420	1162.6694	1162.5652	0.1042	0	79	0.00017	1	K.AGTLASLEDMR.D
☑	457	590.3750	1178.7354	1178.6369	0.0985	0	57	0.03	1	K.DLMTSFLKPK.V
☑	462	594.8160	1187.6174	1187.5530	0.0644	0	66	0.0036	1	R.NSQENAELQR.Q
☑	471	601.8420	1201.6694	1201.5873	0.0821	0	(79)	0.00018	1	R.AQNEQAIAAMR.K
☑	472	601.8440	1201.6734	1201.5873	0.0861	0	90	1.4e-05	1	R.AQNEQAIAAMR.K
☑	495	625.9200	1249.8254	1249.7070	0.1184	0	68	0.0023	1	R.FPIYTAQVALK.Y
☑	506	421.5430	1261.6072	1261.6038	0.0034	0	(55)	0.047	1	K.DVEDLESSLQK.A
☑	507	631.8510	1261.6874	1261.6038	0.0837	0	(72)	0.001	1	K.DVEDLESSLQK.A
☑	508	631.8520	1261.6894	1261.6038	0.0857	0	92	9.5e-06	1	K.DVEDLESSLQK.A
☑	514	636.8820	1271.7494	1271.6469	0.1025	0	77	0.0003	1	K.QQLSAQLEEAR.H
☑	515	426.2220	1275.6442	1275.5943	0.0499	0	(55)	0.041	1	K.ATQETVDDLER.V
☑	517	638.8550	1275.6954	1275.5943	0.1012	0	(69)	0.002	1	K.ATQETVDDLER.V
☑	519	638.8600	1275.7054	1275.5943	0.1112	0	71	0.0013	1	K.ATQETVDDLER.V
☑	551	665.8660	1329.7174	1329.6161	0.1014	0	73	0.00067	1	R.EVQGQLEDEQR.Q
☑	565	449.5640	1345.6702	1345.6361	0.0341	0	(60)	0.014	1	R.ELETELEAEQR.R
☑	566	673.8680	1345.7214	1345.6361	0.0854	0	85	4.7e-05	1	R.ELETELEAEQR.R
☑	570	450.6200	1348.8382	1348.7755	0.0627	0	(64)	0.0062	1	K.VISYFAIVAAAPK.K
☑	571	675.4500	1348.8854	1348.7755	0.1100	0	(71)	0.001	1	K.VISYFAIVAAAPK.K
☑	572	675.4580	1348.9014	1348.7755	0.1260	0	100	1.6e-06	1	K.VISYFAIVAAAPK.K
☑	573	675.4650	1348.9154	1348.7755	0.1400	0	(60)	0.015	1	K.VISYFAIVAAAPK.K
☑	583	683.8880	1365.7614	1365.7252	0.0362	0	59	0.017	1	K.GLETQIQELHAK.L
☑	600	690.3670	1378.7194	1378.6220	0.0974	0	90	1.4e-05	1	R.ANMMSGEIEELR.T
☑	601	460.5850	1378.7332	1378.6220	0.1111	0	(84)	4.9e-05	1	R.ANMMSGEIEELR.T
☑	613	469.5900	1405.7482	1405.6395	0.1087	0	(63)	0.0069	1	K.IQELEAECESAK.S
☑	614	703.8820	1405.7494	1405.6395	0.1100	0	100	1.2e-06	1	K.IQELEAECESAK.S
☑	615	703.8830	1405.7514	1405.6395	0.1120	0	(84)	5.8e-05	1	K.IQELEAECESAK.S
☑	617	469.6430	1405.9072	1405.8082	0.0990	1	57	0.026	1	K.RFPIYTAQVALK.Y
☑	620	707.3770	1412.7394	1412.6098	0.1296	0	60	0.013	1	K.CTTSICNLGEMK.F
☑	633	726.3970	1450.7794	1450.6939	0.0855	1	73	0.00074	1	R.LAEKEEEFEATR.K
☑	643	736.4420	1470.8694	1470.7718	0.0976	0	91	1e-05	1	K.IAGADIEHYLLEK.S
☑	646	737.4040	1472.7934	1472.6994	0.0940	0	89	1.9e-05	1	R.IQELEEDLEAER.A
☑	647	737.4110	1472.8074	1472.6994	0.1080	0	(88)	2.3e-05	1	R.IQELEEDLEAER.A
☑	659	745.4500	1488.8854	1488.7784	0.1071	0	79	0.00019	1	R.ATELSTQAASLAAQK.R
☑	660	497.3040	1488.8902	1488.7784	0.1118	0	(68)	0.0023	1	R.ATELSTQAASLAAQK.R
☑	661	746.8870	1491.7594	1491.6875	0.0720	0	(59)	0.016	1	R.AMESQQASLEAEAK.G
☑	663	746.9020	1491.7894	1491.6875	0.1020	0	77	0.00027	1	R.AMESQQASLEAEAK.G
☑	669	501.9620	1502.8642	1502.7576	0.1065	0	(71)	0.0011	1	R.ENQSILITGESGAGK.T
☑	670	752.4400	1502.8654	1502.7576	0.1078	0	74	0.00052	1	R.ENQSILITGESGAGK.T
☑	674	503.6000	1507.7782	1507.6824	0.0958	0	(55)	0.04	1	R.AMESQQASLEAEAK.G + Oxidation (M)
☑	677	768.4070	1534.7994	1534.7297	0.0698	1	61	0.011	1	K.AGTLASLEDMRDEK.L
☑	683	771.4810	1540.9474	1540.8097	0.1378	0	94	5.9e-06	1	K.DPLNDTVVNLLGGSK.D
☑	688	779.4650	1556.9154	1556.8046	0.1109	0	58	0.02	1	K.ELEEQNVTVLQQK.N
☑	689	781.9690	1561.9234	1561.7736	0.1498	0	83	6.9e-05	1	K.FEDEQSIVAQLQR.K
☑	690	521.6520	1561.9342	1561.7736	0.1606	0	(55)	0.045	1	K.FEDEQSIVAQLQR.K
☑	701	795.4700	1588.9254	1588.7944	0.1311	0	71	0.0011	1	R.QLEEAESQLSQLSK.V
☑	711	810.9440	1619.8734	1619.7638	0.1096	0	80	0.00013	1	K.LDETTNQLSEQASGK.A
☑	721	814.4930	1626.9714	1626.8464	0.1250	0	(72)	0.00089	1	R.QVEEAEEIAAINLAK.Y
☑	722	543.3350	1626.9832	1626.8464	0.1368	0	86	3.6e-05	1	R.QVEEAEEIAAINLAK.Y
☑	726	823.4670	1644.9194	1644.8029	0.1166	0	111	9.7e-08	1	R.LQGELEDLMVDVER.A
☑	729	825.4450	1648.8754	1648.7614	0.1141	0	111	9.7e-08	1	K.LESTLDEMEENLAR.E
☑	731	550.6460	1648.9162	1648.7614	0.1548	0	(66)	0.0033	1	K.LESTLDEMEENLAR.E
☑	732	550.6520	1648.9342	1648.7614	0.1728	0	(54)	0.047	1	K.LESTLDEMEENLAR.E
☑	737	835.9700	1669.9254	1669.8159	0.1096	0	96	3.4e-06	1	K.AEVDDLHSQLESLSK.A
☑	741	839.0170	1676.0194	1675.8893	0.1301	0	104	4.6e-07	1	K.GQNLNQVTYAVSALAK.S
☑	742	559.6830	1676.0272	1675.8893	0.1379	0	(69)	0.0017	1	K.GQNLNQVTYAVSALAK.S
☑	749	846.9280	1691.8414	1691.7308	0.1107	0	101	9.9e-07	1	R.LADEEDANSNLNEMK.K
☑	754	568.0030	1700.9872	1700.8581	0.1291	0	(76)	0.0003	1	R.TLQGEIAQQDEQITK.L
☑	755	851.5070	1700.9994	1700.8581	0.1414	0	92	7.6e-06	1	R.TLQGEIAQQDEQITK.L
☑	772	880.0420	1758.0694	1757.9564	0.1131	0	111	1e-07	1	K.DALVSQLFVPVVAESGK.K
☑	787	595.3690	1783.0852	1782.9476	0.1376	1	(78)	0.00027	1	K.NKDPLNDTVVNLLGGSK.D
☑	788	892.5520	1783.0894	1782.9476	0.1419	1	82	0.0001	1	K.NKDPLNDTVVNLLGGSK.D

☑	795	894.0030	1785.9914	1785.8745	0.1170	0	(69)	0.0014	1		R.LEEQDGVNAQQVDLTK.K
☑	796	596.3470	1786.0192	1785.8745	0.1447	0	80	0.00012	1		R.LEEQDGVNAQQVDLTK.K
☑	817	924.0620	1846.1094	1845.9625	0.1470	0	68	0.0019	1		R.YSILAPNVIPEGFVDGR.Q
☑	827	618.3660	1852.0762	1851.8962	0.1799	0	56	0.033	1	U	R.QQASHVEAELEEVVER.L
☑	871	739.4390	2215.2952	2215.1120	0.1831	0	75	0.00037	1		K.GSLEDQIVQANPVLEAYGNAK.T
☑	878	782.1400	2343.3982	2343.2070	0.1912	1	71	0.00071	1		K.KGSLEDQIVQANPVLEAYGNAK.T

---

2. XP_009174447.1 **Mass:** 222567 **Score:** 4159 **Matches:** 85(85) **Sequences:** 54(54) **emPAI:** 1.68
hypothetical protein T265_09952 [Opisthorchis viverrini]

☐ Check to include this hit in error tolerant search

	Query	Observed	Mr(expt)	Mr(calc)	Delta	Miss	Score	Expect	Rank	Unique	Peptide
	261	440.7550	879.4954	879.4702	0.0252	0	60	0.016	2		K.VGTEFVTK.G
	280	459.2560	916.4974	916.4614	0.0361	0	62	0.012	1		R.TALEQAER.G
	292	466.2970	930.5794	930.5321	0.0474	0	56	0.043	1		R.VALTLMQR.N
	297	471.8210	941.6274	941.5658	0.0617	0	71	0.001	1		K.ISNLVLQR.G
	323	490.2880	978.5614	978.5109	0.0505	0	(58)	0.024	1		R.MFGWLVAR.V
	345	498.3050	994.5954	994.5059	0.0896	0	60	0.016	1		R.MFGWLVAR.V + Oxidation (M)
	364	508.8120	1015.6094	1015.5549	0.0545	0	(59)	0.02	1		K.ILEATNLDK.S
	365	508.8160	1015.6174	1015.5549	0.0625	0	63	0.0091	1		K.ILEATNLDK.S
	376	516.3540	1030.6934	1030.6175	0.0759	0	87	3.1e-05	1		K.VAFLLGVNAK.D
	377	516.3660	1030.7174	1030.6175	0.0999	0	(67)	0.0035	1		K.VAFLLGVNAK.D
	384	523.3180	1044.6214	1044.5451	0.0763	0	61	0.014	1		R.EVEIGGLNSK.F
	391	531.3120	1060.6094	1060.5335	0.0759	0	79	0.00021	1		R.AQLEMSQVR.Q
	392	531.3160	1060.6174	1060.5335	0.0839	0	(70)	0.0016	1		R.AQLEMSQVR.Q
	404	538.7990	1075.5834	1075.5080	0.0754	0	58	0.029	1		R.ANGMASQLER.K
	412	551.3180	1100.6214	1100.5462	0.0753	0	79	0.00018	1		R.ADQAEQALQK.L
	413	551.3180	1100.6214	1100.5462	0.0753	0	(57)	0.029	1		R.ADQAEQALQK.L
	416	551.8270	1101.6394	1101.5601	0.0794	0	77	0.00032	1		K.MQGELQQLR.S
	434	567.3390	1132.6634	1132.5724	0.0910	0	(56)	0.037	1		R.NLTSDLDSLR.E
	435	567.3490	1132.6834	1132.5724	0.1110	0	66	0.004	1		R.NLTSDLDSLR.E
	447	582.3390	1162.6634	1162.5652	0.0982	0	(59)	0.017	1		K.AGTLASLEDMR.D
	448	582.3420	1162.6694	1162.5652	0.1042	0	79	0.00017	1		K.AGTLASLEDMR.D
	457	590.3750	1178.7354	1178.6369	0.0985	0	57	0.03	1		K.DLMTSFLKPK.V
	462	594.8160	1187.6174	1187.5530	0.0644	0	66	0.0036	1		R.NSQENAELQR.Q
☑	467	598.3490	1194.6834	1194.6105	0.0729	0	62	0.0099	1	U	R.GVSAAPAGAGPANR.A
	471	601.8420	1201.6694	1201.5873	0.0821	0	(79)	0.00018	1		R.AQNEQAIAAMR.K
	472	601.8440	1201.6734	1201.5873	0.0861	0	90	1.4e-05	1		R.AQNEQAIAAMR.K
	495	625.9200	1249.8254	1249.7070	0.1184	0	68	0.0023	1		R.FPIYTAQVALK.Y
	506	421.5430	1261.6072	1261.6038	0.0034	0	(55)	0.047	1		K.DVEDLESSLQK.A
	507	631.8510	1261.6874	1261.6038	0.0837	0	(72)	0.001	1		K.DVEDLESSLQK.A
	508	631.8520	1261.6894	1261.6038	0.0857	0	92	9.5e-06	1		K.DVEDLESSLQK.A
	514	636.8820	1271.7494	1271.6469	0.1025	0	77	0.0003	1		K.QQLSAQLEEAR.H
	515	426.2220	1275.6442	1275.5943	0.0499	0	(55)	0.041	1		K.ATQETVDDLER.V
	517	638.8550	1275.6954	1275.5943	0.1012	0	(69)	0.002	1		K.ATQETVDDLER.V
	519	638.8600	1275.7054	1275.5943	0.1112	0	71	0.0013	1		K.ATQETVDDLER.V
	551	665.8660	1329.7174	1329.6161	0.1014	0	73	0.00067	1		R.EVQGQLEDEQR.Q
	565	449.5640	1345.6702	1345.6361	0.0341	0	(60)	0.014	1		R.ELETELEAEQR.R
	566	673.8680	1345.7214	1345.6361	0.0854	0	85	4.7e-05	1		R.ELETELEAEQR.R
	570	450.6200	1348.8382	1348.7755	0.0627	0	(64)	0.0062	1		K.VISYFAIVAAAPK.K
	571	675.4500	1348.8854	1348.7755	0.1100	0	(71)	0.001	1		K.VISYFAIVAAAPK.K
	572	675.4580	1348.9014	1348.7755	0.1260	0	100	1.6e-06	1		K.VISYFAIVAAAPK.K
	573	675.4650	1348.9154	1348.7755	0.1400	0	(60)	0.015	1		K.VISYFAIVAAAPK.K
	583	683.8880	1365.7614	1365.7252	0.0362	0	59	0.017	1		K.GLETQIQELHAK.L
	600	690.3670	1378.7194	1378.6220	0.0974	0	90	1.4e-05	1		R.ANMMSGEIEELR.T
	601	460.5850	1378.7332	1378.6220	0.1111	0	(84)	4.9e-05	1		R.ANMMSGEIEELR.T
	613	469.5900	1405.7482	1405.6395	0.1087	0	(63)	0.0069	1		K.IQELEAECESAK.S
	614	703.8820	1405.7494	1405.6395	0.1100	0	100	1.2e-06	1		K.IQELEAECESAK.S
	615	703.8830	1405.7514	1405.6395	0.1120	0	(84)	5.8e-05	1		K.IQELEAECESAK.S
	617	469.6430	1405.9072	1405.8082	0.0990	1	57	0.026	1		K.RFPIYTAQVALK.Y
	620	707.3770	1412.7394	1412.6098	0.1296	0	60	0.013	1		K.CTTSICNLGEMK.F
	633	726.3970	1450.7794	1450.6939	0.0855	1	73	0.00074	1		R.LAEKEEEFEATR.K
	643	736.4420	1470.8694	1470.7718	0.0976	0	91	1e-05	1		K.IAGADIEHYLLEK.S
	659	745.4500	1488.8854	1488.7784	0.1071	0	79	0.00019	1		R.ATELSTQAASLAAQK.R
	660	497.3040	1488.8902	1488.7784	0.1118	0	(68)	0.0023	1		R.ATELSTQAASLAAQK.R
	661	746.8870	1491.7594	1491.6875	0.0720	0	(59)	0.016	1		R.AMESQQASLEAEAK.G
	663	746.9020	1491.7894	1491.6875	0.1020	0	77	0.00027	1		R.AMESQQASLEAEAK.G
	669	501.9620	1502.8642	1502.7576	0.1065	0	(71)	0.0011	1		R.ENQSILITGESGAGK.T
	670	752.4400	1502.8654	1502.7576	0.1078	0	74	0.00052	1		R.ENQSILITGESGAGK.T
	674	503.6000	1507.7782	1507.6824	0.0958	0	(55)	0.04	1		R.AMESQQASLEAEAK.G + Oxidation (M)
	677	768.4070	1534.7994	1534.7297	0.0698	1	61	0.011	1		K.AGTLASLEDMRDEK.L
	683	771.4810	1540.9474	1540.8097	0.1378	0	94	5.9e-06	1		K.DPLNDTVVNLLGGSK.D

688	779.4650	1556.9154	1556.8046	0.1109	0	58	0.02	1		K.ELEEQNVTVLQQK.N
689	781.9690	1561.9234	1561.7736	0.1498	0	83	6.9e-05	1		K.FEDEQSIVAQLQR.K
690	521.6520	1561.9342	1561.7736	0.1606	0	(55)	0.045	1		K.FEDEQSIVAQLQR.K
701	795.4700	1588.9254	1588.7944	0.1311	0	71	0.0011	1		R.QLEEAESQLSQLSK.V
711	810.9440	1619.8734	1619.7638	0.1096	0	80	0.00013	1		K.LDETTNQLSEQASGK.A
721	814.4930	1626.9714	1626.8464	0.1250	0	(72)	0.00089	1		R.QVEEAEEIAAINLAK.Y
722	543.3350	1626.9832	1626.8464	0.1368	0	86	3.6e-05	1		R.QVEEAEEIAAINLAK.Y
726	823.4670	1644.9194	1644.8029	0.1166	0	111	9.7e-08	1		R.LQGELEDLMVDVER.A
729	825.4450	1648.8754	1648.7614	0.1141	0	111	9.7e-08	1		K.LESTLDEMEENLAR.E
731	550.6460	1648.9162	1648.7614	0.1548	0	(66)	0.0033	1		K.LESTLDEMEENLAR.E
732	550.6520	1648.9342	1648.7614	0.1728	0	(54)	0.047	1		K.LESTLDEMEENLAR.E
737	835.9700	1669.9254	1669.8159	0.1096	0	96	3.4e-06	1		K.AEVDDLHSQLESLSK.A
741	839.0170	1676.0194	1675.8893	0.1301	0	104	4.6e-07	1		K.GQNLNQVTYAVSALAK.S
742	559.6830	1676.0272	1675.8893	0.1379	0	(69)	0.0017	1		K.GQNLNQVTYAVSALAK.S
749	846.9280	1691.8414	1691.7308	0.1107	0	101	9.9e-07	1		R.LADEEDANSNLNEMK.K
754	568.0030	1700.9872	1700.8581	0.1291	0	(76)	0.0003	1		R.TLQGEIAQQDEQITK.L
755	851.5070	1700.9994	1700.8581	0.1414	0	92	7.6e-06	1		R.TLQGEIAQQDEQITK.L
772	880.0420	1758.0694	1757.9564	0.1131	0	111	1e-07	1		K.DALVSQLFVPVVAESGK.K
787	595.3690	1783.0852	1782.9476	0.1376	1	(78)	0.00027	1		K.NKDPLNDTVVNLLGGSK.D
788	892.5520	1783.0894	1782.9476	0.1419	1	82	0.0001	1		K.NKDPLNDTVVNLLGGSK.D
795	894.0030	1785.9914	1785.8745	0.1170	0	(69)	0.0014	1		R.LEEQDGVNAQQVDLTK.K
796	596.3470	1786.0192	1785.8745	0.1447	0	80	0.00012	1		R.LEEQDGVNAQQVDLTK.K
817	924.0620	1846.1094	1845.9625	0.1470	0	68	0.0019	1		R.YSILAPNVIPEGFVDGR.Q
871	739.4390	2215.2952	2215.1120	0.1831	0	75	0.00037	1		K.GSLEDQIVQANPVLEAYGNAK.T
878	782.1400	2343.3982	2343.2070	0.1912	1	71	0.00071	1		K.KGSLEDQIVQANPVLEAYGNAK.T

---

3. CDS24615.1 **Mass:** 224152 **Score:** 595 **Matches:** 13(13) **Sequences:** 7(7) **emPAI:** 0.13
myosin heavy chain [Echinococcus granulosus]
☐ Check to include this hit in error tolerant search

	Query	Observed	Mr(expt)	Mr(calc)	Delta	Miss	Score	Expect	Rank	Unique	Peptide
	376	516.3540	1030.6934	1030.6175	0.0759	0	87	3.1e-05	1		K.VAFLLGVNAK.D
	377	516.3660	1030.7174	1030.6175	0.0999	0	(67)	0.0035	1		K.VAFLLGVNAK.D
	412	551.3180	1100.6214	1100.5462	0.0753	0	79	0.00018	1		R.ADQAEQALQK.L
	413	551.3180	1100.6214	1100.5462	0.0753	0	(57)	0.029	1		R.ADQAEQALQK.L
☑	512	**424.8980**	**1271.6722**	**1271.6469**	**0.0252**	**0**	**61**	**0.012**	**1**	**U**	**K.QQLASQLEEAR.R**
	514	636.8820	1271.7494	1271.6469	0.1025	0	(59)	0.017	2	U	K.QQLASQLEEAR.R
	633	726.3970	1450.7794	1450.6939	0.0855	1	73	0.00074	1		R.LAEKEEEFEATR.K
	669	501.9620	1502.8642	1502.7576	0.1065	0	(71)	0.0011	1		R.ENQSILITGESGAGK.T
	670	752.4400	1502.8654	1502.7576	0.1078	0	74	0.00052	1		R.ENQSILITGESGAGK.T
	726	823.4670	1644.9194	1644.8029	0.1166	0	111	9.7e-08	1	U	R.LQGEIEDLMVDVER.A
	729	825.4450	1648.8754	1648.7614	0.1141	0	111	9.7e-08	1		K.LESTLDEMEENLAR.E
	731	550.6460	1648.9162	1648.7614	0.1548	0	(66)	0.0033	1		K.LESTLDEMEENLAR.E
	732	550.6520	1648.9342	1648.7614	0.1728	0	(54)	0.047	1		K.LESTLDEMEENLAR.E

Proteins matching the same set of peptides:
CDS41550.1 **Mass:** 224160 **Score:** 595 **Matches:** 13(13) **Sequences:** 7(7)
myosin heavy chain [Echinococcus multilocularis]
XP_024352951.1 **Mass:** 227397 **Score:** 595 **Matches:** 13(13) **Sequences:** 7(7)
Myosin heavy chain, striated muscle [Echinococcus granulosus]

---

4. BAA34954.1 **Mass:** 225289 **Score:** 328 **Matches:** 7(7) **Sequences:** 4(4) **emPAI:** 0.07
myosin heavy chain [Dugesia japonica]
☐ Check to include this hit in error tolerant search

Query	Observed	Mr(expt)	Mr(calc)	Delta	Miss	Score	Expect	Rank	Unique	Peptide
376	516.3540	1030.6934	1030.6175	0.0759	0	87	3.1e-05	1		K.VAFLLGVNAK.D
377	516.3660	1030.7174	1030.6175	0.0999	0	(67)	0.0035	1		K.VAFLLGVNAK.D
412	551.3180	1100.6214	1100.5462	0.0753	0	79	0.00018	1		R.ADQAEQALQK.L
413	551.3180	1100.6214	1100.5462	0.0753	0	(57)	0.029	1		R.ADQAEQALQK.L
633	726.3970	1450.7794	1450.6939	0.0855	1	73	0.00074	1	U	R.IAEKEEEFEATR.V
646	737.4040	1472.7934	1472.6994	0.0940	0	89	1.9e-05	1		R.IQELEEDLEAER.Q
647	737.4110	1472.8074	1472.6994	0.1080	0	(88)	2.3e-05	1		R.IQELEEDLEAER.Q

---

5. AAB03660.1 **Mass:** 224190 **Score:** 147 **Matches:** 3(3) **Sequences:** 2(2) **emPAI:** 0.04
myosin heavy chain [Placopecten magellanicus]
☐ Check to include this hit in error tolerant search

	Query	Observed	Mr(expt)	Mr(calc)	Delta	Miss	Score	Expect	Rank	Unique	Peptide
☑	261	440.7550	879.4954	879.4372	0.0583	0	61	0.01	1		K.VGTEMVTK.G + Oxidation (M)

721	814.4930	1626.9714	1626.8464	0.1250	0	(72)	0.00089	1		R.QVEEAEEIAAINLAK.Y
722	543.3350	1626.9832	1626.8464	0.1368	0	86	3.6e-05	1		R.QVEEAEEIAAINLAK.Y

Proteins matching the same set of peptides:

AAB03661.1 **Mass:** 225130 **Score:** 147 **Matches:** 3(3) **Sequences:** 2(2)
myosin heavy chain [Placopecten magellanicus]
BAB40711.1 **Mass:** 224174 **Score:** 147 **Matches:** 3(3) **Sequences:** 2(2)
myosin [Mizuhopecten yessoensis]
OWF43611.1 **Mass:** 197791 **Score:** 147 **Matches:** 3(3) **Sequences:** 2(2)
Myosin heavy chain, striated muscle [Mizuhopecten yessoensis]
XP_021367464.1 **Mass:** 226207 **Score:** 147 **Matches:** 3(3) **Sequences:** 2(2)
myosin heavy chain, striated muscle isoform X1 [Mizuhopecten yessoensis]
XP_021367465.1 **Mass:** 226279 **Score:** 147 **Matches:** 3(3) **Sequences:** 2(2)
myosin heavy chain, striated muscle isoform X2 [Mizuhopecten yessoensis]
XP_021367466.1 **Mass:** 226147 **Score:** 147 **Matches:** 3(3) **Sequences:** 2(2)
myosin heavy chain, striated muscle isoform X3 [Mizuhopecten yessoensis]
XP_021367467.1 **Mass:** 226219 **Score:** 147 **Matches:** 3(3) **Sequences:** 2(2)
myosin heavy chain, striated muscle isoform X4 [Mizuhopecten yessoensis]
XP_021367468.1 **Mass:** 226203 **Score:** 147 **Matches:** 3(3) **Sequences:** 2(2)
myosin heavy chain, striated muscle isoform X5 [Mizuhopecten yessoensis]
XP_021367469.1 **Mass:** 226143 **Score:** 147 **Matches:** 3(3) **Sequences:** 2(2)
myosin heavy chain, striated muscle isoform X6 [Mizuhopecten yessoensis]
XP_021367470.1 **Mass:** 226114 **Score:** 147 **Matches:** 3(3) **Sequences:** 2(2)
myosin heavy chain, striated muscle isoform X7 [Mizuhopecten yessoensis]
XP_021367471.1 **Mass:** 224794 **Score:** 147 **Matches:** 3(3) **Sequences:** 2(2)
myosin heavy chain, striated muscle isoform X8 [Mizuhopecten yessoensis]
XP_021367472.1 **Mass:** 224734 **Score:** 147 **Matches:** 3(3) **Sequences:** 2(2)
myosin heavy chain, striated muscle isoform X9 [Mizuhopecten yessoensis]
XP_021367473.1 **Mass:** 223985 **Score:** 147 **Matches:** 3(3) **Sequences:** 2(2)
myosin heavy chain, striated muscle isoform X10 [Mizuhopecten yessoensis]
XP_021367474.1 **Mass:** 224641 **Score:** 147 **Matches:** 3(3) **Sequences:** 2(2)
myosin heavy chain, striated muscle isoform X11 [Mizuhopecten yessoensis]
XP_021367475.1 **Mass:** 226275 **Score:** 147 **Matches:** 3(3) **Sequences:** 2(2)
myosin heavy chain, striated muscle isoform X12 [Mizuhopecten yessoensis]
P24733.1 **Mass:** 223824 **Score:** 145 **Matches:** 3(3) **Sequences:** 2(2)
RecName: Full=Myosin heavy chain, striated muscle
AAC46490.1 **Mass:** 225198 **Score:** 145 **Matches:** 3(3) **Sequences:** 2(2)
myosin heavy chain [Argopecten irradians]
AAD52842.1 **Mass:** 224145 **Score:** 145 **Matches:** 3(3) **Sequences:** 2(2)
myosin heavy chain [Pecten maximus]

---

6. XP_003747528.1 **Mass:** 222499 **Score:** 132 **Matches:** 2(2) **Sequences:** 2(2) **emPAI:** 0.04
PREDICTED: myosin heavy chain, muscle-like [Galendromus occidentalis]
☐ Check to include this hit in error tolerant search

Query	Observed	Mr(expt)	Mr(calc)	Delta	Miss	Score	Expect	Rank	Unique	Peptide
261	440.7550	879.4954	879.4372	0.0583	0	61	0.01	1		R.VGTEMVTK.G + Oxidation (M)
701	795.4700	1588.9254	1588.7944	0.1311	0	71	0.0011	1		R.QLEEAESQISQLSK.Q

Proteins matching the same set of peptides:

XP_022711325.1 **Mass:** 183245 **Score:** 132 **Matches:** 2(2) **Sequences:** 2(2)
myosin heavy chain, muscle-like isoform X1 [Varroa jacobsoni]
XP_022711327.1 **Mass:** 182888 **Score:** 132 **Matches:** 2(2) **Sequences:** 2(2)
myosin heavy chain, muscle-like isoform X2 [Varroa jacobsoni]
XP_022709639.1 **Mass:** 444999 **Score:** 131 **Matches:** 2(2) **Sequences:** 2(2)
uncharacterized protein LOC111272448 [Varroa jacobsoni]
XP_022647816.1 **Mass:** 672553 **Score:** 127 **Matches:** 2(2) **Sequences:** 2(2)
LOW QUALITY PROTEIN: uncharacterized protein LOC111244705 [Varroa destructor]

---

7. XP_018496589.1 **Mass:** 223260 **Score:** 130 **Matches:** 2(2) **Sequences:** 2(2) **emPAI:** 0.04
PREDICTED: myosin heavy chain, muscle [Galendromus occidentalis]
☐ Check to include this hit in error tolerant search

Query	Observed	Mr(expt)	Mr(calc)	Delta	Miss	Score	Expect	Rank	Unique	Peptide
261	440.7550	879.4954	879.4702	0.0252	0	60	0.016	2		K.VGTEFVTK.G
701	795.4700	1588.9254	1588.7944	0.1311	0	71	0.0011	1		R.QLEEAESQISQLSK.Q

Proteins matching the same set of peptides:

OQR72717.1 **Mass:** 222906 **Score:** 129 **Matches:** 2(2) **Sequences:** 2(2)
myosin heavy chain [Tropilaelaps mercedesae]

8. XP_021957688.1 **Mass:** 223026 **Score:** 129 **Matches:** 2(2) **Sequences:** 2(2) **emPAI:** 0.04
myosin heavy chain, muscle-like [Folsomia candida]
Check to include this hit in error tolerant search

Query	Observed	Mr(expt)	Mr(calc)	Delta	Miss	Score	Expect	Rank	Unique	Peptide
261	440.7550	879.4954	879.4702	0.0252	0	60	0.016	2		K.VGTEFVTK.G
722	543.3350	1626.9832	1626.8464	0.1368	0	71	0.0011	2	U	R.QIEEAEEVAALNLAK.F

9. OZC07256.1 **Mass:** 63064 **Score:** 91 **Matches:** 1(1) **Sequences:** 1(1) **emPAI:** 0.06
myosin head [Onchocerca flexuosa]
Check to include this hit in error tolerant search

Query	Observed	Mr(expt)	Mr(calc)	Delta	Miss	Score	Expect	Rank	Unique	Peptide
643	736.4420	1470.8694	1470.7718	0.0976	0	91	1e-05	1	U	R.LAGADIEHYLLEK.S

Proteins matching the same set of peptides:
KRZ15244.1 **Mass:** 390032 **Score:** 91 **Matches:** 1(1) **Sequences:** 1(1)
Myosin-4 [Trichinella zimbabwensis]
KRZ15245.1 **Mass:** 387501 **Score:** 91 **Matches:** 1(1) **Sequences:** 1(1)
Myosin-4 [Trichinella zimbabwensis]
KRZ15246.1 **Mass:** 388858 **Score:** 91 **Matches:** 1(1) **Sequences:** 1(1)
Myosin-4 [Trichinella zimbabwensis]
KRZ15247.1 **Mass:** 365778 **Score:** 91 **Matches:** 1(1) **Sequences:** 1(1)
Myosin-4 [Trichinella zimbabwensis]
KRY21280.1 **Mass:** 330722 **Score:** 91 **Matches:** 1(1) **Sequences:** 1(1)
Myosin-3, partial [Trichinella patagoniensis]
KRY21281.1 **Mass:** 389422 **Score:** 91 **Matches:** 1(1) **Sequences:** 1(1)
Myosin-3 [Trichinella patagoniensis]
KRY21283.1 **Mass:** 386223 **Score:** 91 **Matches:** 1(1) **Sequences:** 1(1)
Myosin-3 [Trichinella patagoniensis]
KRX23706.1 **Mass:** 226879 **Score:** 91 **Matches:** 1(1) **Sequences:** 1(1)
Myosin-3 [Trichinella nelsoni]
KRX23707.1 **Mass:** 229355 **Score:** 91 **Matches:** 1(1) **Sequences:** 1(1)
Myosin-3 [Trichinella nelsoni]
KRX23708.1 **Mass:** 225530 **Score:** 91 **Matches:** 1(1) **Sequences:** 1(1)
Myosin-3 [Trichinella nelsoni]
CRZ24364.1 **Mass:** 233347 **Score:** 91 **Matches:** 1(1) **Sequences:** 1(1)
BMA-MYO-5 [Brugia malayi]
KRZ28538.1 **Mass:** 226621 **Score:** 91 **Matches:** 1(1) **Sequences:** 1(1)
Myosin-3 [Trichinella pseudospiralis]
KRZ28539.1 **Mass:** 228646 **Score:** 91 **Matches:** 1(1) **Sequences:** 1(1)
Myosin-3 [Trichinella pseudospiralis]
KRY32311.1 **Mass:** 225793 **Score:** 91 **Matches:** 1(1) **Sequences:** 1(1)
Myosin-3 [Trichinella spiralis]
KRY32312.1 **Mass:** 232393 **Score:** 91 **Matches:** 1(1) **Sequences:** 1(1)
Myosin-3 [Trichinella spiralis]
KRZ35309.1 **Mass:** 378761 **Score:** 91 **Matches:** 1(1) **Sequences:** 1(1)
Myosin-3 [Trichinella pseudospiralis]
OUC43934.1 **Mass:** 217093 **Score:** 91 **Matches:** 1(1) **Sequences:** 1(1)
myosin head [Trichinella nativa]
KRX45305.1 **Mass:** 226554 **Score:** 91 **Matches:** 1(1) **Sequences:** 1(1)
Myosin-4 [Trichinella murrelli]
KRX45306.1 **Mass:** 225726 **Score:** 91 **Matches:** 1(1) **Sequences:** 1(1)
Myosin-3 [Trichinella murrelli]
KRY52562.1 **Mass:** 375938 **Score:** 91 **Matches:** 1(1) **Sequences:** 1(1)
Myosin-3 [Trichinella britovi]
KRX65426.1 **Mass:** 403437 **Score:** 91 **Matches:** 1(1) **Sequences:** 1(1)
Myosin-3 [Trichinella sp. T9]
KRX71365.1 **Mass:** 208074 **Score:** 91 **Matches:** 1(1) **Sequences:** 1(1)
Myosin-4 [Trichinella sp. T6]
PDM75142.1 **Mass:** 218873 **Score:** 91 **Matches:** 1(1) **Sequences:** 1(1)
myo-5 [Pristionchus pacificus]
KRY75675.1 **Mass:** 228631 **Score:** 91 **Matches:** 1(1) **Sequences:** 1(1)
Myosin-3, partial [Trichinella pseudospiralis]
KRY75676.1 **Mass:** 226606 **Score:** 91 **Matches:** 1(1) **Sequences:** 1(1)
Myosin-3, partial [Trichinella pseudospiralis]
KRZ76776.1 **Mass:** 226971 **Score:** 91 **Matches:** 1(1) **Sequences:** 1(1)
Myosin-3, partial [Trichinella papuae]

KHN80466.1 **Mass:** 228046 **Score:** 91 **Matches:** 1(1) **Sequences:** 1(1)
Myosin-4 [Toxocara canis]
EJW82348.1 **Mass:** 54800 **Score:** 91 **Matches:** 1(1) **Sequences:** 1(1)
hypothetical protein WUBG_06741 [Wuchereria bancrofti]
KRY86892.1 **Mass:** 228749 **Score:** 91 **Matches:** 1(1) **Sequences:** 1(1)
Myosin-3 [Trichinella pseudospiralis]
KRY86893.1 **Mass:** 225766 **Score:** 91 **Matches:** 1(1) **Sequences:** 1(1)
Myosin-3 [Trichinella pseudospiralis]
KRY86894.1 **Mass:** 226725 **Score:** 91 **Matches:** 1(1) **Sequences:** 1(1)
Myosin-3 [Trichinella pseudospiralis]
KRZ97189.1 **Mass:** 229251 **Score:** 91 **Matches:** 1(1) **Sequences:** 1(1)
Myosin-3 [Trichinella sp. T8]
XP_001901755.1 **Mass:** 232778 **Score:** 91 **Matches:** 1(1) **Sequences:** 1(1)
Myosin tail family protein [Brugia malayi]
XP_020307061.1 **Mass:** 229477 **Score:** 91 **Matches:** 1(1) **Sequences:** 1(1)
myosin tail family protein [Loa loa]
XP_024508381.1 **Mass:** 229403 **Score:** 89 **Matches:** 1(1) **Sequences:** 1(1)
Myosin heavy chain [Strongyloides ratti]

---

10. XP_014661502.1 **Mass:** 225375 **Score:** 74 **Matches:** 2(2) **Sequences:** 1(1) **emPAI:** 0.02
PREDICTED: myosin heavy chain, striated muscle-like isoform X1 [Priapulus caudatus]
☐ Check to include this hit in error tolerant search

Query	Observed	Mr(expt)	Mr(calc)	Delta	Miss	Score	Expect	Rank	Unique	Peptide
669	501.9620	1502.8642	1502.7576	0.1065	0	(71)	0.0011	1	U	R.ENQSLLITGESGAGK.T
670	752.4400	1502.8654	1502.7576	0.1078	0	74	0.00052	1	U	R.ENQSLLITGESGAGK.T

---

11. KYM76081.1 **Score:** 64 **Matches:** 2(2) **Sequences:** 1(1) **emPAI:** 0.02
Restin like protein [Atta colombica]
☐ Check to include this hit in error tolerant search

Query	Observed	Mr(expt)	Mr(calc)	Delta	Miss	Score	Expect	Rank	Unique	Peptide
646	737.4040	1472.7934	1472.7722	0.0212	1	(58)	0.023	2	U	R.IKELEDELTVER.R
647	737.4110	1472.8074	1472.7722	0.0352	1	64	0.0061	2	U	R.IKELEDELTVER.R

Proteins matching the same set of peptides:
XP_012056043.1 **Score:** 64 **Matches:** 2(2) **Sequences:** 1(1)
PREDICTED: LOW QUALITY PROTEIN: restin homolog [Atta cephalotes]
XP_018057394.1 **Score:** 64 **Matches:** 2(2) **Sequences:** 1(1)
PREDICTED: restin homolog isoform X1 [Atta colombica]
XP_018057396.1 **Score:** 64 **Matches:** 2(2) **Sequences:** 1(1)
PREDICTED: restin homolog isoform X2 [Atta colombica]
XP_018057397.1 **Score:** 64 **Matches:** 2(2) **Sequences:** 1(1)
PREDICTED: restin homolog isoform X3 [Atta colombica]
XP_018057398.1 **Score:** 64 **Matches:** 2(2) **Sequences:** 1(1)
PREDICTED: restin homolog isoform X4 [Atta colombica]
XP_018057399.1 **Score:** 64 **Matches:** 2(2) **Sequences:** 1(1)
PREDICTED: restin homolog isoform X5 [Atta colombica]
XP_018057401.1 **Score:** 64 **Matches:** 2(2) **Sequences:** 1(1)
PREDICTED: restin homolog isoform X6 [Atta colombica]

---

12. XP_011669957.1 **Mass:** 13989 **Score:** 62 **Matches:** 1(1) **Sequences:** 1(1) **emPAI:** 0.30
PREDICTED: uncharacterized protein LOC105440990 isoform X1 [Strongylocentrotus purpuratus]
☐ Check to include this hit in error tolerant search

Query	Observed	Mr(expt)	Mr(calc)	Delta	Miss	Score	Expect	Rank	Unique	Peptide
280	459.2560	916.4974	916.4978	-0.0003	1	62	0.012	1	U	R.TALEKAER.L

---

13. XP_015113809.1 **Mass:** 41014 **Score:** 56 **Matches:** 1(1) **Sequences:** 1(1) **emPAI:** 0.10
PREDICTED: growth hormone-regulated TBC protein 1 [Diachasma alloeum]
☐ Check to include this hit in error tolerant search

Query	Observed	Mr(expt)	Mr(calc)	Delta	Miss	Score	Expect	Rank	Unique	Peptide
292	466.2970	930.5794	930.5684	0.0110	1	56	0.043	1	U	R.VALTLMKR.N

---

14. KRY15156.1 **Score:** 56 **Matches:** 1(1) **Sequences:** 1(1) **emPAI:** 0.11
hypothetical protein T12_6453 [Trichinella patagoniensis]
☐ Check to include this hit in error tolerant search

Query	Observed	Mr(expt)	Mr(calc)	Delta	Miss	Score	Expect	Rank	Unique	Peptide
614	703.8820	1405.7494	1405.7122	0.0372	1	56	0.036	2	U	K.IKELEADNSMLK.M + Oxidation (M)

Proteins matching the same set of peptides:
OUC47575.1 **Score:** 56 **Matches:** 1(1) **Sequences:** 1(1)
hypothetical protein D917_06838 [Trichinella nativa]
KRX48584.1 **Score:** 56 **Matches:** 1(1) **Sequences:** 1(1)
hypothetical protein T05_11448 [Trichinella murrelli]
KRY50878.1 **Score:** 56 **Matches:** 1(1) **Sequences:** 1(1)
hypothetical protein T03_5766 [Trichinella britovi]
KRX55741.1 **Score:** 56 **Matches:** 1(1) **Sequences:** 1(1)
hypothetical protein T09_6222 [Trichinella sp. T9]
KRX75988.1 **Score:** 56 **Matches:** 1(1) **Sequences:** 1(1)
hypothetical protein T06_9653 [Trichinella sp. T6]
KRZ86287.1 **Score:** 56 **Matches:** 1(1) **Sequences:** 1(1)
hypothetical protein T08_4685 [Trichinella sp. T8]

---

15. XP_012795120.1 **Score:** 56 **Matches:** 1(1) **Sequences:** 1(1) **emPAI:** 0.04
Unconventional myosin-XVIIIa [Schistosoma haematobium]
☐ Check to include this hit in error tolerant search

Query	Observed	Mr(expt)	Mr(calc)	Delta	Miss	Score	Expect	Rank	Unique	Peptide
614	703.8820	1405.7494	1405.7122	0.0372	1	56	0.036	3	U	R.IKELEAMSNDLK.C + Oxidation (M)

Proteins matching the same set of peptides:
XP_018651411.1 **Score:** 56 **Matches:** 1(1) **Sequences:** 1(1)
hypothetical protein Smp_172330 [Schistosoma mansoni]

---

16. PSN47327.1 **Mass:** 197418 **Score:** 55 **Matches:** 1(1) **Sequences:** 1(1) **emPAI:** 0.02
hypothetical protein C0J52_04406 [Blattella germanica]
☐ Check to include this hit in error tolerant search

	Query	Observed	Mr(expt)	Mr(calc)	Delta	Miss	Score	Expect	Rank	Unique	Peptide
☑	426	559.8320	1117.6494	1117.5880	0.0615	0	55	0.046	1	U	K.SSTWLQQIR.N

Peptide matches not assigned to protein hits: (no details means no match)

	Query	Observed	Mr(expt)	Mr(calc)	Delta	Miss	Score	Expect	Rank	Unique	Peptide
☑	304	474.2840	946.5534	946.5634	-0.0099	1	55	0.059	1		VALTLMKR + Oxidation (M)
☑	789	595.3720	1783.0942	1782.9476	0.1466	1	54	0.06	1		NKDPLNDTVVNLLGGSK
☑	750	564.9570	1691.8492	1691.7308	0.1184	0	54	0.053	1		LADEEDANSNLNEMK
☑	635	484.6130	1450.8172	1450.6939	0.1232	1	54	0.056	1		IAEKEEEFEATR
☑	743	839.0250	1676.0354	1675.8893	0.1461	0	54	0.055	1		GQNLNQVTYAVSALAK
☑	359	505.7890	1009.5634	1009.5080	0.0554	0	52	0.079	1		ATDQTFLSK
☑	317	487.3140	972.6134	972.5604	0.0531	1	52	0.095	1		IELSERVK
☑	228	421.7580	841.5014	841.5022	-0.0007	0	52	0.07	1		VATVSLPR
☑	696	786.9500	1571.8854	1571.7171	0.1683	0	52	0.077	1		GSDSSQLTMMTSISK
☑	230	421.7780	841.5414	841.5022	0.0393	0	52	0.071	1		VATVSLPR
☑	399	535.7870	1069.5594	1069.4862	0.0732	0	52	0.087	1		SLYQCGNTK
☑	452	586.8700	1171.7254	1171.6448	0.0806	1	52	0.092	1		LNAEIDELKK
☑	642	491.2970	1470.8692	1470.7718	0.0974	0	51	0.1	1		IAGADIEHYLLEK
☑	264	440.7760	879.5374	879.4372	0.1003	0	51	0.12	1		VGTEMVTK + Oxidation (M)
☑	607	698.3160	1394.6174	1394.6170	0.0005	0	50	0.13	1		ANMMSGEIEELR + Oxidation (M)
☑	727	549.3390	1644.9952	1644.8029	0.1923	0	50	0.13	1		LQGEIEDLMVDVER
☑	844	654.6850	1961.0332	1960.8796	0.1536	0	49	0.13	1		QNDSVNEMADQLDQLNK
☑	653	739.5230	1477.0314	1476.8704	0.1610	1	49	0.16	1		KVISYFAIVAAAPK
☑	319	489.7560	977.4974	977.4600	0.0375	1	49	0.19	1		QEAMKAER + Oxidation (M)
☑	344	497.7690	993.5234	993.4324	0.0910	0	49	0.19	1		SEEMEELK
☑	608	698.3220	1394.6294	1394.6170	0.0125	0	49	0.18	1		ANMMSGEIEELR + Oxidation (M)
☑	818	616.3890	1846.1452	1845.9625	0.1827	0	49	0.15	1		YSILAPNVIPEGFVDGR
☑	700	530.6460	1588.9162	1588.7944	0.1218	0	49	0.17	1		QLEEAESQISQLSK
☑	717	541.6490	1621.9252	1621.8060	0.1192	0	49	0.17	1		AQLEEAHEQIEAVR
☑	442	575.8100	1149.6054	1149.5335	0.0719	1	49	0.21	1		SEEMEELKR
☑	262	440.7570	879.4994	879.4372	0.0623	0	48	0.22	1		VGTEMVTK + Oxidation (M)
☑	273	451.7540	901.4934	901.4691	0.0243	1	48	0.28	1		MPEEKLR
☑	791	595.3880	1783.1422	1782.9476	0.1946	1	48	0.26	1		NKDPLNDTVVNLLGGSK
☑	867	686.4220	2056.2442	2056.0436	0.2006	1	48	0.19	1		KLEQDINELEVSLDAANR
☑	382	522.8240	1043.6334	1043.5499	0.0836	0	47	0.33	1		LNAEIDELK

☑	466	598.3440	1194.6734	1194.6105	0.0629	0	46	0.33	1	GVSAAPAGAGPANR
☑	773	587.0330	1758.0772	1757.9564	0.1208	0	46	0.32	1	DALVSQLFVPVVAESGK
☑	385	523.3200	1044.6254	1044.5451	0.0803	0	46	0.43	1	EVEIGGLNSK
☑	542	658.3820	1314.7494	1314.6166	0.1329	0	46	0.38	1	MFVLEQEEYK
☑	284	462.2490	922.4834	922.4396	0.0439	0	46	0.4	1	ELANFDSK
☑	652	493.3420	1477.0042	1476.8704	0.1338	1	46	0.36	1	KVISYFAIVAAAPK
☑	309	477.7820	953.5494	953.4892	0.0603	0	45	0.38	1	IDMTSFIK
☑	484	616.3660	1230.7174	1230.5116	0.2058	0	45	0.47	1	TMMMMAATAEK + Oxidation (M)
☑	229	421.7700	841.5254	841.5022	0.0233	0	45	0.38	1	VATVSLPR
☑	272	451.7490	901.4834	901.4869	-0.0034	1	45	0.58	1	DIEEKLR
☑	496	417.6160	1249.8262	1249.7070	0.1191	0	45	0.5	1	FPIYTAQVALK
☑	712	540.9680	1619.8822	1619.7638	0.1183	0	44	0.54	1	LDETTNQLSEQASGK
☑	687	519.9790	1556.9152	1556.8046	0.1106	0	43	0.66	1	ELEEQNVTVLQQK
☑	644	736.4450	1470.8754	1470.7718	0.1036	0	43	0.74	1	IAGADIEHYLLEK
☑	738	557.6620	1669.9642	1669.8159	0.1483	0	43	0.69	1	AEVDDLHSQLESLSK
☑	251	438.2490	874.4834	874.4257	0.0578	1	43	0.97	1	DAGKGANSR
☑	328	490.3140	978.6134	978.5109	0.1025	0	42	0.9	1	MFGWLVAR
☑	274	451.7560	901.4974	901.4869	0.0106	1	42	0.99	1	DIEEKLR
☑	433	566.3310	1130.6474	1130.5608	0.0867	0	42	0.91	1	NVIWVEDEK
☑	490	622.4100	1242.8054	1242.6204	0.1851	0	42	0.99	1	LNDAIADELNR
☑	303	474.2840	946.5534	946.4794	0.0741	1	42	1.2	1	DVTMKEPK
☑	707	534.5920	1600.7542	1600.8420	-0.0878	1	42	0.93	1	TVEAQLQSLEAEKR
☑	253	438.7420	875.4694	875.4786	-0.0092	1	41	1.3	1	MKEIVEK
☑	237	428.7710	855.5274	855.5066	0.0209	0	41	0.94	1	IATVIDPK
☑	375	516.3430	1030.6714	1030.6175	0.0539	0	41	1.3	1	VAFLLGVNAK
☑	552	665.8780	1329.7414	1329.6161	0.1254	0	41	1.2	1	EVQGQLEDEQR
☑	794	596.3320	1785.9742	1785.8745	0.0997	0	40	1.1	1	LEEQDGVNAQQVDLTK
☑	324	490.2920	978.5694	978.5109	0.0585	0	40	1.5	1	MFGWLVAR
☑	417	552.3010	1102.5874	1102.4713	0.1162	0	40	1.5	1	AMNDHATEAK + Oxidation (M)
☑	358	505.7870	1009.5594	1009.5080	0.0514	0	40	1.4	1	ATDQTFLSK
☑	231	421.7880	841.5614	841.5022	0.0593	0	40	1.2	1	VATVSLPR
☑	498	628.3690	1254.7234	1254.6092	0.1143	0	40	1.5	1	VSTSEIYETAR
☑	560	669.8860	1337.7574	1337.6551	0.1024	0	40	1.5	1	GGSFLTVSFMHR
☑	320	489.7660	977.5174	977.4964	0.0211	1	39	2	1	SVAMKAAER + Oxidation (M)
☑	529	434.2680	1299.7822	1299.7146	0.0676	1	39	2	1	QLNLSNNKLEK
☑	482	616.3370	1230.6594	1230.5116	0.1478	0	38	2.1	1	TMMMMAATAEK + Oxidation (M)
☑	299	472.7630	943.5114	943.4723	0.0391	0	38	3	1	EGNPVSSVR
☑	247	435.7920	869.5694	869.4971	0.0724	0	38	2.2	1	ELGVSLPR
☑	485	616.3670	1230.7194	1230.6204	0.0991	0	38	2.5	1	VAAEAEAAATATR
☑	501	631.3530	1260.6914	1260.5833	0.1081	0	38	2.5	1	TQEALQAEEDK
☑	848	660.0340	1977.0802	1976.8745	0.2056	0	38	2	1	QNDSVNEMADQLDQLNK + Oxidation (M)
☑	509	633.3770	1264.7394	1264.5969	0.1426	0	37	2.7	1	TNAELSECTLK
☑	356	505.7550	1009.4954	1009.4022	0.0932	0	37	2.7	1	EEMEEATR + Oxidation (M)
☑	587	684.8640	1367.7134	1367.6317	0.0818	0	37	2.7	1	IQHEIEDAEER
☑	845	656.3890	1966.1452	1965.9755	0.1696	1	36	2.7	1	TQEALQAEEDKVNHLNK
☑	420	555.2710	1108.5274	1108.5189	0.0085	0	36	3.5	1	SVEAFNEWK
☑	460	592.8460	1183.6774	1183.7176	-0.0401	1	36	3.5	1	NVELDKIIIK
☑	238	428.7760	855.5374	855.5178	0.0197	0	36	3	1	LATVSLPR
☑	799	598.9970	1793.9692	1793.9094	0.0598	1	36	3.4	1	IIYGGSVSGANCKELAR
☑	3	403.1540	1206.4402	1206.5914	-0.1512	1	36	6.9	1	MATDDEIIRK + Oxidation (M)
☑	72	519.3140	1036.6134	1036.5077	0.1058	0	35	8.7	1	VLEQEEYK
☑	588	456.9160	1367.7262	1367.6317	0.0945	0	35	4.5	1	IQHEIEDAEER
☑	549	444.2320	1329.6742	1329.6161	0.0581	0	34	5.4	1	EVQGQLEDEQR
☑	326	490.2950	978.5754	978.5109	0.0645	0	34	6.2	1	MFGWLVAR
☑	350	503.7860	1005.5574	1005.4953	0.0621	0	34	6.5	1	CIVPNEFK
☑	252	438.2490	874.4834	874.4760	0.0075	0	34	7.1	1	ITAENSLK
☑	361	506.7610	1011.5074	1011.4444	0.0631	0	34	5.7	1	NQSTEMFR
☑	422	556.3170	1110.6194	1110.5305	0.0889	0	34	5.7	1	DDAQQVNPPK
☑	464	597.8380	1193.6614	1193.5532	0.1082	1	34	5.9	1	MREEMTELR
☑	357	505.7640	1009.5134	1009.5080	0.0054	0	34	5.9	1	ATDQTFLSK
☑	622	474.2560	1419.7462	1419.7431	0.0030	0	34	6.4	1	LSYMLEALQGPAK
☑	338	493.3300	984.6454	984.6331	0.0123	1	34	6.8	1	KQLLSIGVK
☑	406	539.2970	1076.5794	1076.5098	0.0697	1	33	7.2	1	TSDAAKEAER
☑	715	541.6420	1621.9042	1621.7696	0.1346	1	33	5.8	1	KFADSQAELENSQR
☑	548	444.2310	1329.6712	1329.6347	0.0365	0	33	6.7	1	NMTLSELVDHR + Oxidation (HW)
☑	250	438.2460	874.4774	874.4046	0.0729	0	33	8.2	1	WGQNQSR
☑	321	489.7700	977.5254	977.4964	0.0291	1	33	7.9	1	SVAMKAAER + Oxidation (M)
☑	285	462.2490	922.4834	922.4396	0.0439	0	33	7.8	1	ELANFDSK
☑	709	805.9360	1609.8574	1609.7460	0.1115	0	33	6.7	1	FHCLAHGTSVYYR
☑	662	498.2660	1491.7762	1491.6875	0.0887	0	33	7.2	1	AMESQQASLEAEAK

☑	402	538.2910	1074.5674	1074.5305	0.0369	0	33	8.7	1	SSTPAQVTER
☑	494	625.9180	1249.8214	1249.7070	0.1144	0	33	8.1	1	FPIYTAQVALK
☑	287	462.2630	922.5114	922.4396	0.0719	0	32	8.8	1	ELANFDSK
☑	778	587.0520	1758.1342	1757.9564	0.1778	0	32	7.2	1	DALVSQLFVPVVAESGK
☑	279	459.2540	916.4934	916.4364	0.0570	0	32	10	1	MLYFESK
☑	266	444.2900	886.5654	886.4946	0.0708	0	32	9.9	1	MAIAVAPAK + Oxidation (M)
☑	627	714.3780	1426.7414	1426.6762	0.0652	1	32	8.6	1	KMSTSFHTVETK + Oxidation (HW); Oxidation (M)
☑	584	456.2650	1365.7732	1365.7252	0.0480	0	32	8.5	1	GLETQIQELHAK
☑	798	897.9830	1793.9514	1793.9386	0.0128	1	32	7.8	1	FKTLMSFFSLSTLTR + Oxidation (M)
☑	366	508.8300	1015.6454	1015.5848	0.0606	1	32	11	1	LLEAVKGMR
☑	459	592.6800	1183.3454	1183.5729	-0.2275	1	31	11	1	FNKLMGEAMK + Oxidation (M)
☑	664	498.2730	1491.7972	1491.6875	0.1097	0	31	11	1	AMESQQASLEAEAK
☑	868	688.8650	2063.5732	2064.0132	-0.4401	0	31	9.3	1	NCPHVAPPSLEMIQQTVK + Oxidation (M)
☑	239	430.7440	859.4734	859.4188	0.0546	0	31	15	1	IHFDNSK
☑	390	530.2890	1058.5634	1058.5356	0.0278	1	30	15	1	SENPSKLER
☑	405	538.8090	1075.6034	1075.5080	0.0954	0	30	15	1	ANGMASQLER
☑	540	657.3490	1312.6834	1312.7788	-0.0954	0	30	13	1	IEGLIAAIILCK
☑	645	737.3930	1472.7714	1472.6994	0.0720	0	30	13	1	IQELEEDLEAER
☑	492	625.3320	1248.6494	1248.5696	0.0798	0	30	14	1	SFDVPPPSMEK + Oxidation (M)
☑	634	484.6080	1450.8022	1450.6939	0.1082	1	30	13	1	IAEKEEEFEATR
☑	880	789.7830	2366.3272	2366.1517	0.1754	1	30	11	1	LKQFFNHHMFVLEQEEYK
☑	1	401.1200	1200.3382	1200.5128	-0.1746	1	30	28	1	GMSRGMGGGGYR + Oxidation (M)
☑	532	651.9050	1301.7954	1301.6827	0.1128	1	30	16	1	LSDIDQEKQVK
☑	875	749.7410	2246.2012	2246.0736	0.1276	1	29	11	1	KLEADLAAMQADLEEAANEAK + Oxidation (M)
☑	619	471.9100	1412.7082	1412.6098	0.0984	0	29	16	1	CTTSICNLGEMK
☑	94	543.3940	1627.1602	1626.8940	0.2661	0	29	32	1	LVGESGSAAAVAAAALAAK
☑	623	710.8860	1419.7574	1419.7101	0.0473	1	29	17	1	IKELEAQMEAMK
☑	410	545.8010	1089.5874	1089.5488	0.0386	0	29	19	1	EAMQVSANLK
☑	834	629.7550	1886.2432	1886.0513	0.1919	1	29	14	1	DALVSQLFVPVVAESGKK
☑	651	738.4320	1474.8494	1474.8395	0.0099	1	29	16	1	YPLTDVAGAVLKTK
☑	233	424.2640	846.5134	846.4447	0.0688	0	29	18	1	ESIETIR
☑	282	461.7460	921.4774	921.4807	-0.0033	1	29	18	1	KIEEEFK
☑	609	698.8580	1395.7014	1395.6881	0.0133	1	29	17	1	NYEESVEASKLK
☑	294	467.2510	932.4874	932.4313	0.0561	0	29	22	1	MIYSEFK + Oxidation (M)
☑	313	480.2650	958.5154	958.4872	0.0282	0	29	23	1	LYDNHLGK
☑	678	512.6160	1534.8262	1534.7297	0.0965	1	29	18	1	AGTLASLEDMRDEK
☑	493	625.8860	1249.7574	1249.6886	0.0688	1	29	19	1	MLLSVVSMRAK + Oxidation (M)
☑	349	503.7790	1005.5434	1005.4727	0.0707	0	29	22	1	EGSVSGSEVR
☑	378	516.3710	1030.7274	1030.6175	0.1099	0	29	22	1	VAFLLGVNAK
☑	322	490.2860	978.5574	978.5433	0.0142	1	29	22	1	MFRSLVAR
☑	800	599.0020	1793.9842	1793.9094	0.0748	1	28	17	1	IIYGGSVSGANCKELAR
☑	178	643.4080	1927.2022	1926.9774	0.2248	1	28	37	1	FHPSLELMQRIYPHK + 2 Oxidation (HW)
☑	481	615.3890	1228.7634	1228.6088	0.1547	0	28	22	1	ENVYHVEPVK + Oxidation (HW)
☑	681	513.4730	1537.3972	1536.9425	0.4547	0	28	20	1	APLLLLLALACTLR
☑	579	455.9170	1364.7292	1364.6606	0.0686	0	28	21	1	SLESEALVTMNR + Oxidation (M)
☑	606	463.8920	1388.6542	1388.4964	0.1578	0	28	23	1	EHSANDDDDDEK
☑	850	661.7200	1982.1382	1982.0143	0.1239	1	28	18	1	LLSSTSGKSVMGDFVLEGR
☑	518	426.2420	1275.7042	1275.5943	0.1099	0	28	24	1	ATQETVDDLER
☑	456	590.3410	1178.6674	1178.6884	-0.0209	1	28	24	1	VVAAHGKITAGR
☑	820	617.0130	1848.0172	1847.8934	0.1237	1	28	20	1	AKLESTLDEMEENLAR
☑	870	697.3880	2089.1422	2089.1349	0.0073	1	27	20	1	LMLKGMPQMLAALSAILEK + 2 Oxidation (M)
☑	387	526.3130	1050.6114	1050.4692	0.1423	0	27	28	1	MDEIWETK
☑	776	587.0430	1758.1072	1757.9564	0.1508	0	27	27	1	DALVSQLFVPVVAESGK
☑	830	622.3690	1864.0852	1863.8884	0.1968	1	27	25	1	AKLESTLDEMEENLAR + Oxidation (M)
☑	534	436.9170	1307.7292	1307.6735	0.0557	0	27	30	1	NLHSTSPHFIR
☑	541	438.7650	1313.2732	1313.7442	-0.4710	1	27	34	1	DVLELKEIDLK
☑	427	560.5780	1119.1414	1119.6037	-0.4622	1	26	36	1	RGDTFIQGVK
☑	254	438.7460	875.4774	875.4786	-0.0012	1	26	40	1	MKEIDIK
☑	37	465.5600	929.1054	929.4831	-0.3777	1	26	74	1	HRYEGLR
☑	220	411.2330	820.4514	820.4225	0.0289	1	26	24	1	RGAMAATK + Oxidation (M)
☑	307	476.2860	950.5574	950.5259	0.0315	1	26	37	1	MTSFKLPK
☑	255	439.2620	876.5094	876.4124	0.0971	0	26	40	1	VSTCTGPR
☑	483	616.3620	1230.7094	1230.5116	0.1978	0	26	37	1	TMMMMAATAEK + Oxidation (M)
☑	491	415.5790	1243.7152	1243.7023	0.0128	0	26	37	1	TISEIEIAIQK
☑	418	552.3120	1102.6094	1102.4713	0.1381	0	26	41	1	AMNDHATEAK + Oxidation (M)
☑	612	469.5740	1405.7002	1405.6395	0.0607	0	26	35	1	IQELEAECESAK
☑	421	556.3120	1110.6094	1110.6186	-0.0091	1	26	38	1	KNVLHYPPK + Oxidation (HW)
☑	352	504.8490	1007.6834	1007.4342	0.2492	0	26	42	1	MEGGGTTEAR
☑	561	446.9270	1337.7592	1337.6000	0.1592	1	25	38	1	DDAREFWDIR + Oxidation (HW)
☑	430	563.3100	1124.6054	1124.5648	0.0406	0	25	42	1	HDLLMLHSK + 2 Oxidation (HW)

☑	550	665.8650	1329.7154	1329.6161	0.0994	0	25	42	1	EVQGQLEDEQR
☑	369	510.7840	1019.5534	1019.5359	0.0175	1	25	48	1	LSSSGKQSAR
☑	409	544.8180	1087.6214	1087.5008	0.1206	0	25	49	1	FGSMENLFK + Oxidation (M)
☑	135	579.2320	1734.6742	1734.8788	-0.2046	1	25	82	1	EERISVEEYNQIVK
☑	626	713.9280	1425.8414	1425.7292	0.1122	1	25	41	1	YKQLYVPDWSK
☑	81	530.6510	1059.2874	1059.5237	-0.2362	0	25	91	1	DVIDIDWGK
☑	337	493.2670	984.5194	984.5392	-0.0198	1	25	49	1	DKPPAWKK + Oxidation (HW)
☑	444	575.8740	1149.7334	1149.5886	0.1449	1	25	48	1	SMCPAVITKK + Oxidation (M)
☑	595	458.5600	1372.6582	1372.6883	-0.0302	0	25	49	1	QLFMTGGVVMFK + Oxidation (M)
☑	363	508.7990	1015.5834	1015.5525	0.0310	0	25	58	1	LLEMFHVK
☑	308	476.7010	951.3874	951.4484	-0.0609	0	24	52	1	TNAWVSMK + Oxidation (HW)
☑	745	559.7140	1676.1202	1675.8893	0.2309	0	24	47	1	GQNLNQVTYAVSALAK
☑	808	608.3710	1822.0912	1821.9084	0.1828	0	24	45	1	LLMDLFVGSTGGFNAHK + Oxidation (HW)
☑	263	440.7620	879.5094	879.4120	0.0974	1	24	55	1	QDSKQMK + Oxidation (M)
☑	396	533.2960	1064.5774	1064.5172	0.0603	1	24	57	1	MNTEDLAKK + Oxidation (M)
☑	77	522.2250	1042.4354	1042.4614	-0.0260	1	24	1.1e+02	1	NCHQKNDK
☑	691	522.0010	1562.9812	1562.8205	0.1607	1	24	51	1	QATPAFIQQFKER
☑	438	571.3100	1140.6054	1140.5923	0.0132	0	24	56	1	FLTMIEMLK + Oxidation (M)
☑	497	625.9330	1249.8514	1249.6781	0.1734	0	24	57	1	MPLFVWSIII + Oxidation (HW); Oxidation (M)
☑	864	681.1260	2040.3562	2039.9596	0.3966	1	24	46	1	VIERHGYHAGMHGSGLQK + 3 Oxidation (HW); Oxidation (M)
☑	593	687.3290	1372.6434	1372.7211	-0.0777	1	24	58	1	TYGLAVAREHTR
☑	852	662.2790	1983.8152	1983.9816	-0.1664	1	24	46	1	LYRIHNHWEIHHGPK + 3 Oxidation (HW)
☑	216	404.2270	806.4394	806.4286	0.0108	0	24	34	1	IAAETFR
☑	771	586.9960	1757.9662	1757.8559	0.1103	1	24	54	1	LQYVHKYVCTYER
☑	226	420.7460	839.4774	839.4501	0.0273	0	24	42	1	HSELQVK
☑	379	517.3010	1032.5874	1032.5465	0.0410	0	24	71	1	TIIHAPGHR + 2 Oxidation (HW)
☑	556	444.5420	1330.6042	1330.6364	-0.0323	0	24	63	1	GTEEELGEALQR
☑	616	703.9550	1405.8954	1405.8082	0.0873	1	24	59	1	RFPIYTAQVALK
☑	487	413.8720	1238.5942	1238.5826	0.0116	1	24	64	1	QGRIVQPECH + Oxidation (HW)
☑	310	479.2680	956.5214	956.5113	0.0101	1	23	70	1	SEMPPLRK
☑	414	551.8080	1101.6014	1101.5818	0.0196	1	23	72	1	DNSVKLWGAL
☑	649	492.2640	1473.7702	1473.6834	0.0867	0	23	63	1	IEELEEEVEAER
☑	351	503.8010	1005.5874	1005.4880	0.0995	0	23	76	1	GAFPDSVSAR
☑	814	612.6660	1834.9762	1834.8917	0.0845	1	23	55	1	SDTVTQPTLEMRQAMK
☑	516	638.8330	1275.6514	1275.5943	0.0572	0	23	71	1	ATQETVDDLER
☑	665	499.2360	1494.6862	1494.7864	-0.1003	1	23	65	1	LKMYQTNGSVLNK
☑	762	577.3410	1729.0012	1728.8610	0.1401	1	23	65	1	DSEDIYLPFDKFLK
☑	680	769.3880	1536.7614	1536.8334	-0.0719	1	23	72	1	EMRSLASVQILFK + Oxidation (M)
☑	589	456.9160	1367.7262	1367.5809	0.1453	1	23	75	1	RDSMEMLSDER
☑	793	595.6940	1784.0602	1783.8271	0.2330	0	23	67	1	GGVGGAVCAGGAAAAGAEPER
☑	158	605.8430	1209.6714	1209.8060	-0.1346	0	22	1.4e+02	1	LITIVIQGIIK
☑	83	531.6610	1591.9612	1591.7015	0.2596	0	22	1.7e+02	1	SHSAPGWDGTYINR + 2 Oxidation (HW)
☑	479	611.0700	1220.1254	1220.5898	-0.4643	0	22	84	1	YINNNVTNNR
☑	594	458.5560	1372.6462	1372.6115	0.0347	1	22	81	1	CRSAYDDLVMK + Oxidation (M)
☑	735	555.2920	1662.8542	1662.7883	0.0659	0	22	74	1	SISNDGIVTMDQLDR
☑	340	496.2550	990.4954	990.5056	-0.0101	1	22	1e+02	1	KLEMTPEK + Oxidation (M)
☑	568	450.2420	1347.7042	1347.7146	-0.0105	1	22	90	1	KLAGWTGSSSNLK
☑	838	637.6870	1910.0392	1909.8177	0.2215	1	22	71	1	KGNFENSDEELDQEEK
☑	218	405.2250	808.4354	808.3636	0.0718	0	22	43	1	LAEMSDK + Oxidation (M)
☑	370	510.8080	1019.6014	1019.5321	0.0693	0	22	1e+02	1	ISSVCDVIK
☑	104	549.7810	1646.3212	1645.9191	0.4020	1	22	1.8e+02	1	AYGITNKIELHFIK
☑	411	546.3330	1090.6514	1090.5229	0.1285	1	22	1e+02	1	YHLMPDKR + Oxidation (HW); Oxidation (M)
☑	828	619.9340	1856.7802	1856.9956	-0.2154	1	22	78	1	QTVENAIGKSISDLLNR
☑	806	604.0070	1808.9992	1808.9230	0.0762	1	22	79	1	DLDAMVFLISKLSDDK
☑	628	715.3530	1428.6914	1428.6047	0.0867	0	22	93	1	CTTSICNLGEMK + Oxidation (M)
☑	398	535.7330	1069.4514	1069.5040	-0.0525	0	22	1e+02	1	QYISSGSNSK
☑	325	490.2940	978.5734	978.4593	0.1142	0	21	1.1e+02	1	MFGNQSPAK
☑	851	661.9640	1982.8702	1983.1880	-0.3179	1	21	86	1	EVELILLILRNVGFSLR
☑	874	747.1150	2238.3232	2238.2461	0.0771	1	21	77	1	MWIPIMPDLIPLKFIFHK
☑	775	587.0420	1758.1042	1757.9564	0.1478	0	21	94	1	DALVSQLFVPVVAESGK
☑	283	462.2330	922.4514	922.4794	-0.0279	1	21	1.2e+02	1	KLVSMDSK + Oxidation (M)
☑	679	512.6290	1534.8652	1534.7926	0.0726	1	21	1e+02	1	AGGMNLDKLAYNLR
☑	474	606.7560	1211.4974	1211.6510	-0.1536	1	21	1.1e+02	1	SSDPTHVKTLK
☑	105	550.9470	1649.8192	1649.7645	0.0546	1	21	2.2e+02	1	DELDLEWRTGTSGR + Oxidation (HW)
☑	730	550.6440	1648.9102	1648.8380	0.0722	0	21	1e+02	1	ASSISSATTVAAAASQAR
☑	318	489.7230	977.4314	977.5328	-0.1013	1	21	1.3e+02	1	KLSLMSQR + Oxidation (M)
☑	332	491.3070	980.5994	980.5039	0.0955	1	21	1.2e+02	1	RSTLEGYR
☑	801	599.0130	1794.0172	1793.9961	0.0211	1	21	1e+02	1	MEAVFVTKPSTLVTKK + Oxidation (M)
☑	301	472.7860	943.5574	943.4876	0.0699	1	21	1.5e+02	1	WNTGGKGPK
☑	315	484.8110	967.6074	967.4909	0.1165	0	21	1.1e+02	1	IIHANPCK + Oxidation (HW)

☑	604	691.4030	1380.7914	1380.6714	0.1200	0	21	1.2e+02	1	GLGDWYVELWK + Oxidation (HW)
☑	784	591.7630	1772.2672	1771.7831	0.4841	0	21	1e+02	1	GDGDIVTSPMMDIFMK + Oxidation (M)
☑	667	499.3270	1494.9592	1494.6443	0.3149	0	21	1.1e+02	1	TIGMMAEDGTNGGNK
☑	470	601.3730	1200.7314	1200.6098	0.1216	0	21	1.3e+02	1	QSVEADINGLR
☑	222	413.3090	824.6034	824.3889	0.2145	0	21	76	1	LGHGNGDR
☑	372	513.2620	1024.5094	1024.5189	-0.0095	0	21	1.3e+02	1	SIFSTTNAGK
☑	547	441.8920	1322.6542	1322.5950	0.0592	1	20	1.3e+02	1	ADGDTDSNVSKSK
☑	339	495.2440	988.4734	988.4106	0.0628	0	20	1.5e+02	1	AHMPDQMK + 2 Oxidation (M)
☑	857	670.4730	2008.3972	2007.9619	0.4353	1	20	1.1e+02	1	DFTTHLGLMRASTMGAQR + Oxidation (M)
☑	710	538.1000	1611.2782	1610.7942	0.4840	1	20	1.2e+02	1	LLAMRMMTTNELR + 2 Oxidation (M)
☑	276	453.2440	904.4734	904.4073	0.0662	0	20	1.6e+02	1	SGPAPMSSR + Oxidation (M)
☑	199	692.8210	2075.4412	2075.0582	0.3830	1	20	2.4e+02	1	HQQMSDLHKQNLENLIK
☑	306	474.7970	947.5794	947.5189	0.0606	0	20	1.6e+02	1	GNITIQFR
☑	186	657.6390	1313.2634	1312.8078	0.4556	0	20	2.7e+02	1	ASLVILISGAITR
☑	774	880.0510	1758.0874	1757.9564	0.1311	0	20	1.3e+02	1	DALVSQLFVPVVAESGK
☑	305	474.3010	946.5874	946.5335	0.0539	0	20	1.8e+02	1	DVTVVISSK
☑	167	625.8960	1249.7774	1249.6190	0.1584	1	20	2.7e+02	1	EDIEKDFLNK
☑	428	561.8710	1121.7274	1121.5254	0.2020	0	20	1.5e+02	1	FSNIEFHGR + Oxidation (HW)
☑	640	490.7880	1469.3422	1469.7296	-0.3875	1	20	1.4e+02	1	LENGDPKCPATIR
☑	656	743.4180	1484.8214	1484.8061	0.0153	1	20	1.4e+02	1	RLFTSLMGALTFL + Oxidation (M)
☑	293	466.7660	931.5174	931.4723	0.0452	1	20	1.8e+02	1	ESAVEKNR
☑	621	472.8020	1415.3842	1415.7085	-0.3243	1	20	1.5e+02	1	KSEYFIPFSNGK
☑	546	441.8840	1322.6302	1322.6024	0.0278	1	20	1.5e+02	1	EISMQSEEDKK
☑	658	496.3000	1485.8782	1485.7683	0.1098	1	20	1.4e+02	1	IADTMMPLHVAKK + Oxidation (HW); Oxidation (M)
☑	728	550.5660	1648.6762	1648.9334	-0.2572	1	20	1.4e+02	1	MPQIPEAIIRQLPK + Oxidation (M)
☑	824	618.3450	1852.0132	1851.9227	0.0904	0	20	1.3e+02	1	DAVIGRPASEFAHPTER
☑	657	496.0470	1485.1192	1484.6790	0.4401	1	20	1.5e+02	1	QCEHRVTSSHTK + Oxidation (HW)
☑	786	594.3800	1780.1182	1779.7668	0.3513	0	20	1.3e+02	1	LEAFGSGGMMINDNHR + Oxidation (HW); Oxidation (M)
☑	449	582.4160	1162.8174	1162.5540	0.2635	0	19	1.7e+02	1	VEPQSDLTMK + Oxidation (M)
☑	240	430.7590	859.5034	859.4188	0.0846	0	19	1.9e+02	1	IHFDNSK
☑	858	672.1430	2013.4072	2012.9414	0.4657	1	19	1.3e+02	1	ESLPKAWGPQSSNYFMR + Oxidation (M)
☑	97	544.1870	1629.5392	1629.7933	-0.2541	1	19	3.3e+02	1	IFDSTSKHHAVNMK + Oxidation (M)
☑	207	751.5530	2251.6372	2252.1372	-0.5000	1	19	3.1e+02	1	IVRPNATRDESTEMFFLAR
☑	232	423.7180	845.4214	845.4243	-0.0029	0	19	1.9e+02	1	QSTPTAGGK
☑	882	791.3150	2370.9232	2371.2168	-0.2936	0	19	1.2e+02	1	GTGGALVLVMYDEIQMLLFGTK + Oxidation (M)
☑	185	656.5970	1966.7692	1966.8545	-0.0853	0	19	3e+02	1	FAGSGPASEEEEDVFGSPR
☑	768	586.3000	1755.8782	1756.0359	-0.1577	1	19	1.4e+02	1	AKPPQPSKKPAAAAVPAK
☑	641	491.2920	1470.8542	1470.8493	0.0049	1	19	1.6e+02	1	AIVIMTGVWRIGR
☑	225	419.1880	836.3614	836.4617	-0.1002	0	19	1.1e+02	1	SRPPAGPR
☑	723	546.3070	1635.8992	1635.8072	0.0920	1	19	1.6e+02	1	ALEERMLMLTGANR + 2 Oxidation (M)
☑	408	543.8150	1085.6154	1085.4600	0.1554	0	19	1.8e+02	1	TFTNWCNK + Oxidation (HW)
☑	757	570.2930	1707.8572	1707.7257	0.1315	0	19	1.5e+02	1	LADEEDANSNLNEMK + Oxidation (M)
☑	846	656.4130	1966.2172	1965.9558	0.2614	0	19	1.4e+02	1	QTHNHANGAPQFTYKPR
☑	599	459.0910	1374.2512	1374.7065	-0.4553	0	19	1.7e+02	1	VAVMIEEVGGLDK + Oxidation (M)
☑	813	611.4420	1831.3042	1830.9696	0.3346	0	19	1.5e+02	1	CVRPVLSDIQVMASIK + Oxidation (M)
☑	807	607.6650	1819.9732	1819.9754	-0.0022	1	19	1.5e+02	1	VITPSMDKNSDIFIIK
☑	510	633.4010	1264.7874	1264.6061	0.1813	1	19	1.8e+02	1	HDPIGYDRHR
☑	329	490.7970	979.5794	979.5127	0.0667	0	19	1.8e+02	1	WAAQTFLK + Oxidation (HW)
☑	863	679.4320	2035.2742	2034.8874	0.3868	0	19	1.5e+02	1	TDEQIGEPEMCDLELTR
☑	288	464.0140	926.0134	926.4821	-0.4687	0	19	1.6e+02	1	QAPEVVER
☑	193	673.6930	1345.3714	1345.6514	-0.2799	1	19	3.6e+02	1	SYLYTSEAEKR
☑	214	401.7480	801.4814	801.4895	-0.0080	1	19	1.6e+02	1	GVKVMLR
☑	714	541.6290	1621.8652	1621.7988	0.0664	0	19	1.7e+02	1	QAIEEEWLSNIFK + Oxidation (HW)
☑	311	479.2780	956.5414	956.4208	0.1206	1	19	2.1e+02	1	DTMFCRK
☑	61	505.6670	1009.3194	1009.3811	-0.0617	0	19	3.6e+02	1	GGCDWELGT + Oxidation (HW)
☑	630	484.5670	1450.6792	1450.8144	-0.1352	1	19	1.8e+02	1	AVKDALSVPPAEVR
☑	860	674.3720	2020.0942	2019.9459	0.1483	1	19	1.6e+02	1	TEILPVKDDPDSFEEMR
☑	374	516.3400	1030.6654	1030.5910	0.0744	0	18	2.3e+02	1	LVDELTLTK
☑	383	523.2990	1044.5834	1044.5564	0.0271	1	18	2.3e+02	1	EASPRSAVTK
☑	761	577.3250	1728.9532	1728.8406	0.1126	0	18	1.8e+02	1	FEHMGHAPPPGQPLAK + Oxidation (HW)
☑	436	568.3040	1134.5934	1134.4984	0.0950	0	18	2.3e+02	1	MHVGMEMIR + Oxidation (HW); Oxidation (M)
☑	854	665.5260	1993.5562	1994.0077	-0.4516	1	18	1.6e+02	1	MAPDLRSSQLILVYCGR + Oxidation (M)
☑	803	601.4920	1801.4542	1801.8218	-0.3676	0	18	1.7e+02	1	ATYSDSIGNIETTDSTK
☑	265	441.2800	880.5454	880.5171	0.0284	0	18	1.8e+02	1	VITVFFR
☑	781	589.3910	1765.1512	1764.8312	0.3200	1	18	1.8e+02	1	NIDKVDNVTSSASMER
☑	249	438.2300	874.4454	874.5124	-0.0669	1	18	2.6e+02	1	KDATSLLK
☑	281	461.4170	920.8194	920.4385	0.3809	0	18	2.4e+02	1	AAANSMGTAK
☑	74	521.2260	1560.6562	1560.6768	-0.0206	1	18	4.1e+02	1	AMMKLGAESAAMMR + 4 Oxidation (M)
☑	415	551.8190	1101.6234	1101.5600	0.0634	0	18	2.4e+02	1	MQNELAQIR
☑	489	414.2630	1239.7672	1239.6030	0.1642	1	18	2.1e+02	1	SPRMSPTPSHK + Oxidation (HW)

☑	747	563.3170	1686.9292	1686.9086	0.0205	1	18	2e+02	1	AETITAAVNLIMERR
☑	666	499.2850	1494.8332	1494.7791	0.0541	0	18	2.1e+02	1	QQQQQPQPTTIAK
☑	624	710.9240	1419.8334	1419.6929	0.1406	1	18	2.3e+02	1	DFHMIQQSKVR + Oxidation (HW); Oxidation (M)
☑	770	586.4400	1756.2982	1755.8322	0.4659	1	18	1.9e+02	1	TPTSTMTRSHSPNSPR
☑	603	690.8840	1379.7534	1379.5623	0.1911	0	18	2.2e+02	1	DNGGQMTDEEIR + Oxidation (M)
☑	739	557.9690	1670.8852	1670.9203	-0.0351	1	18	2e+02	1	SISLKDVALAEQELR
☑	545	440.9180	1319.7322	1319.7449	-0.0127	1	18	2.3e+02	1	ESLVTFKNAIAK
☑	637	488.7230	1463.1472	1463.6059	-0.4588	0	18	2.2e+02	1	CTGSGNGDADGGGALR
☑	4	408.2610	1221.7612	1221.5270	0.2341	1	18	4.1e+02	1	NYYTSMCRK
☑	725	547.3690	1639.0852	1638.8148	0.2704	0	18	2.1e+02	1	HVSSGSTALHIAAGMGK + Oxidation (M)
☑	204	721.3350	2160.9832	2160.8946	0.0885	0	18	4.2e+02	1	EWTTSAGDFETHDPMYVK + 2 Oxidation (HW); Oxidation (M)
☑	777	587.0510	1758.1312	1757.9564	0.1748	0	18	2.1e+02	1	DALVSQLFVPVVAESGK
☑	575	677.8920	1353.7694	1353.6387	0.1307	0	18	2.3e+02	1	VTWMYPEGALR + Oxidation (HW); Oxidation (M)
☑	586	456.9050	1367.6932	1367.6728	0.0204	0	18	2.3e+02	1	TVAAIQQACHNR
☑	668	499.6080	1495.8022	1495.7266	0.0755	1	18	2.2e+02	1	KIQHEIEDAEER
☑	84	533.1510	1064.2874	1064.5880	-0.3005	0	18	4.6e+02	1	LHHFVAGLR + Oxidation (HW)
☑	196	686.4860	2056.4362	2055.9457	0.4904	1	18	4.4e+02	1	SNSEVDSTQAVYGEATSRR
☑	590	685.3940	1368.7734	1368.8340	-0.0606	1	18	2.3e+02	1	NTLKQLLALVEK
☑	191	666.7100	1997.1082	1996.9101	0.1980	1	18	4.4e+02	1	DPSKLCHYYHINQYK + 2 Oxidation (HW)
☑	578	455.9120	1364.7142	1364.6978	0.0164	0	18	2.3e+02	1	AGVMGLSMSMIIR
☑	716	541.6470	1621.9192	1621.8974	0.0218	1	18	2.2e+02	1	KAMEVLNLHQKPAK + Oxidation (M)
☑	877	774.4030	2320.1872	2320.1508	0.0364	0	18	1.7e+02	1	LTQEELEFMLGEAGAVPTEIK + Oxidation (M)
☑	881	790.1500	2367.4282	2367.2926	0.1356	1	18	1.7e+02	1	HHILCILFFTAHFPVVKLK + 3 Oxidation (HW)
☑	790	595.3760	1783.1062	1782.8505	0.2556	0	18	2.7e+02	1	THLNVTAQCSQMLHK + Oxidation (M)
☑	693	523.2840	1566.8302	1566.7977	0.0325	1	17	2.3e+02	1	LPHPMTPTKFENR
☑	752	567.3010	1698.8812	1698.8507	0.0305	0	17	2.2e+02	1	MICLFLQMAQSTLK + Oxidation (M)
☑	236	428.7270	855.4394	855.4272	0.0122	0	17	2.1e+02	1	EQALHMK
☑	815	613.3760	1837.1062	1837.0111	0.0950	1	17	2.1e+02	1	GFVIPSLYARHLVGHR + Oxidation (HW)
☑	734	552.9120	1655.7142	1655.8227	-0.1085	1	17	2.3e+02	1	QAKRPTEDEVEQAR
☑	523	644.8120	1287.6094	1287.5774	0.0321	0	17	2.6e+02	1	VAMIHMPACDK + Oxidation (M)
☑	567	450.1850	1347.5332	1347.6704	-0.1372	0	17	2.7e+02	1	LQEDMAILQSGK + Oxidation (M)
☑	596	458.5620	1372.6642	1372.6697	-0.0055	1	17	2.6e+02	1	LNYDDFKAIMK + Oxidation (M)
☑	744	559.7090	1676.1052	1676.0600	0.0451	0	17	2.3e+02	1	LVISKPVVSKPVVSKP
☑	767	585.7290	1754.1652	1753.8886	0.2765	0	17	2.3e+02	1	LESLTLGYLDPYQSR
☑	702	531.3200	1590.9382	1590.8519	0.0863	1	17	2.5e+02	1	LVGTFVHKQFGQSK + Oxidation (HW)
☑	746	560.6300	1678.8682	1678.8196	0.0486	1	17	2.5e+02	1	DGDGAITTKELGTVMR + Oxidation (M)
☑	763	579.3580	1735.0522	1734.7454	0.3068	0	17	2.4e+02	1	VNMNEDAFNSMHQAK
☑	66	512.9420	1023.8694	1023.5825	0.2869	1	17	5.4e+02	1	GDSKIHVLR
☑	703	533.0680	1596.1822	1595.7952	0.3870	0	17	2.5e+02	1	LCGLLSMHYLAYR
☑	792	595.4160	1783.2262	1782.9727	0.2535	1	17	3.1e+02	1	TINEAVSPTKIPTSPTK
☑	639	734.1630	1466.3114	1466.7154	-0.4039	0	17	2.7e+02	1	QEALQYEFLNGR
☑	736	833.9460	1665.8774	1665.7880	0.0895	1	17	2.5e+02	1	GKGDDESMTSVSNVLK
☑	708	536.2090	1605.6052	1605.8007	-0.1955	0	17	2.6e+02	1	VLMDQGAFVNPIMR + Oxidation (M)
☑	756	569.6230	1705.8472	1705.8305	0.0167	1	17	2.6e+02	1	IGTDNVRVVAMDGDTK + Oxidation (M)
☑	243	431.1590	860.3034	860.5153	-0.2119	1	17	3.3e+02	1	KISAMLAK
☑	393	531.8110	1061.6074	1061.5506	0.0569	0	17	3.4e+02	1	ISEFPSQVR
☑	811	609.4480	1825.3222	1824.8815	0.4406	0	17	2.4e+02	1	YLVMQGVTDLDDEVTK
☑	819	616.6420	1846.9042	1846.8746	0.0296	0	17	2.5e+02	1	SMYHLFLMIWVTSR + 2 Oxidation (HW); 2 Oxidation (M)
☑	872	744.1440	2229.4102	2229.0661	0.3441	1	17	2.2e+02	1	NEELWAENAQLRSDNEALK
☑	780	589.0570	1764.1492	1763.9166	0.2326	1	17	2.6e+02	1	NAKGLDLTHDALPVER + Oxidation (HW)
☑	843	651.6150	1951.8232	1951.8734	-0.0502	0	17	2.4e+02	1	SLPSVEMEGFQNWQER + Oxidation (M)
☑	469	599.2680	1196.5214	1196.5496	-0.0281	0	17	2.9e+02	1	AALDMDPPPDR
☑	580	683.3770	1364.7394	1364.7160	0.0234	1	17	2.9e+02	1	IQHELQAQRDK
☑	564	673.4440	1344.8734	1344.6746	0.1989	1	17	3e+02	1	QTEPISGNTRSR
☑	638	489.4170	1465.2292	1465.6516	-0.4224	0	17	2.8e+02	1	MWGLPGQSMNMAK + Oxidation (M)
☑	829	620.0770	1857.2092	1857.0162	0.1930	1	17	2.5e+02	1	LVNFIRHHLEPHAFK
☑	705	533.3560	1597.0462	1596.7856	0.2606	1	17	2.7e+02	1	HVDNNKESSLEVAR
☑	655	741.0120	1480.0094	1479.7470	0.2624	0	17	2.8e+02	1	LAINQYGNTFPSR
☑	112	555.3760	1663.1062	1662.8359	0.2703	1	17	6e+02	1	DMVSDNIRTAISVAR + Oxidation (M)
☑	685	517.9550	1550.8432	1550.9144	-0.0712	1	17	2.9e+02	1	VRISNLPLIEEIR
☑	873	744.7590	2231.2552	2231.1005	0.1547	0	17	2.3e+02	1	VSLFMHSVGGSTSPAVPATTNR + Oxidation (M)
☑	797	597.3360	1788.9862	1788.8293	0.1568	1	16	2.8e+02	1	WANYWKNLCIDYK + Oxidation (HW)
☑	486	617.7960	1233.5774	1233.5878	-0.0103	0	16	3.4e+02	1	DFVEGQTVDPK
☑	505	631.6400	1261.2654	1261.6449	-0.3794	1	16	3.5e+02	1	VGRNISEQMTK
☑	574	452.2360	1353.6862	1353.6533	0.0329	0	16	3.2e+02	1	LHICPLHSMAK + 2 Oxidation (HW); Oxidation (M)
☑	400	535.8120	1069.6094	1069.6244	-0.0149	1	16	3.3e+02	1	LQQKIANQK
☑	826	618.3610	1852.0612	1851.8962	0.1649	0	16	2.9e+02	1	QQASHVEAELEEVVER
☑	110	553.5410	1657.6012	1657.8709	-0.2697	1	16	6.9e+02	1	SIKSGDAAPVMLQPTK + Oxidation (M)
☑	267	444.7630	887.5114	887.4899	0.0216	1	16	4.2e+02	1	AQMPVSKK
☑	465	598.3340	1194.6534	1194.6357	0.0178	1	16	3.4e+02	1	RQTAGIFSSTK

☑	671	752.4700	1502.9254	1502.7477	0.1777	1	16	3.4e+02	1	HEGRILDELHGDL
☑	825	927.0190	1852.0234	1851.8673	0.1562	0	16	3e+02	1	AAEVMPDSTLSVGWTTR + Oxidation (HW); Oxidation (M)
☑	704	533.3020	1596.8842	1596.7453	0.1388	0	16	3.2e+02	1	MAEDSYAAQDLVLR + Oxidation (M)
☑	841	647.6980	1940.0722	1939.9058	0.1664	1	16	2.8e+02	1	DLTSWSTSLRGGMTGEAR + Oxidation (M)
☑	99	544.8450	1087.6754	1087.4935	0.1820	0	16	7.4e+02	1	IVDGWDDPR + Oxidation (HW)
☑	554	666.1610	1330.3074	1330.7431	-0.4357	1	16	3.7e+02	1	MPIGRIPSLGFK + Oxidation (M)
☑	531	651.3720	1300.7294	1300.6122	0.1173	0	16	3.6e+02	1	DTTWLYIMAR + Oxidation (HW); Oxidation (M)
☑	836	633.4220	1897.2442	1896.8411	0.4031	0	16	3e+02	1	ETMETSEEQIGIPYDR
☑	802	600.2670	1797.7792	1797.9109	-0.1317	0	16	3e+02	1	LGHLESLIGQPTDSTSK + Oxidation (HW)
☑	215	403.7660	805.5174	805.4004	0.1171	0	16	2.6e+02	1	AVMSGDVK
☑	537	437.2470	1308.7192	1308.5955	0.1237	0	16	3.6e+02	1	WVNCTMEVVR + Oxidation (M)
☑	52	495.4490	1483.3252	1483.6943	-0.3691	0	16	7.9e+02	1	IEYQNEASAANFK
☑	713	541.3080	1620.9022	1620.7492	0.1530	0	16	3.5e+02	1	SRPSSTSTHTSSGWK + Oxidation (HW)
☑	520	426.3000	1275.8782	1275.6684	0.2098	1	16	3.9e+02	1	LHKQHEAAGTGK
☑	835	631.6170	1891.8292	1891.8842	-0.0550	0	16	3.1e+02	1	GMMVSVEIMTDILHER + Oxidation (HW); Oxidation (M)
☑	257	439.2990	876.5834	876.4552	0.1282	0	16	4.3e+02	1	ESSTTKPK
☑	597	458.8410	1373.5012	1373.6800	-0.1789	1	16	3.7e+02	1	TQTYHGTPTRGR
☑	625	475.2780	1422.8122	1422.7177	0.0945	0	16	3.6e+02	1	TNTGGGFMLTIPAK + Oxidation (M)
☑	458	591.3750	1180.7354	1180.5585	0.1770	0	16	3.8e+02	1	SGIENAPNHSR
☑	684	515.3560	1543.0462	1542.6910	0.3552	0	16	3.7e+02	1	DLHTSEDDLAAANR + Oxidation (HW)
☑	33	461.3430	920.6714	920.5080	0.1635	1	16	8.1e+02	1	GFVGRSLVS
☑	676	509.8710	1526.5912	1526.6355	-0.0443	1	16	3.6e+02	1	HGKGGNHCECDLK + Oxidation (HW)
☑	853	662.5820	1984.7242	1985.0153	-0.2911	1	16	3.2e+02	1	YQDKHGMVLIQTGPLNR + Oxidation (M)
☑	242	431.1080	860.2014	860.4174	-0.2160	1	16	4.5e+02	1	MSHGKSSK
☑	353	505.2960	1008.5774	1008.4182	0.1592	0	16	4e+02	1	GDNSVNMEK + Oxidation (M)
☑	837	635.0410	1902.1012	1902.0071	0.0940	1	16	3.3e+02	1	AEALPGNTANQTHVVPKR
☑	401	536.3690	1070.7234	1070.6335	0.0899	1	16	4.1e+02	1	VSEATPKIVK
☑	212	400.7920	799.5694	799.4123	0.1572	1	15	2.6e+02	1	SAKAHMR
☑	525	646.1490	1290.2834	1290.5663	-0.2828	0	15	4.2e+02	1	LWDSGPAAGDMR + Oxidation (HW)
☑	475	606.8090	1211.6034	1211.5683	0.0351	0	15	4e+02	1	GSAAALHWPDR + 2 Oxidation (HW)
☑	785	592.8480	1775.5222	1775.8804	-0.3582	1	15	3.5e+02	1	SFAFEELKEAMAYLK
☑	100	546.3110	1635.9112	1635.6934	0.2178	1	15	8.5e+02	1	EEDDQEGKMEDALK
☑	783	589.6970	1766.0692	1765.9285	0.1407	1	15	3.6e+02	1	MTVEYKLVVVGDGGVGK + Oxidation (M)
☑	184	654.5980	1960.7722	1960.8407	-0.0686	0	15	7.6e+02	1	HAMEQVHENECITFGK + Oxidation (HW); Oxidation (M)
☑	719	542.3000	1623.8782	1623.8113	0.0669	1	15	3.9e+02	1	GSCMGSKYPLINAVK
☑	543	658.9250	1315.8354	1315.6256	0.2099	0	15	4.5e+02	1	DVDLVEDQDLR
☑	380	517.3450	1032.6754	1032.5352	0.1402	0	15	5.1e+02	1	TLNYAAPQR
☑	245	435.2450	868.4754	868.4146	0.0608	1	15	3.4e+02	1	IKSSCMK + Oxidation (M)
☑	718	542.2780	1623.8122	1623.7272	0.0849	0	15	4e+02	1	AQMETEVYMEIHK + Oxidation (M)
☑	553	665.8940	1329.7734	1329.7445	0.0290	0	15	4.6e+02	1	KPIHYEVGFLK
☑	823	618.2540	1851.7402	1851.8044	-0.0642	0	15	3.9e+02	1	LQDMQTSSEETVDPEK + Oxidation (M)
☑	454	587.5820	1173.1494	1173.5626	-0.4131	1	15	5e+02	1	HESAKTTASLN + Oxidation (HW)
☑	75	521.3850	1040.7554	1040.4822	0.2733	0	15	8.8e+02	1	ASPHSGCIGR
☑	805	604.0010	1808.9812	1808.9533	0.0278	1	15	3.9e+02	1	YDIHVVAVAEARIHGK + 2 Oxidation (HW)
☑	753	567.3170	1698.9292	1698.9086	0.0205	1	15	4.1e+02	1	ENLHIATRSLAMLAK + Oxidation (HW); Oxidation (M)
☑	138	587.3610	586.3537	586.3439	0.0099	0	15	1.5	1	GVAGVGK
☑	176	639.9400	1916.7982	1916.8986	-0.1004	0	15	9e+02	1	HVHIGEKPHVCEVCGK + 2 Oxidation (HW)
☑	461	592.9040	1183.7934	1183.5945	0.1989	1	15	4.8e+02	1	TRADNDVHIK + Oxidation (HW)
☑	856	670.1060	2007.2962	2006.9329	0.3633	1	15	3.8e+02	1	TKDNTMPSSTFMEVFLK + 2 Oxidation (M)
☑	177	643.3430	1927.0072	1926.9808	0.0264	1	15	8.8e+02	1	TFLAQCVLRAFQSCVK
☑	528	650.8210	1299.6274	1299.5951	0.0323	1	15	5e+02	1	KTQMMIDDYR
☑	812	611.0530	1830.1372	1829.8652	0.2720	1	15	4.2e+02	1	MAVRSEAVGVTGESMYK + Oxidation (M)
☑	76	521.6600	1561.9582	1561.5629	0.3952	1	15	9.7e+02	1	DEGMMRDEGMMGR + 3 Oxidation (M)
☑	440	572.7870	1143.5594	1143.6499	-0.0905	0	15	5.4e+02	1	LDVIITSQAGK
☑	68	513.9750	1538.9032	1538.7100	0.1932	1	14	9.6e+02	1	KEQEEEYGTVEAK
☑	437	568.3120	1134.6094	1134.5571	0.0524	0	14	5.7e+02	1	GHWHGDVAIK + Oxidation (HW)
☑	618	705.9270	1409.8394	1409.7011	0.1383	1	14	4.8e+02	1	YSVGGASSRGVASGR
☑	804	603.6520	1807.9342	1807.8682	0.0660	1	14	4.3e+02	1	WKYAEIPEHYVWR + 2 Oxidation (HW)
☑	502	631.5970	1261.1794	1261.5608	-0.3814	0	14	5.4e+02	1	EEELLGCEAGR
☑	468	598.8620	1195.7094	1195.5622	0.1473	0	14	5e+02	1	ELWTAYQNR + Oxidation (HW)
☑	558	446.2520	1335.7342	1335.6163	0.1179	0	14	5.1e+02	1	SVMVSQALGVGCE
☑	172	635.9340	1904.7802	1905.0948	-0.3147	1	14	9.5e+02	1	GPRPIPGFGNLKDVLLGR
☑	866	684.4970	2050.4692	2050.9477	-0.4786	0	14	4e+02	1	GASSDVSDNSLVECNTVLGK
☑	98	544.3080	1629.9022	1629.7780	0.1241	0	14	1.1e+03	1	NLSMVNHSTNLQEK + Oxidation (M)
☑	137	586.9190	1757.7352	1757.7349	0.0003	1	14	1e+03	1	CGKAMDDSGDVGEYVR
☑	869	694.6110	2080.8112	2080.9888	-0.1776	0	14	4.1e+02	1	FDDLPSVNLGFMIPGSSNR + Oxidation (M)
☑	876	752.4220	2254.2442	2254.1311	0.1131	1	14	3.9e+02	1	LHGQTALMLAVSHGRIDMTR + 2 Oxidation (HW); Oxidation (M)
☑	513	636.8780	1271.7414	1271.6469	0.0945	0	14	5.5e+02	1	QQLSAQLEEAR
☑	190	666.4710	1330.9274	1330.7608	0.1666	0	14	1e+03	1	IAPSAYSALILGR
☑	101	547.3570	1639.0492	1638.9127	0.1365	1	14	1.1e+03	1	LALKGVVEAVEPMQR

☑	157	605.7210	1209.4274	1209.6652	-0.2377	0	14	1e+03	1	MHAASRPILAK + Oxidation (HW)
☑	648	491.9860	1472.9362	1472.7722	0.1639	0	14	5.3e+02	1	TLTPELSSAVVDNK
☑	488	414.2530	1239.7372	1239.6320	0.1052	1	14	5.4e+02	1	QSVLEEARHR + Oxidation (HW)
☑	569	674.8600	1347.7054	1347.7254	-0.0200	1	14	5.8e+02	1	IKIQNCIMSLL + Oxidation (M)
☑	192	672.5490	671.5417	671.4078	0.1339	1	14	18	1	KAAIGGR
☑	822	617.8510	1850.5312	1850.0625	0.4687	1	14	4.6e+02	1	TKPPKTSIAPLTQNNIK
☑	697	527.3030	1578.8872	1578.8478	0.0394	1	14	5.2e+02	1	QEIVHKIQIQDGR + Oxidation (HW)
☑	759	573.0450	1716.1132	1715.8730	0.2402	0	14	5e+02	1	NDPGASLIPFSGALETK
☑	432	565.4990	1128.9834	1128.5312	0.4523	1	14	6.1e+02	1	AGYKGSNNYR
☑	591	686.4130	1370.8114	1370.7518	0.0597	1	14	5.7e+02	1	DRTVTAGNPVTLK
☑	407	541.5750	1081.1354	1081.6244	-0.4889	1	14	5.7e+02	1	KAEPGTKPVR
☑	133	573.2750	1144.5354	1144.5612	-0.0257	0	14	1.1e+03	1	KPTETTDPEK
☑	11	424.3660	1270.0762	1269.7445	0.3317	0	14	1.2e+03	1	VLFPTTVALGPR
☑	821	617.0140	1848.0202	1847.7463	0.2738	0	14	4.9e+02	1	GIDQGDMMMHFYTMR + Oxidation (M)
☑	62	506.4680	1010.9214	1010.5470	0.3744	1	14	1.1e+03	1	MFEKISLK + Oxidation (M)
☑	128	566.3010	1695.8812	1695.9705	-0.0894	1	14	1.2e+03	1	NPNTKSVAIKPVLMGK
☑	23	450.2280	898.4414	898.4872	-0.0458	0	14	1.2e+03	1	IETVPGQR
☑	403	538.4370	1074.8594	1074.5611	0.2984	0	14	7e+02	1	LSAGPAWFAR
☑	423	557.0700	1112.1254	1111.6349	0.4905	1	14	6e+02	1	EGKLLPVNSR
☑	424	557.4420	1112.8694	1112.6302	0.2393	0	14	6.2e+02	1	ILNSKPQASR
☑	760	573.3630	1717.0672	1716.9159	0.1513	0	13	5.6e+02	1	LNDHEIDIVLGQVPR
☑	695	523.3200	1566.9382	1566.6951	0.2431	0	13	5.9e+02	1	NSWGTTWGDEGTIK + Oxidation (HW)
☑	367	508.8390	1015.6634	1015.5411	0.1224	0	13	7.5e+02	1	AGITVTAGGNR
☑	241	431.0920	860.1694	860.4756	-0.3062	0	13	7.5e+02	1	NIPSFGVK
☑	336	492.7800	983.5454	983.4594	0.0861	1	13	6.5e+02	1	TTSGTDKMK + Oxidation (M)
☑	443	575.8140	1149.6134	1149.5641	0.0494	1	13	6.9e+02	1	YKHPYAMPK + Oxidation (M)
☑	127	565.6660	1693.9762	1693.8127	0.1635	1	13	1.3e+03	1	NVLGTKNMAEMLSDR + Oxidation (M)
☑	314	481.3310	960.6474	960.4739	0.1736	0	13	8.2e+02	1	DWIVMLAN
☑	539	656.2360	1310.4574	1310.8286	-0.3711	1	13	6.4e+02	1	GLQLVKVSLLNK
☑	271	447.2440	892.4734	892.3886	0.0848	0	13	7e+02	1	LSDSGSGDR
☑	765	580.3100	1737.9082	1737.7087	0.1995	1	13	5.8e+02	1	VEGFECCHKNSDEK
☑	123	563.8230	1688.4472	1688.8733	-0.4262	0	13	1.3e+03	1	SPPLPQPLIDSSSHAK + Oxidation (HW)
☑	694	523.3140	1566.9202	1566.7348	0.1854	1	13	6.2e+02	1	SLTKEEQHSSFMK + Oxidation (M)
☑	155	601.8550	1802.5432	1802.8906	-0.3475	0	13	1.3e+03	1	VELAEMGPSLNLEVMR + Oxidation (M)
☑	334	492.5380	983.0614	983.5400	-0.4785	0	13	6.4e+02	1	QLAEIASPR
☑	576	455.2080	1362.6022	1362.6892	-0.0870	0	13	6.8e+02	1	FVDNVSVISNNR
☑	381	517.8230	1033.6314	1033.4750	0.1565	0	13	8.2e+02	1	ISEMPNAEK + Oxidation (M)
☑	522	643.3920	1284.7694	1284.5955	0.1740	0	13	6.9e+02	1	MNFTATMVQAR + Oxidation (M)
☑	592	458.5530	1372.6372	1372.6227	0.0144	1	13	7e+02	1	QTRYGMDAMLR + 2 Oxidation (M)
☑	453	586.9850	1171.9554	1171.4672	0.4883	0	13	7.3e+02	1	TCSNSMTPMK + Oxidation (M)
☑	200	694.3770	693.3697	693.3479	0.0218	1	13	16	1	SSMGKGK
☑	859	673.4720	2017.3942	2016.9939	0.4003	1	13	5.8e+02	1	LENTQSILKIGDHINCF + Oxidation (HW)
☑	208	790.1750	789.1677	789.3327	-0.1650	0	13	5e+02	1	VASEMHT + Oxidation (HW)
☑	7	413.3480	1237.0222	1236.7190	0.3032	1	13	1.2e+03	1	KKPDPEIIAAR
☑	244	434.8680	867.7214	867.3943	0.3272	0	13	6.4e+02	1	CSTTIMR
☑	139	587.7210	1760.1412	1759.7319	0.4093	0	13	1.4e+03	1	DDNNAITTGPEHADMK + Oxidation (HW); Oxidation (M)
☑	833	629.3480	1885.0222	1884.9767	0.0455	1	13	5.9e+02	1	NLYNSEHVAIKLEPMK
☑	536	655.3170	1308.6194	1308.7077	-0.0883	1	13	7.2e+02	1	AADKVPYYAALK
☑	316	487.3000	972.5854	972.4777	0.1077	0	13	8.7e+02	1	ESFLHAGGR
☑	182	649.4760	1945.4062	1944.9952	0.4110	1	13	1.4e+03	1	DPAVIPAGHHTTSISRMR
☑	758	572.4000	1714.1782	1713.7954	0.3828	0	13	6.6e+02	1	LMQVISQEIDTQMF + 2 Oxidation (M)
☑	585	456.9030	1367.6872	1367.6643	0.0229	1	13	7.2e+02	1	VDFEKEDLVMK + Oxidation (M)
☑	631	484.5780	1450.7122	1450.7317	-0.0195	0	13	7.2e+02	1	NDTQPVWIGLHR + Oxidation (HW)
☑	129	567.3420	1699.0042	1698.9742	0.0299	0	13	1.5e+03	1	FVPPSTTMKPKPLLK + Oxidation (M)
☑	842	651.4910	1951.4512	1951.9092	-0.4580	1	13	6.3e+02	1	STSSTAHRIMVDDIMTR + 2 Oxidation (M)
☑	654	493.8670	1478.5792	1478.8028	-0.2236	1	13	7.5e+02	1	TLPVCHKGLGLER
☑	227	421.2010	840.3874	840.4276	-0.0401	0	13	5.3e+02	1	IHVNCAK
☑	122	563.2640	1124.5134	1124.5760	-0.0626	1	13	1.5e+03	1	IHKEGCPLR + Oxidation (HW)
☑	24	450.6270	1348.8592	1348.6219	0.2373	1	13	1.7e+03	1	EASRTEGAISTNN
☑	130	567.8970	1700.6692	1700.9825	-0.3133	0	13	1.6e+03	1	LNIIVVAFDLSGTLAR
☑	650	492.3100	1473.9082	1473.7174	0.1908	1	13	7.7e+02	1	KFVEAVMDYTGAK + Oxidation (M)
☑	221	412.7510	823.4874	823.4109	0.0765	0	12	4.9e+02	1	VATSATMK + Oxidation (M)
☑	533	436.9170	1307.7292	1307.6391	0.0901	0	12	7.8e+02	1	MVSNSITTSPAGK + Oxidation (M)
☑	782	589.4180	1765.2322	1764.8683	0.3639	1	12	7e+02	1	EIHSYKVYDLTTHK + 2 Oxidation (HW)
☑	290	465.8080	929.6014	929.5620	0.0395	1	12	9.8e+02	1	IPMIKLIS + Oxidation (M)
☑	78	524.6730	1047.3314	1047.4291	-0.0977	0	12	1.7e+03	1	HDTMGPSGSK + Oxidation (HW); Oxidation (M)
☑	636	729.3030	1456.5914	1456.7383	-0.1468	1	12	7.8e+02	1	THETGSVVATQRR + Oxidation (HW)
☑	699	529.9580	1586.8522	1586.7518	0.1004	1	12	7.6e+02	1	HLDFFRFGEEYK
☑	861	674.5290	2020.5652	2021.0139	-0.4488	0	12	6.5e+02	1	ALMESDQFLTVEPTGLVR + Oxidation (M)
☑	327	490.3030	978.5914	978.4916	0.0998	1	12	9.2e+02	1	TMSAELRR + Oxidation (M)

☑	335	492.5410	983.0674	983.4205	-0.3530	0	12	7.9e+02	1	MTNTGWMK + Oxidation (M)
☑	706	533.3810	1597.1212	1596.7644	0.3567	1	12	7.6e+02	1	SHISETHKYAHQK + 2 Oxidation (HW)
☑	847	657.0750	1968.2032	1967.9847	0.2185	0	12	6.6e+02	1	ILQEHSGQIGIMLNNNR + Oxidation (HW); Oxidation (M)
☑	563	447.1630	1338.4672	1338.6503	-0.1831	1	12	8.2e+02	1	KHFSYQGMIGR + Oxidation (M)
☑	113	555.9250	1109.8354	1109.4659	0.3696	1	12	1.6e+03	1	DMETESRDK
☑	855	668.4430	2002.3072	2001.8462	0.4610	0	12	7e+02	1	DCWYLYTLQGNHMNR + Oxidation (HW); Oxidation (M)
☑	879	789.4910	2365.4512	2365.1923	0.2589	1	12	6.2e+02	1	MVTVNPRVFFDIEVGGLPMGR + 2 Oxidation (M)
☑	80	530.5500	1588.6282	1588.6353	-0.0072	1	12	1.9e+03	1	MMDRMEAMNGDLK + 3 Oxidation (M)
☑	524	646.0400	1290.0654	1289.6629	0.4025	0	12	9.3e+02	1	GHGPWLSVIHR + 2 Oxidation (HW)
☑	441	573.8600	1145.7054	1145.6267	0.0787	0	12	1e+03	1	VIPGFMIQGGK
☑	779	880.5460	1759.0774	1758.8247	0.2528	0	12	7.9e+02	1	MESAGVHQVVDIEWK + Oxidation (HW); Oxidation (M)
☑	341	496.4930	990.9714	991.3777	-0.4063	0	12	1.1e+03	1	NECNDQGR
☑	769	586.3220	1755.9442	1755.7965	0.1477	0	12	7.8e+02	1	HHQSNHFTLAASVPY + 3 Oxidation (HW)
☑	499	630.3310	1258.6474	1258.7649	-0.1174	0	12	9.4e+02	1	LQGALQILLYK
☑	500	630.4460	1258.8774	1258.6088	0.2686	0	12	9.4e+02	1	AQGQLLQECGR
☑	672	502.2970	1503.8692	1503.7794	0.0898	1	12	8.6e+02	1	LSAQDERVAAFAAR
☑	686	519.3400	1554.9982	1554.7022	0.2959	1	12	8.6e+02	1	KGENTDDIEANHGR
☑	766	585.6040	1753.7902	1753.7618	0.0284	0	12	8.1e+02	1	ETFDTVDNWSMHLK + Oxidation (HW); Oxidation (M)
☑	210	795.2660	794.2587	794.3116	-0.0529	0	12	3.8e+02	1	DEAAMDK + Oxidation (M)
☑	347	498.6390	995.2634	995.4672	-0.2037	0	12	9e+02	1	YNDASSALR
☑	809	608.4940	1822.4602	1822.8018	-0.3417	0	12	7.7e+02	1	TVPEVMWDYVEHMR + 2 Oxidation (M)
☑	194	677.0240	676.0167	676.3504	-0.3337	0	12	58	1	GVTGTSR
☑	582	456.2570	1365.7492	1365.7405	0.0087	0	12	9e+02	1	IGHLISQWLQGV + Oxidation (HW)
☑	839	639.9150	1916.7232	1916.8646	-0.1415	1	12	7.7e+02	1	NMSARVVDNDNPLADDR + Oxidation (M)
☑	724	547.3410	1639.0012	1638.7849	0.2162	0	12	8.4e+02	1	SLDLLGDDDLAHPSR + Oxidation (HW)
☑	526	647.4850	1292.9554	1292.5481	0.4074	0	12	9.5e+02	1	QGTSTETDHSSK + Oxidation (HW)
☑	673	503.3490	1507.0252	1506.6847	0.3405	1	12	9.1e+02	1	TSDFSMLREMFK + Oxidation (M)
☑	397	533.3480	1064.6814	1064.6091	0.0724	1	12	1e+03	1	QHVPISTKR
☑	141	589.2210	1764.6412	1764.9021	-0.2610	0	12	1.9e+03	1	ILHQWAMVNAFLFK + 2 Oxidation (HW); Oxidation (M)
☑	295	468.7750	935.5354	935.5011	0.0344	0	12	1e+03	1	SHMLAIHK
☑	189	664.6810	1991.0212	1990.9571	0.0641	1	12	1.8e+03	1	LTLHVGDGFEFMKNHSK + Oxidation (HW); Oxidation (M)
☑	259	440.4920	878.9694	878.5338	0.4357	1	12	9.3e+02	1	SPVHIAKK
☑	346	498.3670	994.7194	994.4542	0.2652	0	12	1e+03	1	QHLGSMYK + Oxidation (HW); Oxidation (M)
☑	503	631.6110	1261.2074	1261.5496	-0.3422	0	12	1.1e+03	1	ECTEVVDPGEK
☑	90	539.3490	1615.0252	1614.7672	0.2580	1	12	2e+03	1	SSFGEGLTSTQAMRK + Oxidation (M)
☑	43	480.5080	1438.5022	1438.6735	-0.1714	1	11	2.3e+03	1	CHTHLGNTEKSR
☑	629	479.2390	1434.6952	1434.6847	0.0105	1	11	1e+03	1	MAEETLVGPCGKK + Oxidation (M)
☑	557	666.4310	1330.8474	1330.6816	0.1659	1	11	1.1e+03	1	MPIGRAWEALR + Oxidation (HW); Oxidation (M)
☑	831	623.4090	1867.2052	1866.8856	0.3196	0	11	8.6e+02	1	IMTAVMNAETVGSTWPK + 2 Oxidation (M)
☑	840	646.7130	1937.1172	1936.9387	0.1785	0	11	8.8e+02	1	LFIGVMNSLLEMEHTR + Oxidation (HW); 2 Oxidation (M)
☑	698	527.6100	1579.8082	1579.7842	0.0240	1	11	9.5e+02	1	NATTKSILDYDSPR
☑	260	440.7280	879.4414	879.3868	0.0546	1	11	1.1e+03	1	GESERMR + Oxidation (M)
☑	720	543.3030	1626.8872	1626.8114	0.0758	1	11	9.6e+02	1	QKSSHETGAIWEVR
☑	368	510.3210	1018.6274	1018.5196	0.1079	1	11	1.3e+03	1	KAAWSDVAR + Oxidation (HW)
☑	296	469.2740	936.5334	936.4447	0.0888	1	11	1.1e+03	1	MSKASQNR + Oxidation (M)
☑	581	455.9290	1364.7652	1364.7524	0.0127	1	11	1.1e+03	1	AVAKIHLGQQER + Oxidation (HW)
☑	348	500.6660	999.3174	999.4695	-0.1521	0	11	1.2e+03	1	VTFMSNASK + Oxidation (M)
☑	360	506.3310	1010.6474	1010.4702	0.1772	1	11	1.1e+03	1	KATSSMADGK + Oxidation (M)
☑	521	639.3540	1276.6934	1276.6486	0.0449	0	11	1.2e+03	1	TAFLVVTHDMK + Oxidation (M)
☑	124	564.2700	1689.7882	1689.8356	-0.0474	0	11	2.2e+03	1	SIVSCLDVNNSLQNK
☑	544	659.7310	1317.4474	1317.6499	-0.2025	0	11	1.2e+03	1	LYGHMAAANVQK + Oxidation (M)
☑	530	651.3520	1300.6894	1300.7061	-0.0166	0	11	1.2e+03	1	ESCQLPTLLLK
☑	751	565.3510	1693.0312	1692.8063	0.2249	0	11	1e+03	1	DIGTMAMLLDAPSGTGK + Oxidation (M)
☑	764	580.0680	1737.1822	1736.7961	0.3861	0	11	1e+03	1	MEEVVTGLQIDDGGMK + Oxidation (M)
☑	19	440.2250	878.4354	878.4718	-0.0363	1	11	2.4e+03	1	VVMGMKSK
☑	371	512.2860	1022.5574	1022.4703	0.0872	0	11	1.2e+03	1	TSMGDQIGSK
☑	300	472.7760	943.5374	943.4723	0.0651	0	11	1.5e+03	1	GTQVGNSPGK
☑	535	655.2860	1308.5574	1308.6132	-0.0558	0	11	1.2e+03	1	LSSSFDLTMHR + Oxidation (M)
☑	511	635.6260	1269.2374	1269.7153	-0.4779	1	11	1.2e+03	1	IQQGSGLGNLKR
☑	862	676.5660	2026.6762	2026.9418	-0.2657	0	11	9.6e+02	1	LSDLAQWTSGWLMTSGSR + Oxidation (HW); Oxidation (M)
☑	740	558.0520	1671.1342	1670.8628	0.2714	0	11	1.1e+03	1	ADSSQFTIPAASVHIK
☑	473	602.8550	1203.6954	1203.5163	0.1791	1	11	1.3e+03	1	SNGGSASCRHR + Oxidation (HW)
☑	223	414.1510	826.2874	826.3504	-0.0630	0	10	7.6e+02	1	GSAHHMR + Oxidation (HW); Oxidation (M)
☑	88	536.5900	1071.1654	1071.5131	-0.3477	1	10	2.6e+03	1	GGRNQEMALP
☑	450	585.2720	1168.5294	1168.6162	-0.0867	1	10	1.3e+03	1	GVSLKMYLDK + Oxidation (M)
☑	865	683.9270	2048.7592	2049.0683	-0.3092	1	10	1e+03	1	IRAEHLLDGYDFNFIVK
☑	692	523.2370	1566.6892	1566.6668	0.0224	0	10	1.2e+03	1	HLMHDNVHGACTK + 2 Oxidation (HW); Oxidation (M)
☑	733	551.0170	1650.0292	1649.7251	0.3040	1	10	1.2e+03	1	FMMSYQKNDMIDK
☑	611	703.4450	1404.8754	1404.6779	0.1975	1	10	1.3e+03	1	EQMPSISSQSRR
☑	527	650.6570	1299.2994	1299.6129	-0.3134	1	10	1.3e+03	1	TQEGKSMNYVK + Oxidation (M)

☑	810	609.3870	1825.1392	1824.8714	0.2677	1	10	1.1e+03	1	HSGNPSNKSQVEEQLR + Oxidation (HW)
☑	355	505.6950	1009.3754	1009.4386	-0.0632	0	10	1.3e+03	1	DGLSADSMSK
☑	816	615.3630	1843.0672	1842.8723	0.1949	1	10	1.1e+03	1	LADFGFARYMIDSHGK + Oxidation (HW)
☑	205	731.5610	730.5537	730.3246	0.2292	0	10	4e+02	1	EAGPSDR
☑	555	666.1690	1330.3234	1330.6226	-0.2991	1	10	1.4e+03	1	GNQQSGAGGGAGKDK
☑	431	563.8110	1125.6074	1125.4608	0.1466	0	10	1.4e+03	1	TSDDGLSMER + Oxidation (M)
☑	577	682.3290	1362.6434	1362.6999	-0.0565	0	10	1.4e+03	1	INLMTEMVAGIR + Oxidation (M)
☑	331	491.2170	980.4194	980.5403	-0.1209	0	10	1.4e+03	1	ALSLDIHGR
☑	389	528.5380	1055.0614	1055.4533	-0.3918	0	10	1.4e+03	1	WQAHNDNR + Oxidation (HW)
☑	312	480.0090	958.0034	958.4364	-0.4330	1	10	1.6e+03	1	GYAMSMRK + Oxidation (M)
☑	277	455.1340	908.2534	908.4577	-0.2042	1	10	1.4e+03	1	HTGEGPRR
☑	439	572.6190	1143.2234	1143.5607	-0.3373	1	10	1.6e+03	1	SCWPGRLPR + Oxidation (HW)
☑	388	527.3400	1052.6654	1052.4808	0.1847	0	10	1.4e+03	1	SNASSAMLEK + Oxidation (M)
☑	675	508.3860	1522.1362	1521.7722	0.3640	1	10	1.4e+03	1	MASRGGFLVGDGALR + Oxidation (M)
☑	164	621.5240	1861.5502	1861.9608	-0.4106	0	10	2.7e+03	1	NALFGSSLLLANSPMSPK + Oxidation (M)
☑	832	625.4060	1873.1962	1872.9363	0.2598	1	10	1.3e+03	1	GPVDVRNALSIMGAENSK + Oxidation (M)
☑	217	404.8290	807.6434	807.3909	0.2526	0	10	8.8e+02	1	IMGTQSR + Oxidation (M)
☑	632	726.3840	1450.7534	1450.7204	0.0330	1	10	1.5e+03	1	LQIARDEYYHK + Oxidation (HW)
☑	298	472.2760	942.5374	942.4229	0.1145	1	10	1.7e+03	1	GKDMHDPK + Oxidation (M)
☑	373	513.2760	1024.5374	1024.4309	0.1066	0	10	1.6e+03	1	DASESTSNSK
☑	602	690.3800	1378.7454	1378.6412	0.1043	1	10	1.5e+03	1	TWLRSNMGQDR + Oxidation (HW)
☑	538	655.6150	1309.2154	1309.6514	-0.4359	0	10	1.5e+03	1	SEPSISSIAYTR
☑	849	661.5600	1981.6582	1981.9262	-0.2681	1	9	1.3e+03	1	KEPMTGVETGSNSASGISSK + Oxidation (M)
☑	234	427.3520	852.6894	852.5181	0.1713	0	9	1.1e+03	1	VVVNVAPR
☑	289	464.2460	926.4774	926.4821	-0.0047	0	9	1.6e+03	1	SLSGELGHK
☑	235	428.6950	855.3754	855.4814	-0.1060	0	9	1.4e+03	1	LTNVAPNK
☑	256	439.2630	876.5114	876.4739	0.0376	1	9	1.9e+03	1	KELMNVK + Oxidation (M)
☑	463	596.6000	1191.1854	1191.5255	-0.3400	0	9	1.8e+03	1	ETIENSNEEK
☑	343	497.2890	992.5634	992.4597	0.1037	0	9	1.9e+03	1	DIDGLGTMR + Oxidation (M)
☑	451	586.3290	1170.6434	1170.5816	0.0619	0	9	1.7e+03	1	SAQVGCGIVHK + Oxidation (HW)
☑	504	631.6190	1261.2234	1261.6640	-0.4405	1	9	1.8e+03	1	QHSLQQPPRR + Oxidation (HW)
☑	748	563.8340	1688.4802	1688.7828	-0.3026	1	9	1.6e+03	1	DHIDSLIRDMEWK + Oxidation (HW); Oxidation (M)
☑	429	562.6770	1123.3394	1123.5947	-0.2552	0	9	1.7e+03	1	LTNLMYEIK
☑	197	689.4110	688.4037	688.4119	-0.0082	0	9	2.2e+02	1	ASTIGIK
☑	330	491.1860	980.3574	980.4675	-0.1101	0	9	1.8e+03	1	THLESHNK + Oxidation (HW)
☑	275	452.2740	902.5334	902.4974	0.0360	0	9	2.2e+03	1	GPGVASLFR
☑	2	402.7430	1205.2072	1205.6703	-0.4631	1	9	3.4e+03	1	MNFTRAVLVR
☑	598	688.1310	1374.2474	1374.5908	-0.3433	1	9	1.8e+03	1	TYTMMGNKEER + Oxidation (M)
☑	219	405.2510	808.4874	808.4331	0.0544	0	9	8.9e+02	1	SGAVLISY
☑	559	446.9030	1337.6872	1337.6432	0.0440	0	9	1.7e+03	1	MTTTAGVGGAGMLR + Oxidation (M)
☑	425	557.6550	1113.2954	1113.5567	-0.2613	0	9	1.9e+03	1	DGSHILPFGR + Oxidation (HW)
☑	477	608.4330	1214.8514	1214.6619	0.1896	1	9	2e+03	1	QLKTSTSHSVK
☑	342	497.2640	992.5134	992.4597	0.0538	1	9	2.1e+03	1	AENKADAMK + Oxidation (M)
☑	562	669.9090	1337.8034	1337.6285	0.1749	0	9	1.8e+03	1	MAPLSEANQSFK + Oxidation (M)
☑	455	590.3410	1178.6674	1178.5754	0.0921	0	9	2e+03	1	WLNLQTMNK + Oxidation (HW); Oxidation (M)
☑	682	514.3630	1540.0672	1539.6974	0.3697	0	9	1.8e+03	1	STSMSVDVDVDIEK + Oxidation (M)
☑	395	533.2610	1064.5074	1064.4920	0.0154	1	9	2e+03	1	LKNTNASASC
☑	248	435.7950	869.5754	869.4971	0.0784	0	9	1.8e+03	1	VVSLTHAK + Oxidation (HW)
☑	258	439.9870	877.9594	877.4765	0.4829	1	9	2.1e+03	1	MMIKLNI + Oxidation (M)
☑	446	581.2860	1160.5574	1160.5244	0.0330	0	9	2.1e+03	1	GLVAQEDCNR
☑	605	694.3710	1386.7274	1386.6991	0.0284	0	9	2e+03	1	DVNEVENITNIK
☑	268	444.8720	887.7294	887.4613	0.2681	1	9	2.4e+03	1	HAVKDYR
☑	480	613.7000	1225.3854	1225.6013	-0.2158	0	8	2e+03	1	GLATMAAEAFTK + Oxidation (M)
☑	224	416.4820	830.9494	830.4861	0.4633	1	8	2.6e+03	1	KEGLSLGK
☑	333	492.4890	982.9634	982.5196	0.4439	1	8	2.1e+03	1	DRVHTIDK
☑	445	580.0220	1158.0294	1158.5088	-0.4793	1	8	2.4e+03	1	KIDCSTANNH
☑	195	678.3140	677.3067	677.3344	-0.0277	0	8	60	1	TATGGGSK
☑	183	653.3420	652.3347	652.3657	-0.0309	0	8	15	1	GVPAGPR
☑	419	555.0620	1108.1094	1108.5256	-0.4162	0	8	2.3e+03	1	MIMNTENIK + Oxidation (M)
☑	278	455.2790	908.5434	908.5192	0.0243	1	8	2.4e+03	1	QKIGPNPR
☑	286	462.2610	922.5074	922.5097	-0.0022	1	8	2.6e+03	1	HSASIPRR
☑	246	435.4630	868.9114	869.3259	-0.4144	0	8	1.9e+03	1	MMDSENK + Oxidation (M)
☑	269	446.8270	891.6394	891.4735	0.1659	0	7	3.1e+03	1	SLMISPTK + Oxidation (M)
☑	478	610.0280	1218.0414	1217.6252	0.4163	0	7	3e+03	1	TENLSSELAVR
☑	211	400.7450	799.4754	799.4374	0.0380	0	7	1.8e+03	1	AAPLCIR
☑	610	703.3450	1404.6754	1404.7613	-0.0858	1	7	3e+03	1	STDSKLSWLIQK
☑	213	401.1720	800.3294	800.4028	-0.0734	0	7	2.2e+03	1	EELGTPR
☑	203	699.0500	698.0427	698.3963	-0.3536	0	6	2.2e+02	1	SPGPTLK
☑	302	473.2980	944.5814	944.4563	0.1251	0	6	4.1e+03	1	HESSQITK + Oxidation (HW)
☑	386	523.3240	1044.6334	1044.4368	0.1966	0	6	3.7e+03	1	EVMCTAYR + Oxidation (M)

☑	270	446.9850	891.9554	892.4073	-0.4518	1	6	3.9e+03	1	KQQSDCK
☑	394	532.8990	1063.7834	1063.4653	0.3181	0	6	4e+03	1	GLFMMMYR + Oxidation (M)
☑	362	508.5720	1015.1294	1015.5410	-0.4116	0	6	4.4e+03	1	INLQQSASR
☑	171	634.2600	633.2527	633.2792	-0.0265	0	5	34	1	SGVSGMP
☑	291	466.2960	930.5774	930.4519	0.1256	1	5	5.1e+03	1	KNTHSSNK + Oxidation (HW)
☑	354	505.4450	1008.8754	1008.4798	0.3957	0	5	4.4e+03	1	ICSTISLTD
☑	476	606.8630	1211.7114	1211.5712	0.1402	0	5	4.3e+03	1	MDMTTLTIMR
☑	198	691.3950	690.3877	690.3371	0.0507	0	5	4.3e+02	1	IGAMDGK
☑	206	743.4450	742.4377	742.3762	0.0615	0	4	1.6e+03	1	TPGGFHK
☑	202	698.4300	697.4227	697.3620	0.0608	1	3	1.7e+02	1	GSRGGHK
☑	181	648.3720	647.3647	647.2875	0.0772	0	3	1.4e+02	1	GGSGDGAK
☑	140	588.4730	587.4657	587.3391	0.1266	1	2	25	1	AGGGAKK
☑	169	627.5060	626.4987	626.3136	0.1851	0	2	33	1	GTGGAHK
☑	95	543.4270	542.4197	542.2813	0.1385	0	2	0.85	1	AGGPGGK
☑	143	591.4590	590.4517	590.2660	0.1857	0	1	52	1	GSAGGDK
☑	187	661.4970	660.4897	660.3555	0.1342	1	1	5.8e+02	1	KAGGGGSK
☑	174	639.3220	638.3147	638.3500	-0.0353	0	1	33	1	GGGPPVR
☑	5	*409.4100*	*408.4027*							
☑	6	*409.4100*	*408.4027*							
☑	8	*414.1950*	*413.1877*							
☑	9	*423.4110*	*422.4037*							
☑	10	*423.4240*	*422.4167*							
☑	12	*429.8000*	*428.7927*							
☑	13	*432.2500*	*431.2427*							
☑	14	*437.3190*	*436.3117*							
☑	15	*437.4420*	*436.4347*							
☑	16	*438.2440*	*437.2367*							
☑	17	*439.3730*	*438.3657*							
☑	18	*440.1820*	*439.1747*							
☑	20	*441.2610*	*440.2537*							
☑	21	*444.1680*	*443.1607*							
☑	22	*445.2180*	*444.2107*							
☑	25	*451.4040*	*450.3967*							
☑	26	*451.4110*	*450.4037*							
☑	27	*451.4420*	*450.4347*							
☑	28	*453.3300*	*452.3227*							
☑	29	*457.4550*	*456.4477*							
☑	30	*458.1650*	*457.1577*							
☑	31	*458.3170*	*457.3097*							
☑	32	*459.0970*	*458.0897*							
☑	34	*463.0830*	*462.0757*							
☑	35	*463.3300*	*462.3227*							
☑	36	*465.3350*	*464.3277*							
☑	38	*474.2700*	*473.2627*							
☑	39	*474.3890*	*473.3817*							
☑	40	*475.3730*	*474.3657*							
☑	41	*476.1400*	*475.1327*							
☑	42	*480.2910*	*479.2837*							
☑	44	*481.3850*	*480.3777*							
☑	45	*484.3610*	*483.3537*							
☑	46	*487.2270*	*486.2197*							
☑	47	*488.4250*	*487.4177*							
☑	48	*489.4520*	*488.4447*							
☑	49	*492.6120*	*491.6047*							
☑	50	*494.1680*	*493.1607*							
☑	51	*495.2930*	*494.2857*							
☑	53	*496.2480*	*495.2407*							
☑	54	*499.2360*	*498.2287*							
☑	55	*499.9710*	*498.9637*							
☑	56	*503.4600*	*502.4527*							
☑	57	*504.0340*	*503.0267*							
☑	58	*504.2720*	*503.2647*							
☑	59	*505.3390*	*504.3317*							
☑	60	*505.4310*	*504.4237*							
☑	63	*507.0880*	*506.0807*							
☑	64	*508.3610*	*507.3537*							
☑	65	*512.6120*	*511.6047*							
☑	67	*513.2280*	*512.2207*							
☑	69	*515.2510*	*514.2437*							
☑	70	*515.4810*	*514.4737*							

☑	71	516.4780	515.4707
☑	73	520.2610	519.2537
☑	79	527.4830	526.4757
☑	82	531.5220	530.5147
☑	85	533.5900	532.5827
☑	86	535.3780	534.3707
☑	87	536.0480	535.0407
☑	89	537.9810	536.9737
☑	91	540.7120	539.7047
☑	92	541.3800	540.3727
☑	93	542.8040	541.7967
☑	96	543.5110	542.5037
☑	102	547.4710	546.4637
☑	103	549.3080	548.3007
☑	106	551.1640	550.1567
☑	107	551.3170	550.3097
☑	108	551.5500	550.5427
☑	109	553.4110	552.4037
☑	111	554.3880	553.3807
☑	114	557.7170	556.7097
☑	115	558.2240	557.2167
☑	116	558.4050	557.3977
☑	117	558.5570	557.5497
☑	118	559.4810	558.4737
☑	119	560.3420	559.3347
☑	120	561.4800	560.4727
☑	121	562.6190	561.6117
☑	125	565.4440	564.4367
☑	126	565.5150	564.5077
☑	131	568.6070	567.5997
☑	132	571.9010	570.8937
☑	134	575.4560	574.4487
☑	136	584.8470	583.8397
☑	142	589.3180	588.3107
☑	144	591.5250	590.5177
☑	145	592.1060	591.0987
☑	146	593.5460	592.5387
☑	147	594.5300	593.5227
☑	148	594.8130	593.8057
☑	149	596.4830	595.4757
☑	150	598.3260	597.3187
☑	151	599.4950	598.4877
☑	152	601.4650	600.4577
☑	153	601.7220	600.7147
☑	154	601.8520	600.8447
☑	156	604.5930	603.5857
☑	159	606.5590	605.5517
☑	160	606.6480	605.6407
☑	161	615.4920	614.4847
☑	162	617.4760	616.4687
☑	163	619.5030	618.4957
☑	165	623.9140	622.9067
☑	166	624.4830	623.4757
☑	168	626.4110	625.4037
☑	170	628.3850	627.3777
☑	173	636.2410	635.2337
☑	175	639.7440	638.7367
☑	179	644.4390	643.4317
☑	180	645.3380	644.3307
☑	188	664.5190	663.5117
☑	201	696.0930	695.0857
☑	209	790.2130	789.2057

Search Parameters

```
Type of search         : MS/MS Ion Search
Enzyme                 : Trypsin
Fixed modifications    : Carbamidomethyl (C)
Variable modifications : Oxidation (HW),Oxidation (M)
Mass values            : Monoisotopic
```

```
Protein Mass           : Unrestricted
Peptide Mass Tolerance : ± 0.5 Da
Fragment Mass Tolerance: ± 0.5 Da
Max Missed Cleavages   : 1
Instrument type        : ESI-TRAP
Number of queries      : 882
```

**Mascot:** http://www.matrixscience.com/
