# Supplementary material for: Identification and characterization of a target antigen recognized by the monoclonal antibody against Opisthorchis viverrini
Source: PLoS One. 2025 May 29;20(5):e0324137. doi: 10.1371/journal.pone.0324137 (PMC12121735; doi:10.1371/journal.pone.0324137)
Supplement: S4 File — (PDF) [file pone.0324137.s006.pdf]

B-cell epitope prediction of myosin between amino acid residues 461-628 utilizing the spatial clustering characteristics of epitopes via a graph attention network (EpiGraph). B cell epitope prediction by capturing spatial clustering property of the epitopes using graph attention network [1].

Residue 461-628

FDENGFEQICINYTNERLQQFFNHHMFVLEQEEYKREKIQWTFIDFGMDLQACIDLIE
KPMGILSILEEECIVPKATDQTFLSKLYDNHLGKSPNFTKPKPPKAGQKEAHFELHHY
AGSVPYTITGWLEKNKDPLNDTVVNLLGGSKDALVSQLFVPVVAESGKKTKG

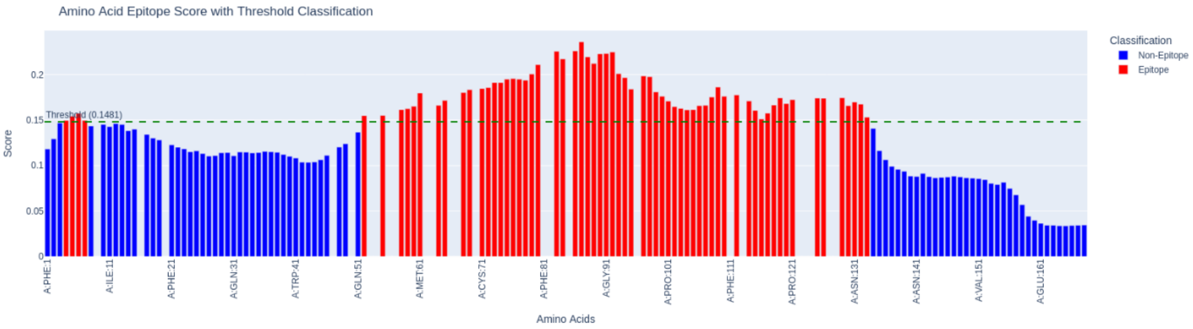

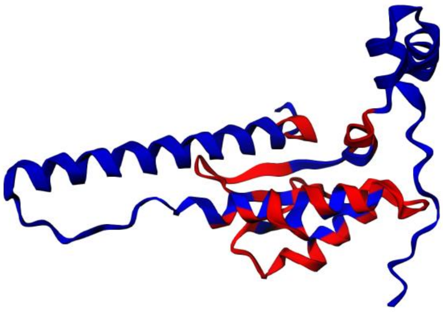

Additionally, the prediction was also conducted on each 200-residue fragment of the *Opisthorchis viverrini* myosin heavy chain, comprising a total of 1932 amino acids. For each fragment, the single-letter polypeptide sequence, amino acid epitope scores with threshold classification, and 3D structure are presented. The B cell recognition patterns were all located on myosin head domain (401-800 amino acid residues).

Residue 1-200

MEPNDPDFKYLGVDRKALLKELANFDSKNVIWVEDEKEGYILADIVETSGDTITVKL
KDGVEKKVKKDDAQQVNPPKFFLIEDMANLTFLNDASVLENLRARYYRQLIYTYSG
LFCVAVNPYKRFPIYTAQVALKYKGKRRSEMPPHIFSISDNAYHNMLQDRENQSILITG
ESGAGKTENTKKVISYFAIVAAAPKKDD

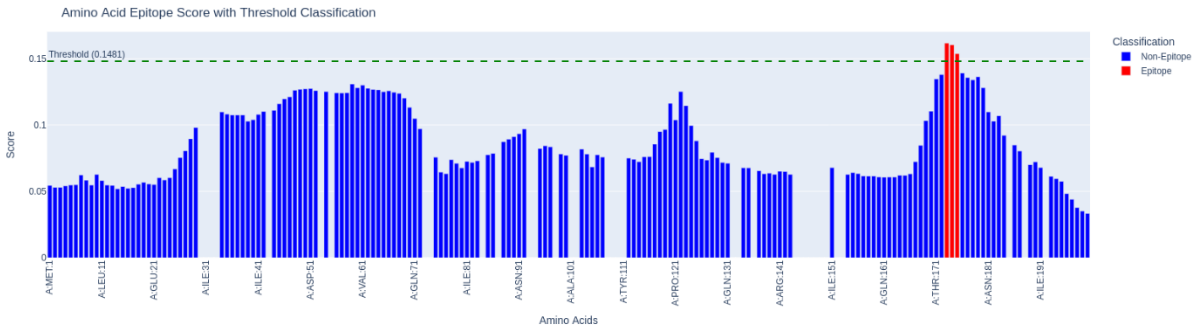

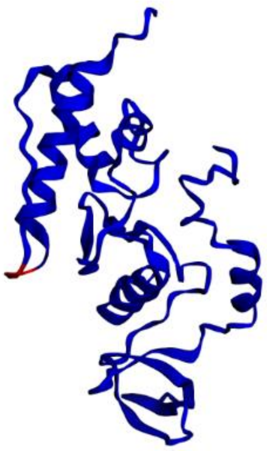

Residue 201-400

DESSKKGSLEDQIVQANPVLEAYGNAKTTRNNNSSRFGKFIRIHFGTTGKIAGADIEH
YLLEKSRVVSQMKGERNYHIFYQLLSDYGKKYHDKLLVAADPGLYSFINQGELTIDG
VDDSEEMRLCDEAFEVLGFSEEEKMSLYKCTTSICNLGEMKFKQRPREEQAEADGTA
ECEKVAFLLGVNAKDLMTSFLKPKVKVG

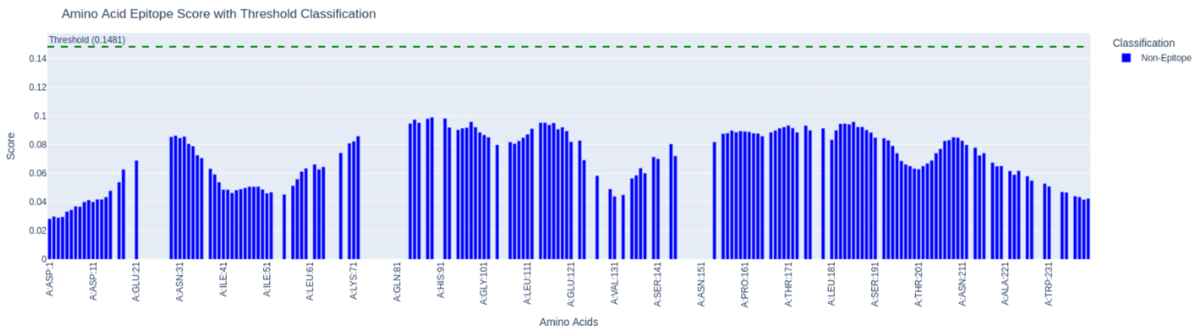

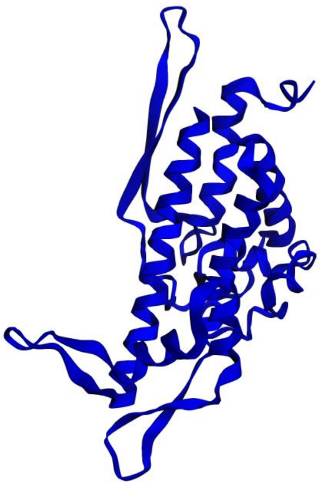

Residue 401-600

TEFVTKGQNLNQVTYAVSALAKSLYNRMFGWLVARVNKTLDTKVKRQFFIGVLDIA
GFEIFDENGFEQICINYTNERLQQFFNHHMFVLEQEEYKREKIQWTFIDFGMDLQACI
DLIEKPMGILSILEEECIVPKATDQTFLSKLYDNHLGKSPNFTKPKPPKAGQKEAHFEL
HHYAGSVPYTITGWLEKNKDPLNDTVV

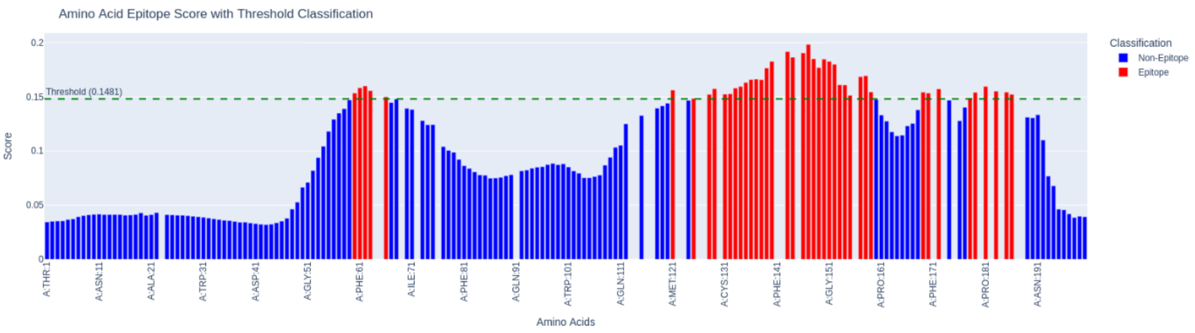

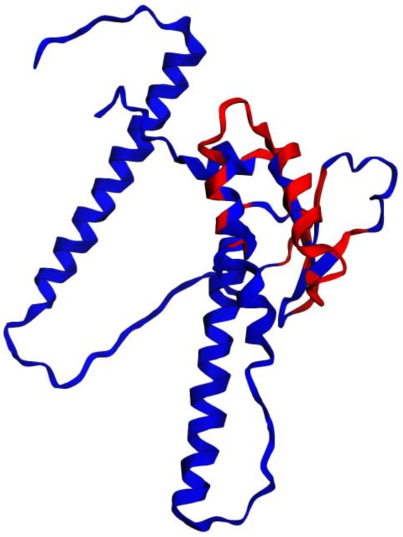

Residue 601-800

NLLGGSKDALVSQLFVPVVAESGKKTKGGSFLTVSFMHRESLNKLMKNLHSTSPHFI
RCIVPNEFKQPGVVDAHLVLHQLHCNGVLEGIRICRKGFPNRMIYSEFKQRYSILAPN
VIPEGFVDGRQVTEKILEATNLDKSLYQCGNTKVFFKAGTLASLEDMRDEKLNVIIAL
FQAEIRGYLMRKQYKKLQDQRVALTLM

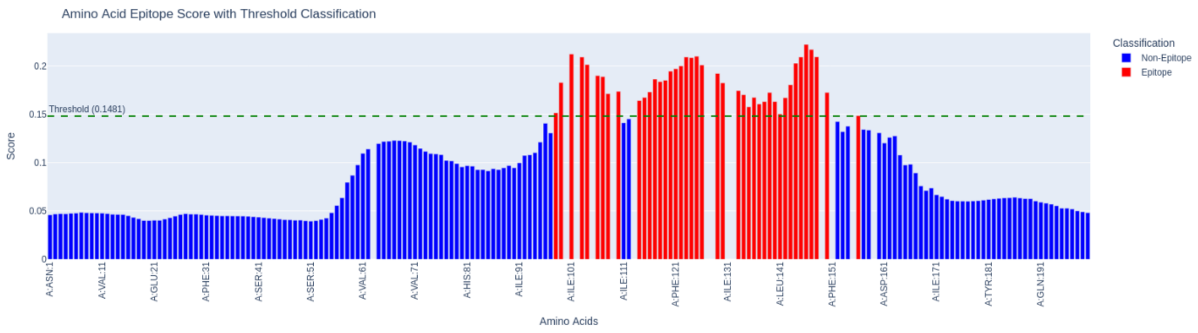

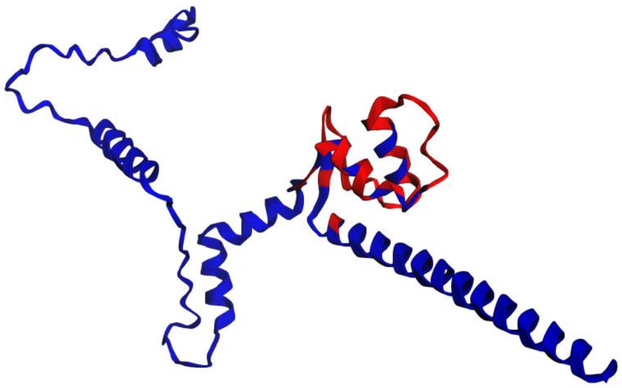

Residue 801-1000

QRNIRKYLMLRNWPWWRLYAKVKPMLNVARQEEEMKKAAEELAKLKEEFEKLEKL
KKELEEQNVTVLQQKNDLFLQLQTEQDSLAEAEEKISNLVLQRGDLETRIKELEERLA
DEEDANSNLNEMKKKLNAEIDELKKDVEDLESSLQKAEQEKQTKDNQIRTLQGEIAQ
QDEQITKLNKDKKALEEQNKRTQEALQAEE

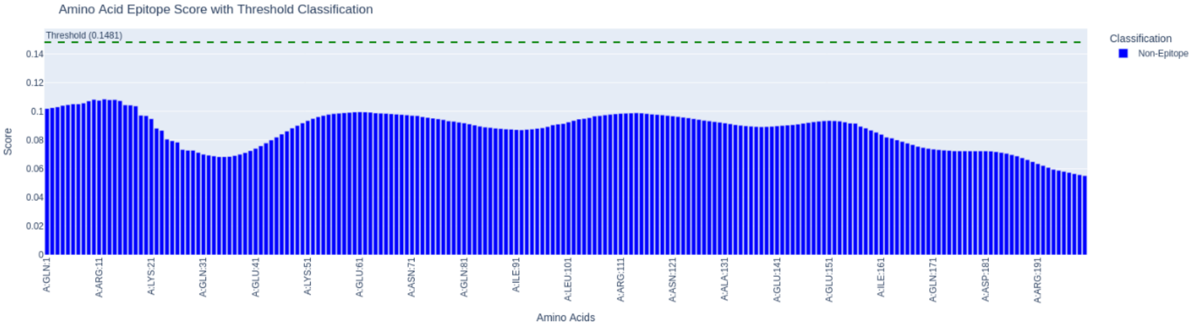

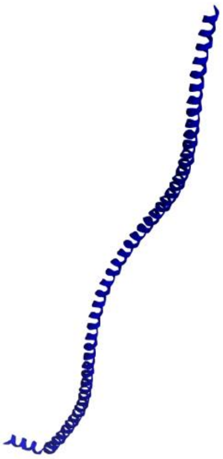

Residue 1001-1200

DKVNHLNKLKAKLESTLDEMEENLAREQKVRADVEKAKRKLEGDLKATQETVDDL
ERVKRDLEEQLRRREVEIGGLNSKFEDEQSIVAQLQRKIKELQTRIQELEEDLEAEQLE
EVVERLEEQDGVNAQQVDLTKKRESELMKLKRDLEDARAQNEQAIAAMRKKQNDS
VNEMADQLDQLNKAKAKAEKERNQFKAEVDD

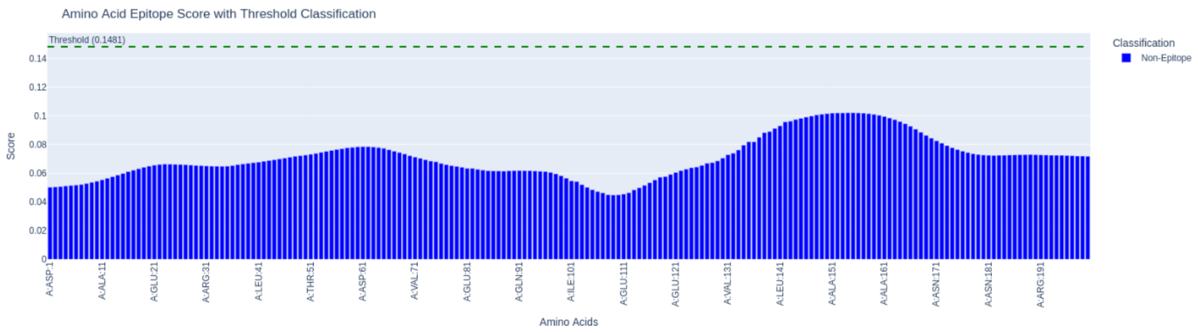

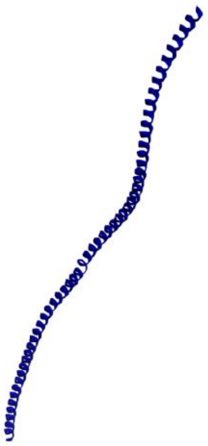

Residue 1201-1400

LHSQLESLSKAKMNAEKNTKGLETQIQELHAKLDETTNQLSEQASGKARNSQENAE
LQRQLEEAESQLSQLSKVKQQLSAQLEEARHSLEEESRMKAKLNSEVRNLTSDLDSL
RESLEEEQSGKADLQRQLQKMQGELQQLRSRGGGGDVRSEEMEELKRRLNGKIQEL
EAECESAKSKCGQLEKTKARLQGELEDLMVD

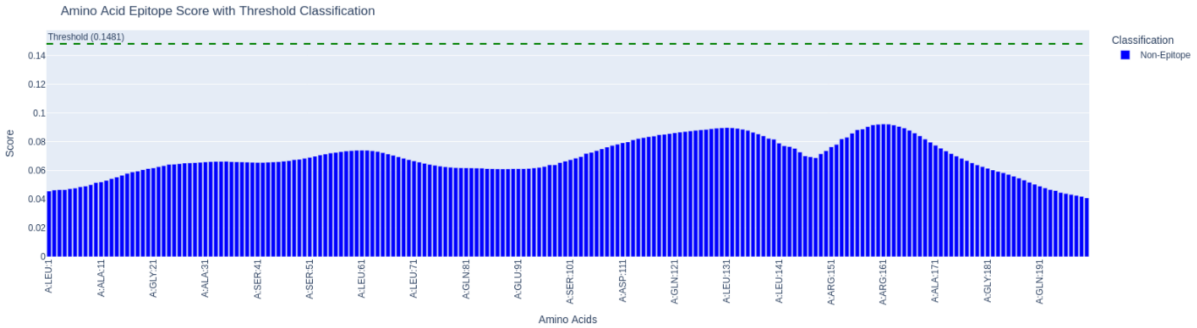

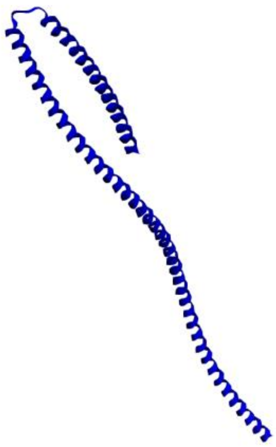

Residue 1401-1600

VERANGMASQLERKQANFNRTLAEWQKKFADSQAELENSQRDARNQSTEMFRLKA
QLEEAHEQIEAVRRENKNLSDEIHDLTEQLGEGGRSVHEIDKARRRLEMEKEELQAA
LEEAEGALEQEEAKLQRAQLEMSQVRQEIDRRLAEKEEEFEATRKNHQRAMESQQA
SLEAEAKGKAEALRIKKKLEQDINELEVSLDA

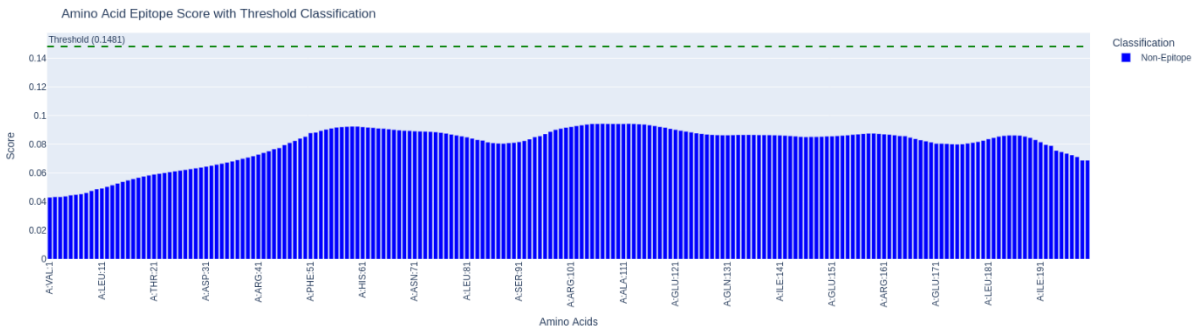

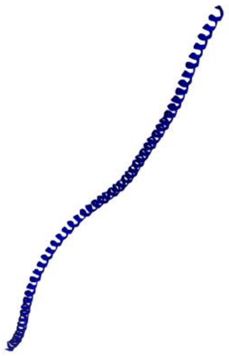

Residue 1601-1800

ANRNRAEQEKNVKKLQQQVREVQGQLEDEQRQRDDIREQFQAAERRANMMSGEIE
ELRTALEQAERGRKMAEAERAEAADRATELSTQAASLAAQKRKLEADLAAMQADL
EEAANEAKQADERAKKAMADSSRVFEEIRQEQEHTQHVEKARKQLELQVKEMQAR
LEDTESGAAKNGRKAVGKLEQRVRELETELEAEQR

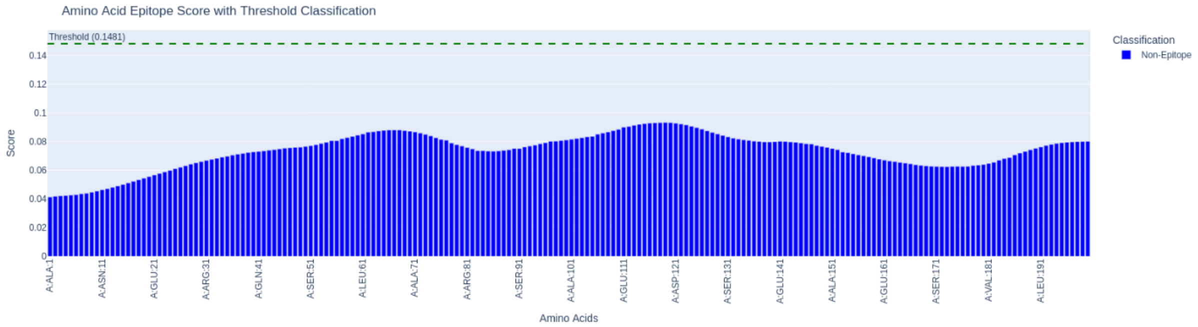

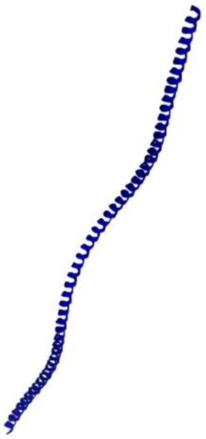

Residue 1801-1932

RHGETLKNLRKVDRRMKEISMQSEEDKKNHERMQELVEKLQGKIKTYKRQVEEAEE
IAAINLAKYRKIQHEIEDAEERADQAEQALQKLRAKNRSSVSAARGVSAAPAGAGPA
NRARKPAASHQRELNFQPK

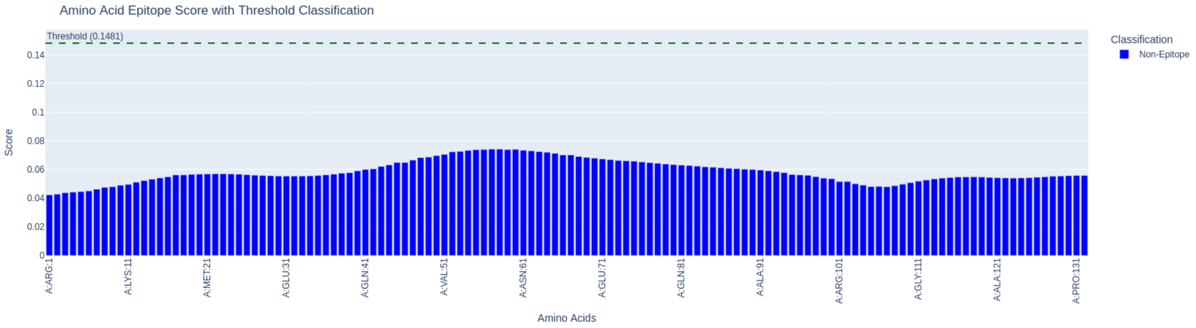

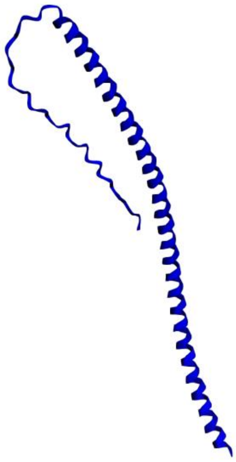

Reference

1. Choi S, Kim D. B cell epitope prediction by capturing spatial clustering property of the epitopes using graph attention network. Sci Rep. 2024;14(1):27496. doi: 10.1038/s41598-024-78506-z
