# Supplementary material for: Identification and characterization of a target antigen recognized by the monoclonal antibody against Opisthorchis viverrini
Source: PLoS One. 2025 May 29;20(5):e0324137. doi: 10.1371/journal.pone.0324137 (PMC12121735; doi:10.1371/journal.pone.0324137)
Supplement: Attachment — Submitted filename: Final_Techasen et al_241113_ps PC.docx [file pone.0324137.s007.docx]

**Identification and characterization of a target antigen recognized by the monoclonal antibody against *Opisthorchis viverrini***

Anchalee Techasen^1,2^, Chanika Worasith^1,3^, Duangkamon Muengsaen^1^, Jiraprapa Ponglong^1^, Panupong Mahalapbutr^4^, Napat Kongtaworn^5^, Thanyada Rungrotmonkol^5,6^, Kanoknan Khongsukwiwat^1,7^, Phattharaphon Wongphutorn^1,7^, Chompunoot Wangboon^8^, Chutima Homwonk^1,7^, Sujittra Chaiyadet^9^, Thewarach Laha^7^,  [Sutas Suttiprapa](https://pubmed.ncbi.nlm.nih.gov/?term=Suttiprapa+S&cauthor_id=32659283)^9^, Chadamas Sakonsinsiri^1,4^, Paiboon Sithithaworn^1,7* ¶^ and Raynoo Thanan^1,4* ¶^

^1^Cholangiocarcinoma Research Institute, Khon Kaen University, Khon Kaen, Thailand

^2^Faculty of Associated Medical Sciences, Khon Kaen University, Khon Kaen, Thailand

^3^Department of Adult Nursing, Faculty of Nursing, Khon Kaen University, Khon Kaen, Thailand

^4^Department of Biochemistry, Faculty of Medicine, Khon Kaen University, Khon Kaen, Thailand

^5^Program in Bioinformatics and Computational Biology, Graduate School, Chulalongkorn University, Bangkok, Thailand

^6^Center of Excellence in Structural and Computational Biology, Department of Biochemistry, Faculty of Science, Chulalongkorn University, Bangkok, Thailand

^7^Department of Parasitology, Faculty of Medicine, Khon Kaen University, Khon Kaen, Thailand

^8^School of Preclinic, Institute of Science, Suranaree University of Technology, Nakhon Ratchasima, Thailand

^9^Department of Tropical Medicine, Faculty of Medicine, Khon Kaen University, Khon Kaen, Thailand

*Corresponding authors:

E-mail: [raynoo@kku.ac.th](mailto:raynoo@kku.ac.th) (RT) and [paib_sit@kku.ac.th](mailto:paib_sit@kku.ac.th) (PS)

**Abstract**

*Opisthorchis viverrini* (Ov) infection caused opisthorchiasis, which posed an important risk for the development of cholangiocarcinoma (CCA). Therefore, it is crucial to focus on the primary prevention and control of opisthorchiasis in order to control CCA effectively in Thailand and other endemic regions. A recent diagnostic method of antigen detection using monoclonal antibody-based enzyme-linked immunosorbent assay (mAb-ELISA) has the potential for rapid mass screening of opisthorchiasis. Nevertheless, the specific antigen(s) in Ov adult worm recognized by mAb has not been determined. In this study, we aimed to identify and characterize the target molecule(s) of our in-house Ov-specific monoclonal antibody. The specific antigenic bands formed by the reaction of Ov adult worm extract and mAb were detected using western blot analysis. The protein band was identified as the myosin head (MH) of Ov using LC-MS/MS analysis. The reactivity of the recombinant myosin head (rMH) was comparable to that of the crude Ov antigen when evaluated using mAb-ELISA at similar protein concentrations. Moreover, the binding ability between MH and mAb was confirmed using *in silico* analysis. Whether the target antigen rMH is detectable in urine and/or fecal samples of individuals with opisthorchiasis should be determined in the future. The results reported here indicate that rMH could potentially substitute for Ov crude antigen, thereby avoiding the use of laboratory animals to produce Ov adult worms.

**Introduction**

*Opisthorchis viverrini* (Ov) infection is a major factor contributing to the development of cholangiocarcinoma (CCA) in the Greater Mekong sub-region, especially northeastern Thailand [1]. Raw cyprinoid fish consummation is a high risk for Ov infection. Up to date, many studies revealed that Ov infection induces bile ducts and liver injury leading to periductal fibrosis (PDF), which consequently develops CCA in animal models [2, 3]. Similarly, Ov infection and PDF are recognized as the CCA risk factors in human subjects [4]. CCA is the leading cause of mortality in Thailand, Laos, Vietnam, and Cambodia. Therefore, it is crucial to promptly detect cases of Ov infection and PDF individuals in order to promote CCA control measures. Currently, PDF and CCA are screened using abdominal ultrasonography by the radiologists. Comprehensive analyses of tumor markers in the serum and urine of PDF subjects were conducted using mass spectrometry-based techniques [5]. However, simple biochemical techniques remain required to identify and validate these biomarkers for the purpose of screening individuals who are at high risk of developing CCA.

Parasitological and molecular techniques have been developed to detect parasite eggs and DNA in fecal samples. One of the most reliable methods for diagnosing Ov infection is the egg detection by formalin ethyl-acetate concentration technique (FECT). This procedure accurately quantifies Ov eggs in feces and is considered the "gold standard" for diagnosis of Ov infection. The FECT approach revealed limited analytical sensitivity. Quantification of Ov eggs carries a technical error risk due to variations among microscopists. Ov eggs are similar in size to those of minute intestinal flukes (MIFs) [6], requiring fresh feces samples for accurate results. As an alternative, PCR-based approaches were developed to detect parasite DNA in fecal samples using molecular techniques [7, 8]. These methods exhibit high sensitivity and accuracy when used to fecal samples from an Ov-infected animal model. However, due to the presence of numerous components in human feces that can disrupt DNA extraction and PCR conditions, they may not be appropriate for all patients [9, 10].

We have previously developed immunological methods using ELISA-based techniques to detect Ov-antigens. The anti-Ov monoclonal antibody-producing hybridoma cell lines were induced and developed in-house by Sithithaworn *et al.* [11]. The monoclonal antibody-based detection and quantitation of Ov antigens in urine samples showed much greater diagnostic sensitivity and specificity than the current gold standard method. The monoclonal antibody-based ELISA assay offers the potential to revolutionize the diagnosis of liver fluke infection and provide an effective tool for control and elimination of these tumorigenic parasites.

The in-house monoclonal antibody (Thai patent No. 2872) has been used for detection and quantification of opisthorchiasis in animal and human studies [11, 12]. Due to its user-friendly nature and the noninvasive collection of urine samples, this approach was subsequently enhanced to serve as the diagnostic test kit for opisthorchiasis in Thailand and the neighboring countries [13].

The Ov crude antigens were applied as the positive control for the purpose of standardization of the detection method. In order to produce Ov crude antigens, infective metacercariae were extracted from cyprinoid fish and introduced into hamsters via oral injection. Hamsters infected with Ov were sacrificed three to four months after infection, and the adult worms were collected from the livers. In this study, to save time and animals required to produce Ov crude antigens, the monoclonal antibody-targeted protein was identified, and the recombinant protein was produced and characterized.

**Materials and methods**

**Animal ethics**

The protocols involving laboratory animals for production of the adult *O.viverrini* to prepare crude antigen was approved by the Animals Ethic Committee, Faculty of Medicine, Khon Kaen University (approval number AEKKU 98/2562). The protocols for monoclonal and polyclonal antibodies productions were also approved by the Animals Ethic Committee, Faculty of Medicine, Khon Kaen University (approval number AEKKU 43/25620). Humane endpoints were implemented with daily health assessments of the animals to ensure their well-being. The procedure adhered strictly to the guidelines set forth by the National Research Council of Thailand for the Care and Use of Laboratory Animals.

**The crude OV antigens preparation**

Metacercariae of Ov was extracted from the naturally infected cyprinoid fish, which were obtained from an endemic area in northeastern Thailand. Fish were chopped into small pieces by an electrical blender, and, the minced fish were enzymatically digested in a freshly prepared solution of 0.25% pepsin and incubated at 37°C for 1 h in a shaking water bath. After digestion, the digested material was filtered and washed several times. The Ov metacercaria were sorted from the final sediment and identified under a dissecting microscope. The metacercariae were pooled and administered orally via gastric intubation to five inbred male Syrian golden hamsters (*Mesocricetus auratus*) (50 cysts per animal) which yielded about 50% worm recovery according to the previous study [11]. The infected hamsters were housed and maintained at the animal center, Faculty of Medicine, Khon Kaen University. While the metacercariae dose was known to cause no effect on the well-being of the hamsters, the animals were monitored daily for any abnormal clinical, physiological, or behavioral signs, with criteria for humane endpoint intervention in place. No significant morbidity was observed over the 12-week infection period. The animals were then euthanized with isoflurane prior to removal of the liver to recover Ov adult worms. The intact worms cleaned and homogenized on ice and centrifuged at 14,000 rpm at 4°C for 30 min, and the supernatant obtained was stored at -20 ℃ until required. The protein concentration of the crude somatic Ov antigen was determined using the BCA protein assay kit (Thermo Fisher Scientific, MA, USA).

**Monoclonal and polyclonal antibody production**

The monoclonal antibody (mAb) clone KKU 505 produced previously was used in this study [11]. The mAb was specific to *O. viverrini* had no cross reactivity with *Fasciola* sp., *Taenia* sp., echinostomes, Spargarnum, *Dipylidium caninum*, *Angiostrongylus cantonensis*, hookworm, and *Strongyloides stercoralis* was produced in BALB/c mice. Following acclimatization of the animals for 7 days, the hybridoma cells (1-3×10^6^ cells/ml PBS/animal) were intraperitoneally injected into two pristane-primed inbred male BALB/c mice. The ascitic fluid containing mAb was collected at 2-4 weeks after injection by the experienced animal technicians at the animal canter. The humane endpoint was applied by daily inspection of the animals for evidence of abnormal clinical, physiological, and behavioral signs, once the animal reached the endpoint criteria euthanasia can be done within 3 hours. No animal died before meeting the criteria for euthanasia. The ascetic fluid was centrifuged at 2,000 rpm for 10 min. and the supernatant was aliquoted and kept at -80˚C until use.

The rabbit IgG polyclonal antibody against Ov was generated by immunizing two New Zealand white rabbits (*Oryctolagus Cuniculus*) with crude Ov antigen. After a week of acclimatization, the animals were first immunized with a subcutaneous injection of 100 µg of Ov antigen suspended in complete Freund's adjuvant (CFA, SIGMA® (St. Louis, USA). Four weeks later, the second immunization was performed using 100 µg of Ov antigen mixed with incomplete Freund's adjuvant (IFA, SIGMA® (St. Louis, USA)), followed by a booster injection of the same antigen formulation two weeks after that. At week 10 after the initial immunization, the rabbits were euthanized using isoflurane, and blood was collected via cardiac puncture by the experienced animal technician. No animals had abnormal physiological signs before the schedule for euthanasia. The serum was separated and stored at -20°C for future use.

**Sodium dodecyl sulfate polyacrylamide gel electrophoresis (SDS-PAGE)**

The protein sample was mixed with SDS loading sample buffer, which had a final concentration of 62.5 mM Tris-HCl pH 6.8, 20% glycerol, 2% SDS, and 5% β-mercaptoethanol. The mixture was then boiled to 100 ˚C for 10 min and subjected to electrophoresis on either an 8% or 6% SDS-PAGE gel. For the cut-off target size of protein bands, electrophoresis was performed at 40 mA (per gel) for 40 min, or until the bromophenol blue dye reached the bottom of the gel, or by following a pre-stained protein ladder. SDS-PAGE gel was stained with Coomassie Brilliant Blue (CBB) R-250 solution containing 10% acetic acid with 25% methanol for 1 h. Gels were de-stained in 8% acetic acid for 1 h or until cleared the background.

**Western blot analysis**

After electrophoresis, proteins were transferred from SDS-PAGE gel to PVDF membrane (Immobilon®, Millipore, MA, USA). The electroblotting was carried out at 30 V, 0.1 Amp, overnight at room temperature with a Bio-Rad Transblot apparatus and transfer buffer (25 mM Tris, 192 mM Glycine, 20% v/v methanol, pH 8.3). After transfer, the PVDF membrane was incubated in 5% skim milk with TBS buffer for 1 h to block non-specific protein binding. Following a blocking step, the membrane was probed with a mAb that was obtained from ascitic fluid (1:1,000 in 1% skim milk) or culture media (1:100 in 1% skim milk) for 1 h on the shaker. Then, the membrane was washed twice with 0.05% Tween-20 in TBS buffer (TTBS) and washed with TBS buffer for 5 min on the shaker. After that, a secondary antibody, anti-mouse IgM conjugated-HRP (1:1,000 in 1% skim milk), was added and incubated for 1 h on the shaker. Following a further washing process, a chemiluminescence substrate specific to the HRP enzyme was applied onto the membrane. The HRP enzyme then modifies the substrate, resulting in the emission of light as detected by the Chemiluminescence Imaging Systems (Amersham™ Imager 600).

**Digestions of O-glycans and N-glycans**

Digestions of O-glycans and N-glycans were performed using O-glycosidase and PNGase F protocols from the New England BioLabs^®^ Inc. To denature glycoproteins, the reaction mixture containing 10 µg of glycoprotein sample, 1 µl of glycoprotein denaturing buffer (10×) and deionized water were mixed in a total reaction volume of 10 µl in a microtube. The reaction mixture was heated at 100 °C for 10 min. After that, 2 µl of GlycoBuffer-2 (10×), 2 µl of NP-40, and glycosidase enzyme (1 µl of O-glycosidase and 2 µl of neuraminidase or 1 µl of PNGase F) were added, and deionized water was added for up to 20 µl of total volume. The reaction mixture was incubated at 37 °C for 1 h. The results of digestion were checked using SDS-PAGE and western blot analysis.

**Liquid chromatography coupled with tandem mass spectrometry (LC-MS/MS)**

Expected protein targets of the mAb were identified using liquid chromatography tandem mass spectrometry (LC-MS/MS). Protein bands of interest were cut from SDS-PAGE gel (CBB stained gel) and placed into a 1.5 ml microcentrifuge tube. The sample in gel was digested with trypsin. After digestion, the samples were cleaned up using C18 Zip Tips^TM^ (Millipore) and analyzed with a nano-liquid chromatography system (EASY-nLC II, Bruker, MA, USA) coupled to an ion trap mass spectrometer (Amazon Speed ETD, Bruker) combined with an ESI nanosprayer. The ESI-TRAP instrument was calibrated at the m/z range 50−3000 using an internal calibration standard. The autosampler loaded a 3 µL sample volume at a flow rate of 500 nL/min onto an EASY-Column, 10 cm, ID 75 µm, 3 µm, C18-A2 (Thermo Scientific, MA, USA). DI water was present in mobile phase A, while acetonitrile and 0.1% formic acid were present in mobile phase B. The gradient was 5–35% B for 50 min, and then 80% B for 10 min. The experiment was conducted using tryptic digest (50 fmol) of bovine serum albumin (BSA) as the standard control. The ion trap device was operated by the HyStar v.3.2 software package from Bruker Daltonics. Compass Data Analysis v.4.0 was used to examine LC-MS/MS spectra. For additional searching in the MASCOT application, compound lists were exported as Mascot generic files (mgf).

**Bioinformatics and sequence analysis**

Amino acid sequences from LC-MS/MS analysis were searched for homology versus gene and protein in public databases using Blast search at <https://blast.ncbi.nlm.nih.gov/Blast.cgi> [14]. Sequences were edited and analyzed for coding regions and restriction maps using BioEdit.

**Recombinant protein production**

The targeted gene of myosin head was synthesized and cloned into the pET-15b vector using the Nde I site (CATATG) (GeneScript Centennial Ave. Piscataway, NJ, USA). The *Escherichia coli* stain TOP10 was used to replicate the recombinant gene. The target clones, full Ov-myosin head, was annotated from the genome sequence of Ov (GenBank acc. XM_009176183) (full Ov-myosin head, spanning nt. 120-5,918 encoding 1,932 aa and partial Ov-myosin head, spanning nt. 120-2,256 encoding 712 aa) were verified by Nde I restriction digestion or PCR with T7 promotor primers (data not shown). The recombinant plasmids were transformed into *E. coli* stain BL21 (DE3) (Novagen Madison, WI, USA) for target protein expression. The foreign plasmids were inserted through the cell wall by the heat shock method at 42 °C and the transformed cells were spread on LB agar supplemented with ampicillin (1 µg/ml) and incubated at 37 °C with horizontal shaking 200 rpm overnight. Single colonies were picked and grew in 200 ml LB broth with ampicillin (1 µg/ml) at 37 °C until the OD600 approximate to 0.6. After that, the recombinant protein expression was induced by adding 1 mM of isopropyl β-D-1-thiogalactopyranoside (IPTG) and incubated at 37 °C overnight with shaking. After cell growth, the cells were aliquoted and collected by centrifugation at 5,000 *g* for 15 min at 4 °C. The supernatant was removed, and cell pellets were kept in -20 °C until used.

**Isolation of the recombinant protein by serial extraction**

The bacteria cell pellets (induced myosin expression) were added by 2 mL of the extraction buffer 1 (50 mM NaH_4_PO_4_.2H_2_O, 75 mM NaCl, pH 8.2) and sonicated for 5 min on ice. Then, the tubes were centrifuged, and supernatant was removed (supernatant 1; S1). The pellets were further washed again with 2 mL of extraction buffer 1, sonicated, centrifuged, and the supernatant was removed. After washing, the pellets were re-suspended in the extraction buffer 2 (50 mM NaH_4_PO_4_.2H_2_O, 150 mM NaCl, pH 8.2) and sonicated for 5 min on ice. Then, the tubes were centrifuged, and supernatant was removed (supernatant 2; S2). After that, the pellets were re-suspended in the extraction buffer 3 (20 mM NaH_4_PO_4_.2H_2_O, 500 mM NaCl, 2M thiourea, 7M urea, pH 8.0) and mixed by a vertex mixer. The mixtures were labeled as supernatant 3 (S3). Then, to salt out proteins, S3 was mixed with 100% ammonium sulfate, to make up a final concentration of 30% ammonium sulfate. The mixtures were centrifuged at 10,000 rpm at 4 °C for 10 min. The supernatant was removed, and the pellets were washed 3 times with acetone. Subsequently, the pellets were re-dissolved at 4 °C in PBS for overnight, and the mixtures were labeled as supernatant 4 (S4). SDS-PAGE was then applied to separate supernatant 1-4, and S4 was used for the capture ELISA analysis. The leftover samples were kept at -20 °C.

**Capture enzyme linked immunosorbent assay (Capture-ELISA)**

The protocol for capture ELISA was similar to those described previously using the same mAb clone (KKU505) and rabbit anti-Ov IgG [11, 12, 15]. Polystyrene microtiter plates (NUNC, Roskilde, Denmark) were sensitized overnight at 4 °C with 5 µg/ml of mAb of a clone KKU505, which is specific for Ov antigen. Plates were washed three times with 0.05% Tween 20 (NSST), and uncoated sites were blocked with 5% skimmed milk in carbonate buffer, pH 9.6. After incubation for 1 h at 37 °C, spiked serially diluted myosin in PBS was added, and the plates were incubated at 37 °C for 2 h. After five washes with NSST, 10 µg/ml of IgG rabbit anti-crude Ov antigen was added to each plate, and then incubated at 37 °C for 1 h. Following the addition of biotin goat anti-rabbit IgG (dilution 1:4,000) (Invitrogen, CA, USA), the plates were incubated at 37 °C for 1 h. After washing three times, streptavidin-horseradish peroxidase conjugate (dilution 1:5,000) (GE Healthcare, Buckinghamshire, UK) was added, and the plates were incubated at 37 °C for 1 h. The plates were washed, and the substrate solution (*o*-phenylenediamine hydrochloride) (Sigma, St. Louis, MO, USA) was added and incubated for 20 min in the dark at room temperature. The enzyme reaction was stopped with 2 M sulfuric acid, and the optical densities (OD) were read spectrophotometrically at 492 nm with an ELISA reader (Tecan Sunrise Absorbance Reader, Austria).

**Structural modeling and molecular docking**

The amino acid sequences of mAb KKU505 and the Ov myosin head were obtained from Siripanthong *et al.* [16]. The 3D structures of both proteins were predicted using ChimeraX AlphaFold [17]. The best model of each protein was selected for molecular docking using the ZDOCK webserver (https://zdock.umassmed.edu/) [18]. The docked structure with the highest docking score was selected for further study. Note that the tail of the myosin head (residues 771-800) was removed from the docked complex to reduce computational time. The protonation state of the protein-protein complex was predicted at pH 7.0 using the PROPKA web server [19]. The ff14SB force field parameter [20] was applied for protein-protein complex. The Na^+^ ions were added to neutralize the system. Then, the complex was solvated using the TIP3P water model in an octahedral box [21]. After that, the system was minimized using the steepest descent and the conjugated gradient methods.

**Molecular dynamics (MD) simulation**

MD simulation of the complex was performed using the AMBER22 software package [22]. Firstly, the system was heated up from 100 to 310 K for 100 ps with a canonical ensemble (*NVT*) with the time step of 2 fs. Secondly, the water molecules and the complex were equilibrated for 100 and 500 ps, respectively. Finally, the system was simulated for 100 ns under the isothermal-isobaric ensemble (*NPT*) at 1 atm and 310 K with a time step of 2 fs. The pressure and the temperature of the system were maintained using the Berendsen barostat [23] (pressure-relaxation time = 1 ps) and the Langevin thermostat [24] (collision frequency = 2 ps^-1^), respectively. The SHAKE method [25] was applied to constrain all bonds involving hydrogens. The particle-mesh Ewald method [26] was used to manage the long-range electrostatic interactions. After completing the MD simulation, the MD trajectories were gathered for analysis of the root-mean-square displacement (RMSD) using the CPPTRAJ module in the AMBER22 program [22]. The intermolecular interactions between KKU505 Fab and myosin head were evaluated using the PDBsum web server.

**Results**

**Detection and identification of the targeted proteins recognized by monoclonal antibody**

Using SDS-PAGE, the crude Ov antigens of adult worms were separated. The CBB-stained 6% gels and the western blot results were shown in Figs 1A-B. The primary antibody applied in these assays was a mAb derived from KKU505. As illustrated in Figs 1A-B, the candidate protein band was detected at approximately over 180 KD on 6% gel SDS-PAGE. The gel stained with CBB for the Ov crude antigens (Fig 1A) revealed two protein bands that were clearly stained. The arrow indicated the selected protein band, which was analyzed further using LC-MS/MS. Ov-crude antigens treated with N-glycosidase, O-glycosidase, and N-&O-glycosidases did not affect the immunoreactivity patterns (Fig 1C), indicating that the targeted epitopes are in the peptide sequences but not in glycan parts.

**Fig 1.** **Separation of Ov crude antigens using SDS-PAGE and western blot analysis.** (A) CBB staining of 15 μg of Ov crude antigens separated using 6% acrylamide gels of SDS-PAGE. (B) Western blot analysis of 1 μg of Ov crude antigens of using mAb KKU 505 clone as primary antibody. Arrows indicate the selected protein for LC-MS/MS analysis. (C) Western blot analysis of Ov crude antigens after treatments with glycosidase enzymes. MW = standard protein molecular weights, KDa = kilodalton and Crude-Ov = Ov crude extract

The results of the LC-MS/MS analysis for the selected protein band are presented in S1 File. The protein was identified as the myosin head of Ov with protein sequence coverage of 21% of the OON15278 sequence. The myosin head contains a monoisotopic mass (Mr) of 222.414 KD. Thus, the myosin head of the parasite was selected for further characterization.

**Recombinant Ov myosin head protein production and characterization**

The full mRNA sequence of Ov myosin head was obtained from the shotgun genome sequence of the Ov genome project submitted to GenBank (GenBank acc. XM_009176183) [27]. The Ov myosin head expression (full Ov-myosin head, spanning nt. 120-5,918 encoded 1,932 aa and partial Ov-myosin head, spanning nt. 120-2,256 encoded 712 aa) in pET15b plasmid vectors were constructed by GeneScript Centennial Ave. Piscataway, NJ 08854, USA using pET-15b vector. The target clones were verified by Nde I restriction digestion or PCR with T7 promotor primers (data not shown). Around 75 KD band of the partial Ov-myosin head and ~200 KD protein band of the full Ov-myosin head were detected in *E. coli* BL21 after induction of the genes in pET-15b vector expression condition as shown in Fig 2A (the recombinant proteins were indicated by arrows). The LC-MS/MS analysis results of the ~75 KD and ~200 KD protein bands (Fig 2A) were confirmed to be the myosin head of Ov as shown in the S2 File. In addition, as shown in Fig 2B, the recombinant protein of the full Ov-myosin head reacted with the monoclonal antibody as determined by capture ELISA, whereas the partial Ov-myosin head did not react with it. These findings show that the monoclonal antibody has no reactivity towards the recombinant partial Ov-myosin head protein or the bacterial proteins, although it has a reactivity with the recombinant full Ov-myosin head protein. Since the crude protein isolated from *E. coli* BL21 transfected with the full Ov-myosin head exhibited a lower intensity (OD492) in the capture ELISA in comparison to the crude Ov antigens, full Ov-myosin head protein purification is necessary to increase the immunoreactivity.

**Fig 2. Recombinant Ov-myosin head production in *E. coli* BL21.** (A) CBB staining 8% gel of protein mixtures extracted from *E. coli* BL21 (1), *E. coli* BL21 with partial Ov-myosin head transfection (2), and *E. coli* BL21 with full Ov-myosin head transfection (3). Black arrow indicates the partial Ov-myosin head protein and blown arrow indicates the full Ov-myosin head protein (B) mAb-ELISA of proteins crude extract from *O.viverrini* (Crude-Ov), *E. coli* BL21 with partial Ov-myosin head expression (Crude-Ec partial rMH), and *E. coli* BL21 with full Ov-myosin head expression (Crude-Ec full rMH).

**Isolation, purification,** **and immunoreactivity of recombinant full length-Ov myosin head protein**

The recombinant full Ov-myosin head protein was purified by the serial extraction as described in the Materials and Methods section. The single band of recombinant full Ov-myosin head protein was obtained after the final step of purification (Fig 3A). Immunoreactivity of rMH was compared to that of crude Ov antigens using the capture mAb-ELISA (Fig 3B). The immunoreactivity of rMH was substantially higher than that of crude Ov antigens at the concentration range from 0.006 to 12.5 μg/mL. However, at the protein concentrations exceeding 25 μg/mL, the ELISA intensities of rMH were comparable to those of crude Ov antigens (Fig 3B).

**Fig 3. Isolation, purification, and immunoreactivity of recombinant full length-Ov myosin head.** (A) CBB staining 10% gel of proteins (1 µg/well) from supernatant 1 (S1), supernatant 2 (S2), supernatant 3 (S3) and recombinant Ov myosin head (rMH) obtained from the serial extraction of the induced Ov myosin head expression in *E. coli* BL21. (B) mAb-ELISA of Crude-Ov and rMH curves. MW = standard protein molecular weights, KDa = kilodalton, Crude-Ov = Ov crude extract, rMH = recombinant full length Ov myosin head. ^*^*P*<0.05, ^**^*P*<0.01, and ^***^*P*<0.001 compare to crude-Ov.

**Molecular modeling of KKU505 Fab in complex with Ov myosin head**

To elucidate the binding mechanism of KKU505 Fab against Ov myosin head, molecular docking was first conducted using the ZDOCK Server. As shown in Fig 4A, both chains A and B of KKU505 Fab could bind to the myosin head with the ZDOCK score of 942.182. To evaluate further the structural stability of the KKU505Fab/myosin head complex in an aqueous environment, MD simulation was performed. As shown in Fig 4B, the protein-protein complex reached the equilibrium state after 20 ns with the RMSD fluctuation of 3.5 to 5.0 Å, suggesting the high stability of the complex in water solution. The protein-protein interactions between KKU505 Fab and the Ov myosin head were predicted using the PDBsum. The results (Fig 4C) showed that the chain A of KKU505 Fab can form 33 non-bonded contacts, whereas the chain B of KKU505 Fab can form 58 non-bonded contacts with the Ov myosin head. In addition, seven hydrogen bonds were detected between chain B of KKU505 Fab and Ov myosin head: (i) Lys275 and Asn601 (two bonds), (ii) Pro274 and Asn 601, (iii) Tyr316 and Glu621, (iv) Tyr319 and Glu621, (v) Gln270 and Lys398, and (vi) Asn268 and Lys627. Taken together, these data indicate that KKU505 Fab could interact with Ov myosin head with high structural stability.

**Fig 4.** **Molecular modeling of KKU505 Fab in complex with Ov myosin head.** (A) Docked structure of KKU505 Fab in complex with myosin head. (B) Time evolution of RMSD. (C) Protein-protein interaction profile between KKU505 Fab and myosin head.

**Discussion**

Currently, recombinant technology has brought unique impacts on the advancement of human health research. By virtue of this technology, crucial proteins can be produced safely, affordably, and sufficiently. It offered new opportunities for innovations to produce a wide range of protein products. Recombinant technology has been used to identify target proteins of *O. viverrini* (Ov) recognized by antibodies. Using human CCA sera, Senawong and colleagues [26] isolated 22.8 kDa of Ov calcium-binding EF-hand protein (Ov CaBP) from the adult stage Ov cDNA library. Ov CaBP and *Clonorchis sinensis* CaBP, both measuring 22.8 kDa, exhibited 98% amino acid identity and were classified as a novel class of CaBP EF-hand proteins. Additionally, a soluble protein known as recombinant Ov CaBP protein (rOv CaBP) was produced, and an antibody specific to this rOv CaBP protein could detect Ov CaBP in the Ov somatic extracts but not in the Ov ES products [28]. Likewise, from an Ov cDNA library, Suttiprapa and colleagues [27] identified a cDNA of 23.57 kDa thioredoxin peroxidase (Ov TPx) and produced a recombinant protein of it. Using an antibody raised against Ov TPx, they detected that the native Ov TPx protein is expressed in the eggs, metacercariae, and adult various developmental stages of adult worms, as well as excretory-secretory products secreted by adult Ov [29].

In this study, we successfully identified and characterized a novel 222 KD of Ov protein, myosin head (MH), which is a target molecule of our in-house mAb against Ov. The crude Ov antigens were extracted from the adult worms, and the specificity of the protein pattern was assessed using western blot with an in-house mAb derived from KKU055 cell culture media and ascitic fluid. The targeted bands were detected. Since immunoreactivity patterns were unchanged after glycosidases-treatments of Ov-crude antigens, the antigenic epitope(s) of the target protein is not belonged to the carbohydrate part. The LC-MS/MS analysis of the selected protein band was identified as the myosin head of Ov. Myosin head (MH) is the head domain of myosin that facilitates the contraction of the parasite muscles, thus contributing to the diverse muscular activities exhibited by flukes. The binding efficacy of 222 KD rMH and Ov crude antigen were compared in mAb-ELISA at the same protein concentration. The results showed that the immune-reactivity of rMH against an in-house mAb was comparable with, or slightly better than, that of the crude Ov antigen. Moreover, *in silico* analysis confirmed the binding capacity between the monoclonal antibody and the rMH. Therefore, the rMH protein can be used as a new effective molecule substitute for Ov crude antigen to reduce the use of laboratory animals required to produce Ov worms.

Several muscle-associated proteins, such as myosin, actin, paramyosin, and tropomyosin, have been identified in trematodes. Detection of myosin in extracts of *Fasciola hepatica* was recognized in western blots using the anti-*T. solium* myosin type II antibody [30]. Myosin immunoreactivity was detected in the somatic muscle tissues as well as in the muscle of the oral sucker and uterus of *F. hepatica*. Since the immunoreactivity was confined to muscle tissue, it is likely that the antibody recognizes a common epitope that belongs to a type II myosin of cestodes and trematodes [30]. Myosin has been detected on the surface of *Schistosoma mansoni* [31]. Moreover, the recombinant polypeptides of myosin heavy chain show experimental promise as vaccine candidates, although this vaccine has not progressed to human trials yet [32]. Furthermore, myosin can be targeted by the medication praziquantel, which is employed to treat schistosomiasis [33]. Muscle proteins such as myosin could serve as potential targets for the development of an anti-schistosomal vaccine or chemotherapy [34, 35].

The results in this study raised several interesting points that required consideration. First, based on the results in this study, whether the antigen detected in urine or feces in OV-infected individuals consisted of OV myosin head or not required a confirmatory study. Second, whether the rMH described in this study has similar properties to the native myosin head molecule required additional investigations as there could be multiple isoforms due to post-translational modification and protein preparation. Lastly, the isolation and characterization of target molecules in urine of OV-infected individuals recognized by the current mAb deserved more study as it is important in the interpretation of the OV antigen profiles before and after treatment by praziquantel [36]. The information regarding OV-specific antigen detected in urine as well as feces, which originated from worms in the biliary system, is essential for our understanding of the OV antigen pathway and improvement of OV diagnostics.

Our study first focused on the identification and characterization of the myosin head, a specific molecule targeted by a mAb against the liver fluke, *O. viverrini*. Application of rMH protein as a substitute for crude Ov antigen will save costs, time, and minimize the requirement for animal use in the production of Ov crude antigens. Moreover, the insights gained from our experiments may contribute to the development of future Ov vaccine candidates. Additional research is required to clarify this issue.

**Acknowledgments**

This research was supported by the National Research Council of Thailand (NRCT) via Cholangiocarcinoma Research Institute, Khon Kaen University and the Fundamental Fund of Khon Kaen University from the National Science, Research and Innovation Fund (NSRF). We would like to acknowledge Prof. Yukifumi Nawa, for editing the MS via Publication Clinic KKU, Thailand.

**References**

1.	Thinkhamrop K, Khuntikeo N, Laohasiriwong W, Chupanit P, Kelly M, Suwannatrai AT. Association of comorbidity between *Opisthorchis viverrini* infection and diabetes mellitus in the development of cholangiocarcinoma among a high-risk population, northeastern Thailand. PLoS Negl Trop Dis. 2021;15(9):e0009741. doi: 10.1371/journal.pntd.0009741.

2.	Pinlaor S, Hiraku Y, Ma N, Yongvanit P, Semba R, Oikawa S, et al. Mechanism of NO-mediated oxidative and nitrative DNA damage in hamsters infected with *Opisthorchis* *viverrini*: a model of inflammation-mediated carcinogenesis. Nitric Oxide. 2004;11(2):175-83. doi: 10.1016/j.niox.2004.08.004.

3.	Pinlaor S, Prakobwong S, Boonmars T, Wongkham C, Pinlaor P, Hiraku Y. Effect of praziquantel treatment on the expression of matrix metalloproteinases in relation to tissue resorption during fibrosis in hamsters with acute and chronic *Opisthorchis* *viverrini* infection. Acta Trop. 2009;111(2):181-91. doi: 10.1016/j.actatropica.2009.04.011.

4.	Chamadol N, Pairojkul C, Khuntikeo N, Laopaiboon V, Loilome W, Sithithaworn P, et al. Histological confirmation of periductal fibrosis from ultrasound diagnosis in cholangiocarcinoma patients. J Hepatobiliary Pancreat Sci. 2014;21(5):316-22. doi: 10.1002/jhbp.64.

5.	Jamnongkan W, Lebrilla CB, Barboza M, Techasen A, Loilome W, Sithithaworn P, et al. Discovery of Serotransferrin Glycoforms: Novel Markers for Diagnosis of Liver Periductal Fibrosis and Prediction of Cholangiocarcinoma. Biomolecules. 2019;9(10). doi: 10.3390/biom9100538.

6.	Lovis L, Mak TK, Phongluxa K, Soukhathammavong P, Sayasone S, Akkhavong K, et al. PCR Diagnosis of *Opisthorchis viverrini* and Haplorchis taichui Infections in a Lao Community in an area of endemicity and comparison of diagnostic methods for parasitological field surveys. J Clin Microbiol. 2009;47(5):1517-23. doi: 10.1128/JCM.02011-08.

7.	Intapan PM, Thanchomnang T, Lulitanond V, Pongsaskulchoti P, Maleewong W. Rapid molecular detection of *Opisthorchis viverrini* in human fecal samples by real-time polymerase chain reaction. Am J Trop Med Hyg. 2009;81(5):917-20. doi: 10.4269/ajtmh.2009.09-0275.

8.	Aung WPP, Htoon TT, Tin HH, Thinn KK, Sanpool O, Jongthawin J, et al. First report and molecular identification of *Opisthorchis viverrini* infection in human communities from Lower Myanmar. PLoS One. 2017;12(5):e0177130. doi: 10.1371/journal.pone.0177130.

9.	Wongratanacheewin S, Pumidonming W, Sermswan RW, Maleewong W. Development of a PCR-based method for the detection of *Opisthorchis viverrini* in experimentally infected hamsters. Parasitology. 2001;122(Pt 2):175-80. doi: 10.1017/s0031182001007235.

10.	Le TH, Van De N, Blair D, Sithithaworn P, McManus DP. Clonorchis sinensis and *Opisthorchis viverrini*: development of a mitochondrial-based multiplex PCR for their identification and discrimination. Exp Parasitol. 2006;112(2):109-14. doi: 10.1016/j.exppara.2005.09.012.

11.	Worasith C, Kamamia C, Yakovleva A, Duenngai K, Wangboon C, Sithithaworn J, et al. Advances in the Diagnosis of Human Opisthorchiasis: Development of *Opisthorchis* *viverrini* Antigen Detection in Urine. PLoS Negl Trop Dis. 2015;9(10):e0004157. doi: 10.1371/journal.pntd.0004157.

12.	Worasith C, Wangboon C, Duenngai K, Kiatsopit N, Kopolrat K, Techasen A, et al. Comparing the performance of urine and copro-antigen detection in evaluating *Opisthorchis* *viverrini* infection in communities with different transmission levels in Northeast Thailand. PLoS Negl Trop Dis. 2019;13(2):e0007186. doi: 10.1371/journal.pntd.0007186.

13.	Worasith C, Sithithaworn J, Wongphutorn P, Homwong C, Khongsukwiwat K, Techasen A, et al. Accuracy of a new rapid diagnostic test for urinary antigen detection and assessment of drug treatment in opisthorchiasis. Infect Dis Poverty. 2023;12(1):102. doi: 10.1186/s40249-023-01162-4.

14.	Altschul SF, Gish W, Miller W, Myers EW, Lipman DJ. Basic local alignment search tool. J Mol Biol. 1990;215(3):403-10. doi: 10.1016/S0022-2836(05)80360-2.

15.	Watwiengkam N, Sithithaworn J, Duenngai K, Sripa B, Laha T, Johansen MV, et al. Improved performance and quantitative detection of copro-antigens by a monoclonal antibody based ELISA to diagnose human opisthorchiasis. Acta Trop. 2013;128(3):659-65. doi: 10.1016/j.actatropica.2013.09.012.

16.	Siripanthong S, Techasen A, Nantasenamat C, Malik AA, Sithithaworn P, Leelayuwat C, et al. Production and characterization of antibody against *Opisthorchis* *viverrini* via phage display and molecular simulation. PLoS One. 2021;16(3):e0248887. doi: 10.1371/journal.pone.0248887.

17.	Varadi M, Anyango S, Deshpande M, Nair S, Natassia C, Yordanova G, et al. AlphaFold Protein Structure Database: massively expanding the structural coverage of protein-sequence space with high-accuracy models. Nucleic Acids Res. 2022;50(D1):D439-D44. doi: 10.1093/nar/gkab1061.

18.	Pierce BG, Wiehe K, Hwang H, Kim BH, Vreven T, Weng Z. ZDOCK server: interactive docking prediction of protein-protein complexes and symmetric multimers. Bioinformatics. 2014;30(12):1771-3. doi: 10.1093/bioinformatics/btu097.

19.	Jurrus E, Engel D, Star K, Monson K, Brandi J, Felberg LE, et al. Improvements to the APBS biomolecular solvation software suite. Protein Sci. 2018;27(1):112-28. doi: 10.1002/pro.3280.

20.	Maier JA, Martinez C, Kasavajhala K, Wickstrom L, Hauser KE, Simmerling C. ff14SB: Improving the Accuracy of Protein Side Chain and Backbone Parameters from ff99SB. J Chem Theory Comput. 2015;11(8):3696-713. doi: 10.1021/acs.jctc.5b00255.

21.	Mark P, Nilsson L. Structure and Dynamics of the TIP3P, SPC, and SPC/E Water Models at 298 K. J Phys Chem A. 2001;105(43):9954-60. doi: 10.1021/jp003020w.

22.	Case DA, Aktulga HM, Belfon K, Ben-Shalom IY, Berryman JT, Brozell SR, et al. Amber 2023. University of California, San Francisco. 2023.

23.	Berendsen HJC, Postma JPM, van Gunsteren WF, DiNola A, JR. H. Molecular dynamics with coupling to an external bath. J Chem Phys. 1984;81(8):3684-90. doi: 10.1063/1.448118.

24.	Uberuaga BP, Anghel M, AF. V. Synchronization of trajectories in canonical molecular-dynamics simulations: Observation, explanation, and exploitation. J Chem Phys. 2004;120(14):6363-74. doi: 10.1063/1.1667473.

25.	Elber R, Ruymgaart AP, B H. SHAKE parallelization. EPJ ST. 2011;200(1):211-23. doi: 10.1140/epjst/e2011-01525-9.

26.	Darden TA, York DM, LG P. Particle mesh Ewald: An N⋅log(N) method for Ewald sums in large systems. J Chem Phys. 1993;98:10089-92. doi: org/10.1063/1.464397.

27.	Young ND, Nagarajan N, Lin SJ, Korhonen PK, Jex AR, Hall RS, et al. The Opisthorchis viverrini genome provides insights into life in the bile duct. Nat Commun. 2014;5:4378. doi: 10.1038/ncomms5378.

28.	Senawong G, Laha T, Loukas A, Brindley PJ, Sripa B. Cloning, expression, and characterization of a novel Opisthorchis viverrini calcium-binding EF-hand protein. Parasitol Int. 2012;61(1):94-100. doi: 10.1016/j.parint.2011.07.012.

29.	Suttiprapa S, Loukas A, Laha T, Wongkham S, Kaewkes S, Gaze S, et al. Characterization of the antioxidant enzyme, thioredoxin peroxidase, from the carcinogenic human liver fluke, *Opisthorchis viverrini*. Mol Biochem Parasitol. 2008;160(2):116-22. doi: 10.1016/j.molbiopara.2008.04.010.

30.	Kumar D, McGeown JG, Reynoso-Ducoing O, Ambrosio JR, Fairweather I. Observations on the musculature and isolated muscle fibres of the liver fluke, *Fasciola* *hepatica*. Parasitology. 2003;127(Pt 5):457-73. doi: 10.1017/s0031182003003925.

31.	Deng J, Gold D, LoVerde PT, Fishelson Z. Inhibition of the complement membrane attack complex by *Schistosoma* *mansoni* paramyosin. Infect Immun. 2003;71(11):6402-10. doi: 10.1128/IAI.71.11.6402-6410.2003.

32.	Jones MK, Gobert GN, Zhang L, Sunderland P, McManus DP. The cytoskeleton and motor proteins of human schistosomes and their roles in surface maintenance and host-parasite interactions. Bioessays. 2004;26(7):752-65. doi: 10.1002/bies.20058.

33.	Gnanasekar M, Salunkhe AM, Mallia AK, He YX, Kalyanasundaram R. Praziquantel affects the regulatory myosin light chain of *Schistosoma* *mansoni*. Antimicrob Agents Chemother. 2009;53(3):1054-60. doi: 10.1128/AAC.01222-08.

34.	Sulbaran G, Alamo L, Pinto A, Marquez G, Mendez F, Padron R, et al. An invertebrate smooth muscle with striated muscle myosin filaments. Proc Natl Acad Sci U S A. 2015;112(42):E5660-8. doi: 10.1073/pnas.1513439112.

35.	Qokoyi NK, Masamba P, Kappo AP. Proteins as Targets in Anti-Schistosomal Drug Discovery and Vaccine Development. Vaccines (Basel). 2021;9(7). doi: 10.3390/vaccines9070762.

36.	Worasith C, Wangboon C, Kopolrat KY, Homwong C, Sithithaworn J, Techasen A, et al. Application of urine antigen assay to evaluate outcomes of praziquantel treatment and reinfection in opisthorchiasis in northeast Thailand. Trans R Soc Trop Med Hyg. 2020;114(10):751-61. doi: 10.1093/trstmh/traa057.

**Supporting information**

**S1 File. LC-MS/MS analysis data sheet of Ov myosin head protein from Ov crude antigens.**

**S2 File.** **LC-MS/MS analysis data sheet of the Ov myosin head recombinant protein (222 KD) from the myosin head gene inserted pET-15b vector-transfected *E. coli* stain BL21 (DE3) crude extract.**
