# Supplementary material for: Identification and characterization of a target antigen recognized by the monoclonal antibody against Opisthorchis viverrini
Source: PLoS One. 2025 May 29;20(5):e0324137. doi: 10.1371/journal.pone.0324137 (PMC12121735; doi:10.1371/journal.pone.0324137)
Supplement: Attachment — Submitted filename: Response to Revewers.docx [file pone.0324137.s008.docx]

**Point-by-Point Responses to the Editor’s and Reviewer’s Comments**

We express our gratitude to the editor and reviewers for their constructive comments and suggestions to improve our manuscript. We appreciate the opportunity to revise our manuscript and have made the necessary amendments accordingly.

1. Double check the MS for English usage, and typos.

The authors’ response to the editor’s Comment #1: We have carefully examined the English usage and corrected any typographical errors in this manuscript. Additionally, the Quillbot Premium, an online software, was utilized to verify the grammar and spelling in the revised manuscript.

2. Demonstrate the antigen recognition pattern, and the usefulness in Ov and non-Ov samples.

The authors’ response to the editor’s Comment #2:

The antigen recognition pattern was further analyzed by B cell epitope prediction. The results aligned well with MD simulations data, identifying the epitope between residues 461-628. The results have been incorporated into the revised manuscript (Line 311-316 and 406-407).

The usefulness of the monoclonal antibody KKU505 in distinguishing urine samples from Ov and non-Ov subjects was clearly demonstrated by our group [1]. The monoclonal antibody exhibited complete specificity for urine samples from subjects infected with Ov (81%), with minimal cross-reactivity observed in samples from individuals with *S. stercoralis* infections (3.57%) and hookworm infections (10%). The information has been added in the “Introduction” section of the revised manuscript (Line 88-93)

**Reference**

1. Worasith C, Kamamia C, Yakovleva A, Duenngai K, Wangboon C, Sithithaworn J, et al. Advances in the Diagnosis of Human Opisthorchiasis: Development of Opisthorchis viverrini Antigen Detection in Urine. PLoS Negl Trop Dis. 2015;9(10):e0004157. doi: 10.1371/journal.pntd.0004157.

3. Clear the points raised by the Reviewers, especially the additional experiments required.

The authors’ response to the editor’s Comment #3: We addressed all comments and suggestions from the reviewers as described below.

**Reviewer #1:** The manuscript entitled "Identification and characterization of a target antigen recognized by the monoclonal antibody against Opisthorchis viverrini" by Techasen and coworkers had been reviewed. Generally, the study in the manuscript dealt with an interesting topic. The study format was well designed, and the data are valuable for publication. I do feel that it has merit but does not fully meet the publication standard as it currently stands. The authors can submit an improved version of the manuscript that addresses the points raised as follows as well as in the attached review report.

1. The language of the manuscript still needs to be polished to improve accuracy throughout the whole manuscript, especially in the results and discussion sections (see my comments in the attached review report).

The authors’ response to Comment #1: We sincerely appreciate your thorough review and valuable recommendations for enhancing the quality of our manuscript. The manuscript has been revised in accordance with your suggestions, as detailed in the marked version.

2. How is the affinity between the mAb and Ov myosin?

The authors’ response to Comment #2: The equilibrium dissociation constant (K_D_) indicates the binding affinity between monoclonal antibodies (mAb KKU505) and their target protein. A lower K_D_ value indicates a greater binding affinity, whereas a higher K_D_ value signifies a weaker interaction. The raw data presented in Fig. 3B were utilized to determine the K_D_ values between the mAb and crude-Ov (5.298 µg/mL), as well as between the mAb and recombinant full-length Ov myosin heavy chain (rMHC) protein (1.329 µg/mL), using GraphPad Prism 9 software (Fig R1). The results indicated that rMHC exhibited a higher affinity to the mAb compared to crude-Ov antigens. The aforementioned information has been included in the revised manuscript (Line 279-282 and 374-379)

**Figure R1** The binding affinity analysis of mAb KKU505 with various protein concentrations from crude-Ov antigens and rMHC from GraphPad Prism 9 software.

3. Does the mAb recognize the host myosin? I would like to suggest including a homology analysis on the parasite myosin and host orthologs in the manuscript, especially focusing on the binding sites predicted by the molecular modeling.
The authors’ response to Comment #3: Molecular docking analysis was conducted on the myosin head domain of Ov- myosin heavy chain (Ov myosin head) and the human myosin heavy chain 7 (human myosin head). The molecular docking results suggested that Ov-myosin head had a higher binding affinity to the mAb compared to the host myosin, with docking scores of -245.6 and -224.85, respectively (Fig. R2). Furthermore, the urine antigen levels decreased following praziquantel treatment, as identified by mAb ELISA and OV-RDT [1, 2]. A one-year post-treatment follow-up revealed the presence of antigen in urine, which correlated with fecal examination results. This indicates that Ov antigen in urine is associated with Ov infection. Therefore, it is improbable that the monoclonal antibody is interacting with host antigens, such as heavy chain 7.

**Figure R2** The molecular docking analysis of the binding interactions between the mAb and Ov myosin head, as well as between the mAb and host myosin.

**Reference**

1. Worasith C, Wangboon C, Kopolrat KY, Homwong C, Sithithaworn J, Techasen A, et al. Application of urine antigen assay to evaluate outcomes of praziquantel treatment and reinfection in opisthorchiasis in northeast Thailand. Trans R Soc Trop Med Hyg. 2020;114(10):751-61. doi: 10.1093/trstmh/traa057.

2. Worasith C, Sithithaworn J, Wongphutorn P, Homwong C, Khongsukwiwat K, Techasen A, et al. Accuracy of a new rapid diagnostic test for urinary antigen detection and assessment of drug treatment in opisthorchiasis. Infect Dis Poverty. 2023;12(1):102. doi: 10.1186/s40249-023-01162-4.

4. The discussion section can still be deepened. Can authors explain why the mAb cannot recognize the trimmed myosin (spanning nt. 120-2,256 encoding 712 aa) in the ELISA test (Fig 2B).

The authors’ response to Comment #3: A more detailed explanation regarding the inability of the mAb to recognize the trimmed myosin has been incorporated into the revised manuscript (Line 430-439): “Moreover, in silico analysis confirmed the binding capacity between the monoclonal antibody and the Ov myosin head domain. The molecular docking results demonstrated that the Ov myosin head domain employs amino acid residues from Asn268 to Gly628 for interaction with the mAb KKU505. The amino acid residues from 461 to 628 were also predicted to have an antigenicity to B cells. The recombinant full-length Ov myosin heavy chain and partial Ov myosin head were performed. The partial Ov myosin head was constructed exclusively in the N-terminal region (712 amino acids) to enhance the protein production and preparation efficiency. Nevertheless, it demonstrated negative reactivity with the monoclonal antibody. These results suggest that the partial myosin head protein expression may lack proper conformation, leading to diminished immunoreactivity.”

**Reviewer #2:** This manuscript primarily focuses on identifying the target antigen in Opisthorchis viverrini recognized by the monoclonal antibody (clone KKU505) developed in previous research. However, there are still several significant areas where additional experiments and further clarification are needed.

1. Can the KKU505 monoclonal antibody detect the ES products of *O. viverrini*? If it can, the authors should conduct target identification of KKU505 within the ES products of *O. viverrini*.

The authors’ response to Comment #1: The mAb clone KKU505 exhibits reactivity to ES and somatic products of *O. viverrini*, as demonstrated by mAb-ELISA, according to Worasith C. and colleagues [1]. We sought to identify the target antigen in the ES protein; however, the results were inconclusive. This necessitates additional investigation in the future.

**Reference**

1. Worasith C, Kamamia C, Yakovleva A, Duenngai K, Wangboon C, Sithithaworn J, et al. Advances in the Diagnosis of Human Opisthorchiasis: Development of Opisthorchis viverrini Antigen Detection in Urine. PLoS Negl Trop Dis. 2015;9(10):e0004157. doi: 10.1371/journal.pntd.0004157.

2. The Western blot shows a broad area of reactivity. Why did the authors select only two distinct bands for analysis? Why not include the entire reactive region?

The authors’ response to Comment #2: We attempted to identify additional bands; however, the MS/MS results yielded low accuracy scores. As a result, these results have been excluded from the manuscript.

3. Since 1D gel-mass spectrometry may lack accuracy, the authors should use additional methods in parallel, such as immunoprecipitation or 2DE-immunomics.

The authors’ response to Comment #3: In agreement with the reviewer’s comments, we previously interacted the mAb with protein G or protein A beads and as a primary antibody in immunoprecipitation and 2DE-immunomics. Despite extensive optimization efforts, the results remained inconclusive. Consequently, we have excluded them from the manuscript.

4. What is the specific epitope recognized by the KKU505 monoclonal antibody?

The authors’ response to Comment #4: The *in-silico* analysis confirmed the binding capacity between the monoclonal antibody and the Ov myosin head protein. The molecular docking results indicated that the Ov myosin head protein interaction with the mAb through amino acid residues spanning Asn268 to Gly628. These results, along with the capture-ELISA results, showed that the appropriate conformation of the Ov myosin head protein allows the 3D structure of the peptide between Asn268 and Gly628 to fit the mAb.

B-cell epitope prediction was performed by capturing spatial clustering property
of the epitopes using graph attention network via **EpiGraph (**[**http://epigraph.kaist.ac.kr/**](http://epigraph.kaist.ac.kr/)**)**, which was developed by Choi, S. and Kim, D. [1]. The results are consistent with molecular docking, and the epitope was identified between residues 461-628. This analysis was incorporated in the revised manuscript (Line 311-316 and 406-407) and S4 File. The results of residues 461-628 are also shown below.

> Ov myosin head: amino acid residue 461-628

FDENGFEQICINYTNERLQQFFNHHMFVLEQEEYKREKIQWTFIDFGMDLQACIDLIEKPMGILSILEEECIVPKATDQTFLSKLYDNHLGKSPNFTKPKPPKAGQKEAHFELHHYAGSVPYTITGWLEKNKDPLNDTVVNLLGGSKDALVSQLFVPVVAESGKKTKG

**Reference**

1. Choi S, Kim D. B cell epitope prediction by capturing spatial clustering property of the epitopes using graph attention network. Sci Rep. 2024;14(1):27496. doi: 10.1038/s41598-024-78506-z

5. For the LC-MS/MS results, please provide a complete list of peptides identified through mass spectrometry, in a supplementary table to ensure unbiased selection.

The authors’ response to Comment #4: The LC-MS/MS results have been updated with the comprehensive protein list obtained from the MS/MS analysis in the revised manuscript (S1 File).

6. Ideally, this study should include identification of the targets specifically recognized by the KKU505 monoclonal antibody in feces and urine

The authors’ response to Comment #5: Since the specific target antigen recognized by the mAb KKU505 clone remains unidentified, this study represents an initial step to elucidate the antigen components in the worms, potentially uncovering the Ov antigen detected in urine and feces of opisthorchiasis patients. As suggested by the reviewers, future work is needed to identify the specific target antigens recognized by KKU 505.
